# Parton distributions from high-precision collider data: NNPDF Collaboration

**DOI:** 10.1140/epjc/s10052-017-5199-5

**Published:** 2017-10-04

**Authors:** Richard D. Ball, Valerio Bertone, Stefano Carrazza, Luigi Del Debbio, Stefano Forte, Patrick Groth-Merrild, Alberto Guffanti, Nathan P. Hartland, Zahari Kassabov, José I. Latorre, Emanuele R. Nocera, Juan Rojo, Luca Rottoli, Emma Slade, Maria Ubiali

**Affiliations:** 1 The Higgs Centre for Theoretical Physics, University of Edinburgh, JCMB, KB, Mayfield Rd, Edinburgh, EH9 3JZ Scotland; 2 Department of Physics and Astronomy, VU University, 1081 HV Amsterdam, The Netherlands; 3 Nikhef Theory Group, Science Park 105, 1098 XG Amsterdam, The Netherlands; 4 Theoretical Physics Department, CERN, 1211 Geneva, Switzerland; 5 Tif Lab, Dipartimento di Fisica, Università di Milano, Milano, Italy; 6 INFN, Sezione di Milano, Via Celoria 16, 20133 Milano, Italy; 7 Dipartimento di Fisica, Università di Torino, Turin, Italy; 8 INFN, Sezione di Torino, Via P. Giuria 1, 10125 Turin, Italy; 9 Departament de Física Quàntica i Astrofísica, Universitat de Barcelona, Diagonal 645, 08028 Barcelona, Spain; 10 Center for Quantum Technologies, National University of Singapore, Singapore, Singapore; 11 Rudolf Peierls Centre for Theoretical Physics, 1 Keble Road, University of Oxford, OX1 3NP Oxford, UK; 12 Cavendish Laboratory, HEP Group, University of Cambridge, J.J. Thomson Avenue, Cambridge, CB3 0HE UK

## Abstract

We present a new set of parton distributions, NNPDF3.1, which updates NNPDF3.0, the first global set of PDFs determined using a methodology validated by a closure test. The update is motivated by recent progress in methodology and available data, and involves both. On the methodological side, we now parametrize and determine the charm PDF alongside the light-quark and gluon ones, thereby increasing from seven to eight the number of independent PDFs. On the data side, we now include the D0 electron and muon *W* asymmetries from the final Tevatron dataset, the complete LHCb measurements of *W* and *Z* production in the forward region at 7 and 8 TeV, and new ATLAS and CMS measurements of inclusive jet and electroweak boson production. We also include for the first time top-quark pair differential distributions and the transverse momentum of the *Z* bosons from ATLAS and CMS. We investigate the impact of parametrizing charm and provide evidence that the accuracy and stability of the PDFs are thereby improved. We study the impact of the new data by producing a variety of determinations based on reduced datasets. We find that both improvements have a significant impact on the PDFs, with some substantial reductions in uncertainties, but with the new PDFs generally in agreement with the previous set at the one-sigma level. The most significant changes are seen in the light-quark flavor separation, and in increased precision in the determination of the gluon. We explore the implications of NNPDF3.1 for LHC phenomenology at Run II, compare with recent LHC measurements at 13 TeV, provide updated predictions for Higgs production cross-sections and discuss the strangeness and charm content of the proton in light of our improved dataset and methodology. The NNPDF3.1 PDFs are delivered for the first time both as Hessian sets, and as optimized Monte Carlo sets with a compressed number of replicas.

## Introduction

A precise understanding of parton distributions [1–3] (PDFs) has played a major role in the discovery of the Higgs boson and will be a key ingredient in searches for new physics at the LHC [4]. In recent years PDF sets of a new generation [5–11] have been developed for use at the LHC Run II. Some of these have been used in the construction of the PDF4LHC15 combined sets, recommended for new physics searches and for the assessment of PDF uncertainties on precision observables [12]. These PDF4LHC15 sets are obtained by means of statistical combination of the three global sets [5–7]: this is justified by the improved level of agreement in the global determinations, with differences between them largely consistent with statistical fluctuation.

Despite these developments, there remains a need for improvements in the precision and reliability of PDF determinations. Precision measurements at the LHC, such as in the search for new physics through Higgs coupling measurements, will eventually require a systematic knowledge of PDFs at the percent level in order to fully exploit the LHC’s potential. The NNPDF3.0 PDF set [5], which is one of the sets entering the PDF4LHC15 combination, is unique in being a PDF set based on a methodology systematically validated by means of closure tests, which ensure the statistical consistency of the procedure used to extract the PDFs from data. The goal of this paper is to present NNPDF3.1, an update of the NNPDF3.0 set, and a first step towards PDFs with percent-level uncertainties. Two directions of progress are required in order to reach this goal, the motivation for an update being accordingly twofold.

On the one hand, bringing the precision of PDFs down to the percent level requires a larger and more precise dataset, with correspondingly precise theoretical predictions. In the time since the release of NNPDF3.0, a significant number of new experimental measurements have become available. From the Tevatron, we now have the final measurements of the *W* boson asymmetries with the electron and muon final states based upon the complete Run II dataset [13, 14]. At the LHC, the ATLAS, CMS and LHCb experiments have released a wide variety of measurements on inclusive jet production, gauge boson production and top production. Finally, the combined legacy measurements of DIS structure functions from HERA have also become available [9]. In parallel with the experimental developments, an impressive number of new high-precision QCD calculations of hadron collider processes with direct sensitivity to PDFs have recently been completed, enabling their use in the determination of PDFs at NNLO. These include differential distributions in top-quark pair production [15, 16], the transverse momentum of the *Z* and *W* bosons [17, 18], and inclusive jet production [19, 20], for all of which precision ATLAS and CMS datasets are available.

All of these new datasets and calculations have been incorporated into NNPDF3.1. The inclusion of the new data presents new challenges. Given the large datasets on which some of these measurements are based, uncorrelated experimental uncertainties are often at the permille level. Achieving a good fit then requires an unprecedented control of both correlated systematics and of the numerical accuracy of theoretical predictions.

On the other hand, with uncertainties at the percent level, accuracy issues related to theoretical uncertainties hitherto not included in PDF determinations become relevant. Whereas the comprehensive inclusion of theoretical uncertainties in PDF determination will require further study, we have recently argued that a significant source of theoretical bias arises from the conventional assumption that charm is generated entirely perturbatively from gluons and light quarks. A methodology which allows for the inclusion of a parametrized heavy-quark PDFs within the FONLL matched general-mass variable-flavor number scheme has been developed [21, 22], and implemented in an NNPDF PDF determination [23]. It was found that when the charm PDF is parametrized and determined from the data alongside the other PDFs, much of the uncertainty related to the value of the charm mass becomes part of the standard PDF uncertainty, while any bias related to the assumption that the charm PDF is purely perturbative is eliminated [23]. In NNPDF3.1 charm is therefore parametrized as an independent PDF, in an equivalent manner to light quarks and the gluon. We will show that this leads to improvements in fit quality without an increase in uncertainty, and that it stabilizes the dependence of PDFs on the charm mass, all but removing it in the light-quark PDFs.

The NNPDF3.1 PDF sets are released at LO, NLO, and NNLO accuracy. For the first time, all NLO and NNLO PDFs are delivered both as Hessian sets and as Monte Carlo replicas, exploiting recent powerful methods for the construction of optimal Hessian representations of PDFs [24]. Furthermore, and also for the first time, the default PDF sets are provided as compressed Monte Carlo sets [25]. Therefore, despite being presented as sets of only 100 Monte Carlo replicas, they exhibit many of the statistical properties of a much larger set, reducing observable computation time without loss of information. A further improvement in computational efficiency can be obtained by means of the SM-PDF tool [26], which allows for the selection of optimal subsets of Hessian eigenvectors for the computation of uncertainties on specific processes or classes of processes, and which is available as a web interface [27], now also including the NNPDF3.1 sets. A variety of PDF sets based on subsets of data are also provided (as standard 100 replica Monte Carlo sets), which may be useful for specific applications such as new physics searches, or measurements of standard model parameters.

The outline of this paper is as follows. First, in Sect. 2 we discuss the experimental aspects and the relevant theoretical issues of the new datasets. We then turn in Sect. 3 to a detailed description of the baseline NNPDF3.1 PDF sets, with a specific discussion of the impact of methodological improvements, specifically the fact that the charm PDF is now independently parametrized and determined like all other PDFs. In Sect. 4 we discuss the impact of the new data by comparing PDF sets based upon various data subsets, and also discuss PDF sets based on more conservative data subsets. In Sect. 5 we summarize the status of uncertainties on PDFs and luminosities, and specifically discuss the strange and charm content of the proton in light of our results, and present first phenomenological studies at the LHC. Finally, a summary of the PDFs being delivered in various formats is provided in Sect. 6, together with links to repositories whence more detailed sets of plots may be downloaded.

## Experimental and theoretical input

The NNPDF3.1 PDF sets include a wealth of new experimental data. We have augmented our dataset with improved determinations of observables already included in NNPDF 3.0 (such as *W* and *Z* rapidity distributions) as well as two new processes: top-quark differential distributions, and the *Z* transverse momentum distribution, which is included for the first time in a global PDF determination.

In this section we discuss the NNPDF3.1 dataset in detail. After a general overview, each observable will be examined: we describe the individual measurements, and address specific theoretical and phenomenological issues related to their inclusion, particularly in relation to the use of recent NNLO results.

In NNPDF3.1 only LHC data from Run I, taken at center-of-mass energies of 2.76, 7 and 8 TeV (with one single exception), are included. The more recent 13 TeV dataset is reserved for phenomenological comparison purposes in Sect. 5. Available and upcoming LHC Run II data at 13 TeV will be part of future NNPDF releases.

### Experimental data: general overview

The NNPDF3.0 global analysis involved data from deep-inelastic scattering (DIS) experiments, fixed-target Drell–Yan data, and collider measurements from the Tevatron and LHC. The fixed-target and collider DIS datasets included measurements from NMC [28, 29], BCDMS [30, 31] and SLAC [32]; the combined HERA-I inclusive structure function dataset [33] and HERA-II inclusive measurements from H1 and ZEUS [34–37]; the HERA combined measurements of the charm production cross-section $\sigma _{c}^\mathrm{NC}$ [38]; CHORUS inclusive neutrino DIS [39], and NuTeV dimuon production data [40, 41]. From the Tevatron, CDF [42] and D0 [43] *Z* rapidity distributions; and CDF [44] Run-II one-jet inclusive cross-sections were used. Constraints from fixed-target Drell–Yan came from the E605 [45] and E866 [46–48] experiments. LHC measurements included electroweak boson production data from ATLAS [49–51], CMS [52–54] and LHCb [55, 56]; one-jet inclusive cross-sections from ATLAS [57, 58] and CMS [59]; the differential distributions for *W* production in association with charm quarks from CMS [60]; and total cross-section measurements for top-quark pair production data from ATLAS and CMS at 7 and 8 TeV [61–66].

**Table 1:** Deep-inelastic scattering data included in NNPDF3.1. The EMC $F_2^c$ data are in brackets because they are only included in a dedicated set but not in the default dataset. New datasets, not included in NNPDF3.0, are denoted (*). The kinematic range covered in each variable is given after cuts are applied. The total number of DIS data points after cuts is 3102 / 3092 for the NLO/NNLO PDF determinations (not including the EMC $F_2^c$ data)

Experiment	Obs.	Ref.	$N_{\mathrm{dat}}$	*x* range	*Q* range (GeV)	Theory
NMC	$F_2^d/F_2^p$	[28]	260 (121/121)	$0.012\le x \le 0.68$	$2.1 \le Q \le 10$	APFEL
	$\sigma ^\mathrm{NC,p}$	[29]	292 (204/204)	$0.012 \le x \le 0.50$	$1.8 \le Q \le 7.9$	
SLAC	$F_2^p$	[32]	211 (33/33)	$0.14\le x \le 0.55$	$1.9 \le Q \le 4.4$	APFEL
	$F_2^d$	[32]	211 (34/34)	$0.14 \le x \le 0.55$	$1.9\le Q \le 4.4$	
BCDMS	$F_2^p$	[30]	351 (333/333)	$0.07 \le x \le 0.75$	$2.7\le Q \le 15.1$	APFEL
	$F_2^d$	[31]	254 (248/248)	$0.07 \le x \le 0.75$	$3.0\le Q \le 15.1$	
CHORUS	$\sigma ^\mathrm{CC,\nu }$	[39]	607 (416/416)	$0.045 \le x \le 0.65$	$1.9\le Q \le 9.8$	APFEL
	$\sigma ^\mathrm{CC,\bar{\nu }}$	[39]	607 (416/416)	$0.045 \le x \le 0.65$	$1.9 \le Q \le 9.8$	
NuTeV	$\sigma _\nu ^{cc}$	[40, 41]	45 (39/39)	$0.02 \le x \le 0.33$	$2.0 \le Q \le 10.8$	APFEL
	$\sigma _{\bar{\nu }}^{cc}$	[40, 41]	45 (37/37)	$0.02 \le x \le 0.21$	$1.9 \le Q \le 8.3$	
HERA	$\sigma _{\mathrm{NC,CC}}^{p}$ (*)	[9]	1306 (1145/1145)	$4\cdot 10^{-5}\le x \le 0.65$	$1.87 \le Q \le 223$	APFEL
	$\sigma _{\mathrm{NC}}^{c}$	[38]	52 (47/37)	$7 \cdot 10^{-5} \le x \le 0.05$	$2.2 \le Q \le 45$	
	$F_2^b$ (*)	[67, 68]	29 (29/29)	$2\cdot 10^{-4}\le x \le 0.5$	$2.2 \le Q \le 45$	
EMC	[ $F_2^c$ ] (*)	[69]	21 (16/16)	$0.014\le x \le 0.44$	$2.1 \le Q \le 8.8$	APFEL

For NNPDF3.1 we have made a number of improvements to the NNPDF3.0 dataset. Firstly we have included the final datasets for several experiments which have now concluded, replacing superseded data in the NNPDF3.0 analysis. The HERA-I data and the H1 and ZEUS HERA-II inclusive structure functions have been replaced by the final HERA combination [9]. The HERA dataset has also been enlarged by the inclusion of H1 and ZEUS measurements of the bottom structure function $F_2^b(x,Q^2)$ [67, 68], which may prove useful in specific applications such as in the determination of the bottom quark mass $m_\mathrm{b}$. In order to perform dedicated studies of the charm content of the proton, we have constructed a PDF set also including the EMC measurements of charm structure functions at large *x* [69], which will be discussed in Sect. 5.3. However, these measurements are not included in the standard dataset. The legacy *W* lepton asymmetries from D0 using the complete Tevatron luminosity, both in the electron [14] and in the muon [13] channels, have been added. These precise weak gauge boson production measurements provide important information on the quark flavor separation at large *x*, as demonstrated in [70].

Aside from the updated legacy datasets, in NNPDF3.1 a large number of recent measurements from ATLAS, CMS and LHCb are included. For ATLAS, we now include the *Z* boson $(p_T^Z,y_Z)$ and $(p_T^Z,M_{ll})$ double-differential distributions measured at 8 TeV [71]; the inclusive $W^+$, $W^-$ and *Z* rapidity distributions at 7 TeV from the 2011 dataset [72], the top-quark pair production normalized $y_t$ distribution at 8 TeV [73]; total cross-sections for top-quark pair production at 7, 8 and 13 TeV [74, 75]; inclusive jet cross-sections at 7 TeV from the 2011 dataset [76]; and finally low-mass Drell–Yan $M_{ll}$ distributions at 7 TeV from the 2010 run [77]. The transverse momentum spectrum at 7 TeV (2011 dataset) [78] will be studied in Sect. 4.2 but it is not included in the default set. The total top cross-section is the only data point at 13 TeV which is included. For CMS, NNPDF3.1 includes the $W^+$ and $W^-$ rapidity distributions at 8 TeV [79], together with their cross-correlations; the inclusive jet production cross-sections at 2.76 TeV [80]; top-quark pair production normalized $y_{t\bar{t}}$ distributions at 8 TeV [81], total inclusive $t\bar{t}$ cross-sections at 7, 8 and 13 TeV [82]; the distribution of the *Z* boson double differentially in $(p_T,y_Z)$ at 8 TeV [83]. The double-differential distributions $(y_{ll},M_{ll})$ in Drell–Yan production at 8 TeV [84] will be studied in Sect. 4.8 below, but they are not included in the default PDF determination. For LHCb, NNPDF3.1 includes the complete 7 and 8 TeV measurements of inclusive *W* and *Z* production in the muon channel [85, 86], which supersede all previous measurements in the same final state.

**Table 2:** Same as Table 1 for the Tevatron fixed-target Drell–Yan and *W*, *Z* and jet collider data. The total number of Tevatron data points after cuts is 345 / 339 for NLO/NNLO fits

Exp.	Obs.	Ref.	$N_{\mathbf{dat}}$	$\hbox {Kin}_1$	$\hbox {Kin}_2$ (GeV)	Theory
E866	$\sigma _{\mathrm{DY}}^d/\sigma _{\mathrm{DY}}^p$	[48]	15 (15/15)	0.07 $\le y_{ll}\le 1.53$	$4.6 \le M_{ll}\le 12.9$	APFEL+Vrap
	$\sigma _{\mathrm{DY}}^p$	[46, 47]	184 (89/89)	$0 \le y_{ll}\le 1.36$	$4.5 \le M_{ll}\le 8.5$	APFEL+Vrap
E605	$\sigma _{\mathrm{DY}}^p$	[45]	119 (85/85)	$-0.2 \le y_{ll}\le 0.4$	$7.1 \le M_{ll}\le 10.9$	APFEL+Vrap
CDF	$d\sigma _Z/dy_Z$	[42]	29 (29/29)	$0 \le y_{ll}\le 2.9$	$66 \le M_{ll}\le 116$	Sherpa+Vrap
	$k_t$ incl jets	[87]	76 (76/76)	$0\le y_{\mathrm{jet}}\le 1.9$	$58 \le p_T^\mathrm{jet}\le 613$	NLOjet++
D0	$d\sigma _Z/dy_Z$	[43]	28 (28/28)	$0 \le y_{ll}\le 2.8$	$66 \le M_{ll}\le 116$	Sherpa+Vrap
	*W* electron asy (*)	[14]	13 (13/8)	$0 \le y_{e}\le 2.9$	$Q=M_W$	MCFM+FEWZ
	*W* muon asy (*)	[13]	10 (10/9)	$0 \le y_{\mu }\le 1.9$	$Q=M_W$	MCFM+FEWZ

**Table 3:** Same as Table 1, for ATLAS, CMS and LHCb data from the LHC Run I at $\sqrt{s}=2.76$ TeV, $\sqrt{s}=7$ TeV and $\sqrt{s}=8$ TeV. The ATLAS 7 TeV $Z\, p_T$ and CMS 2D DY 2012 are in brackets because they are only included in a dedicated study but not in the default PDF set. The total number of LHC data points after cuts is 848 / 854 for NLO/NNLO fits (not including ATLAS 7 TeV $Z\, p_T$ and CMS 2D DY 2012).

Exp.	Obs.	Ref.	$N_{\mathrm{\mathbf dat}}$	Kin$_1$	Kin$_2$ (GeV)	Theory
ATLAS	*W*, *Z* 2010	[49]	30 (30/30)	$0 \le |\eta _l| \le 3.2$	$Q=M_W, M_Z$	MCFM+FEWZ
	*W*, *Z* 2011 (*)	[72]	34 (34/34)	$0 \le |\eta _l| \le 2.3$	$Q=M_W, M_Z$	MCFM+FEWZ
	High-mass DY 2011	[50]	11 (5/5)	$0 \le |\eta _l| \le 2.1$	$116 \le M_{ll} \le 1500$	MCFM+FEWZ
	Low-mass DY 2011 (*)	[77]	6 (4/6)	$0 \le |\eta _l| \le 2.1$	$14 \le M_{ll} \le 56$	MCFM+FEWZ
	[$Z\, p_T$ 7 TeV $\left( p_T^Z,y_Z\right)$] (*)	[78]	64 (39/39)	$0 \le |y_{Z}| \le 2.5$	$30 \le p_T^Z \le 300$	MCFM+NNLO
	$Z\, p_T$ 8 TeV $\left( p_T^Z,M_{ll}\right)$ (*)	[71]	64 (44/44)	$12 \le M_{ll} \le 150$ GeV	$30 \le p_T^Z \le 900$	MCFM+NNLO
	$Z\, p_T$ 8 TeV $\left( p_T^Z,y_Z\right)$ (*)	[71]	120 (48/48)	$0.0\le |y_{Z}| \le 2.4$	$30\le p_{T}^Z \le 150$	MCFM+NNLO
	7 TeV jets 2010	[57]	90 (90/90)	$0 \le |y^\mathrm{jet}| \le 4.4$	$25 \le p_T^\mathrm{jet} \le 1350$	NLOjet++
	2.76 TeV jets	[58]	59 (59/59)	$0 \le |y^\mathrm{jet}| \le 4.4$	$20 \le p_T^\mathrm{jet} \le 200$	NLOjet++
	7 TeV jets 2011 (*)	[76]	140 (31/31)	$0 \le |y^\mathrm{jet}| \le 0.5$	$108 \le p_T^\mathrm{jet} \le 1760$	NLOjet++
	$\sigma _{\mathrm{tot}}(t\bar{t})$	[74, 75]	3 (3/3)	–	$Q=m_t$	top++
	$(1/\sigma _{t\bar{t}})d\sigma (t\bar{t})/y_t$ (*)	[73]	10 (10/10)	$0<|y_t|<2.5$	$Q=m_t$	Sherpa+NNLO
CMS	*W* electron asy	[52]	11 (11/11)	$0 \le |\eta _{\mathrm{e}}| \le 2.4$	$Q=M_W$	MCFM+FEWZ
	*W* muon asy	[53]	11 (11/11)	$0 \le |\eta _{\mu }| \le 2.4$	$Q=M_W$	MCFM+FEWZ
	$W+c$ total	[60]	5 (5/0)	$0 \le |\eta _l| \le 2.1$	$Q=M_W$	MCFM
	$W+c$ ratio	[60]	5 (5/0)	$0 \le |\eta _l| \le 2.1$	$Q=M_W$	MCFM
	2D DY 2011 7 TeV	[54]	124 (88/110)	$0 \le |\eta _{ll}| \le 2.2$	$20 \le M_{ll} \le 200$	MCFM+FEWZ
	[2D DY 2012 8 TeV]	[84]	124 (108/108)	$0 \le |\eta _{ll}| \le 2.4$	$20 \le M_{ll} \le 1200$	MCFM+FEWZ
	$W^{\pm }$ rap 8 TeV (*)	[79]	22 (22/22)	$0 \le |\eta _{l}| \le 2.3$	$Q=M_W$	MCFM+FEWZ
	$Z\, p_T$ 8 TeV (*)	[83]	50 (28/28)	$0.0 \le |y_{Z}| \le 1.6$	$30 \le p_T^Z \le 170$	MCFM+NNLO
	7 TeV jets 2011	[59]	133 (133/133)	$0 \le |y^\mathrm{jet}| \le 2.5$	$114 \le p_T^\mathrm{jet} \le 2116$	NLOjet++
	2.76 TeV jets (*)	[80]	81 (81/81)	$0 \le |y_{\mathrm{jet}}| \le 2.8$	$80 \le p_T^\mathrm{jet} \le$ 570	NLOjet++
	$\sigma _{\mathrm{tot}}(t\bar{t})$	[82, 88]	3 (3/3)	–	$Q=m_t$	top++
	$(1/\sigma _{t\bar{t}})d\sigma (t\bar{t})/y_{t\bar{t}}$ (*)	[81]	10 (10/10)	$-2.1<y_{t\bar{t}}<2.1$	$Q=m_t$	Sherpa+NNLO
LHCb	*Z* rapidity 940 pb	[55]	9 (9/9)	$2.0 \le \eta _l \le 4.5$	$Q=M_Z$	MCFM+FEWZ
	$Z\rightarrow ee$ rapidity 2 fb	[56]	17 (17/17)	$2.0 \le \eta _l \le 4.5$	$Q=M_Z$	MCFM+FEWZ
	$W,Z\rightarrow \mu$ 7 TeV (*)	[85]	33 (33/29)	$2.0 \le \eta _l \le 4.5$	$Q=M_W,M_Z$	MCFM+FEWZ
	$W,Z\rightarrow \mu$ 8 TeV (*)	[86]	34 (34/30)	$2.0 \le \eta _l \le 4.5$	$Q=M_W,M_Z$	MCFM+FEWZ

An overview of the data included in NNPDF3.1 is presented in Tables 1, 2, and 3, for the DIS structure function data, the fixed-target and Tevatron Drell–Yan experiments, and the LHC datasets, respectively. For each dataset we indicate the corresponding published reference, the number of data points in the NLO/NNLO PDF determinations before and after (in parentheses) kinematic cuts, the kinematic range covered in the relevant variables after cuts, and the code used to compute the NLO and NNLO results. Datasets included for the first time in NNPDF3.1 are flagged with an asterisk. The datasets not used for the default determination are in brackets. The total number of data points for the default PDF determination is 4175 / 4295 / 4285 at LO/NLO/NNLO.

**Fig. 1:**
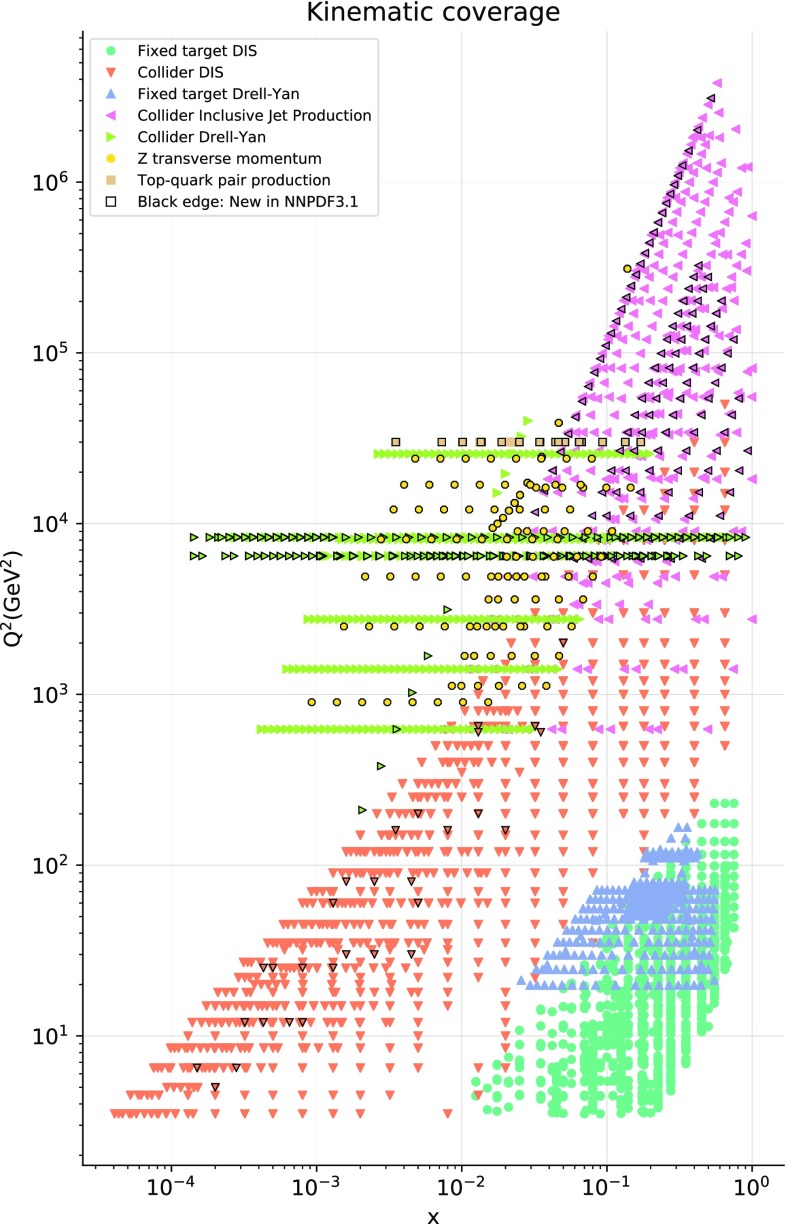
The kinematic coverage of the NNPDF3.1 dataset in the $\left( x,Q^2\right)$ plane

In Fig. 1 we show the kinematic coverage of the NNPDF3.1 dataset in the $\left( x,Q^2\right)$ plane. For hadronic data, leading-order kinematics have been assumed for illustrative purposes, with central rapidity used when rapidity is integrated over and the plotted value of $Q^2$ set equal to the factorization scale. It is clear that the new data added in NNPDF3.1 are distributed in a wide range of scales and *x*, considerably extending the kinematic reach and coverage of the dataset.

In Table 4 we present a summary of the kinematic cuts applied to the various processes included in NNPDF3.1 at NLO and NNLO. These cuts ensure that only data where theoretical calculations are reliable are included. Specifically, we always remove from the NLO dataset points for which the NNLO corrections exceed the statistical uncertainty. The further cuts collected in Table 4, specific to individual datasets, will be described when discussing each dataset in turn. All computations are performed up to NNLO in QCD, not including electroweak corrections. We have checked that with the cuts described in Table 4, electroweak corrections never exceed experimental uncertainties.

The codes used to perform NLO computations will be discussed in each subsection below. With the exception of deep-inelastic scattering, NNLO corrections are implemented by computing at the hadron level the bin-by-bin ratio of the NNLO to NLO prediction with a pre-defined PDF set, and applying the correction to the NLO computation (see Sect. 2.3 of Ref. [5]). For all new data included in NNPDF3.1, the PDF set used for the computation of these correction factors (often refereed to as *K*-factors, and in Ref. [5] as *C*-factors) is NNPDF3.0, except for the CMS W rap 8 TeV and ATLAS W/Z 2011 entries of Table 3 for which published xFitter results have been used and the CMS 2D DY 2012 data for which MMHT PDFs have been used [89] (see Sect. 2.5 below); the PDF dependence of the correction factors is much smaller than all other relevant uncertainties as we will demonstrate explicitly in Sect. 2.7 below.

**Table 4:** Full set of kinematical cuts applied to the processes used for NNPDF3.1 PDF determination at NLO and at NNLO. Only data satisfying the constraints in the table are retained. The experiments in brackets are not part of the global dataset and only used for dedicated studies. The cut on the HERA charm structure function data at NNLO is applied only when charm is fitted, and it is applied in addition to the other DIS kinematical cuts

Dataset	NLO	NNLO
DIS structure functions	$W^2\ge 12.5$ GeV$^2$	$W^2\ge 12.5$ GeV$^2$
	$Q^2\ge 3.5$ GeV$^2$	$Q^2\ge 3.5$ GeV$^2$
HERA $\sigma _c^\mathrm{NC}$ (in addition)	–	$Q^2 \ge 8$ GeV$^2$ (fitted charm)
ATLAS 7 TeV inclusive jets 2011	$|y_{\mathrm{jet}}|\le 0.4$	$|y_{\mathrm{jet}}|\le 0.4$
Drell–Yan E605 and E866	$\tau \le 0.080$	$\tau \le 0.080$
	$|y/y_{\mathrm{max}}|\le 0.663$	$|y/y_{\mathrm{max}}|\le 0.663$
D0 $W \rightarrow l\nu$ asymmetries	–	$|A_{l}|\ge 0.03$
CMS Drell–Yan 2D 7 TeV	30 GeV $\le M_{ll}\le 200$ GeV	$M_{ll}\le 200$ GeV
	$|y_Z|\le 2.2$	$|y_Z|\le 2.2$
[CMS Drell–Yan 2D 8 TeV]	$M_{ll}\ge 30$ GeV	$M_{ll}\ge 30$ GeV
LHCb 7 TeV and 8 TeV $W,Z\rightarrow \mu$	–	$|y_{l}|\ge 2.25$
[ATLAS $Z\, p_T$ 7 TeV]	$30~\mathrm{GeV}\le p_T^Z \le 500~\mathrm{GeV}$	$30~\mathrm{GeV}\le p_T^Z \le 500~\mathrm{GeV}$
ATLAS $Z\, p_T$ 8 TeV $(p_T,M_{ll})$	$p_T^Z\ge 30~\mathrm{GeV}$	$p_T^Z\ge 30~\mathrm{GeV}$
ATLAS $Z\, p_T$ 8 TeV $(p_T,y_{Z})$	$30~\mathrm{GeV}\le p_T^Z \le 150~\mathrm{GeV}$	$30~\mathrm{GeV}\le p_T^Z \le 150~\mathrm{GeV}$
CMS $Z\, p_T$ 8 TeV $(p_T,y_{Z})$	$30~\mathrm{GeV}\le p_T^Z \le 170~\mathrm{GeV}$	$30~\mathrm{GeV}\le p_T^Z \le 170~\mathrm{GeV}$
	$|y_Z| \le 1.6$	$|y_Z| \le 1.6$

### Deep-inelastic structure functions

The main difference between the NNPDF 3.0 and 3.1 DIS structure function datasets is the replacement of the separate HERA-I and ZEUS/H1 HERA-II inclusive structure function measurements by the final legacy HERA combination [9]. The impact of the HERA-II data on a global fit which includes HERA-I data is known [5, 90–92] to be moderate to begin with; the further impact of replacing the separate HERA-I and HERA-II data used in NNPDF3.0 with their combination has been studied in [93] and found to be completely negligible.

Additionally, the NNPDF3.1 dataset includes the H1 and ZEUS measurements of the bottom structure function $F_2^b(x,Q^2)$ [67, 68]. While the $F_2^b$ dataset is known to have a very limited pull, the inclusion of this dataset is useful for applications, such as the determination of the bottom mass [94].

While it is not included in the default NNPDF3.1 dataset, the EMC data on charm structure functions [69] will also be used for specific studies of the charm content of the proton in Sect. 5.3. As discussed in Refs. [23, 95], the EMC dataset has been corrected by updating the BR$(D\rightarrow \mu )$ branching ratio: the value used in the original analysis [69] is replaced with the latest PDG value [96]. A conservative uncertainty on this branching ratio of $\pm 15\%$ is also included.

The cuts applied to DIS data are as follows. As in NNPDF3.0, for all structure function datasets we exclude data with $Q^2 < 3.5$ GeV$^2$ and $W^2< 12.5$ GeV$^2$, i.e. the region where higher twist corrections might become relevant and the perturbative expansion may become unreliable. At NNLO we also remove $F_2^c$ data with $Q^2 < 8$ GeV$^2$ in order to minimize the possible impact of unknown NNLO terms related to initial-state charm (see below).

The computation of structure functions has changed in comparison to previous NNPDF releases. Indeed, in NNPDF3.0 the solution of the DGLAP evolution equations and the structure functions were computed with the internal NNPDF code FKgenerator [97, 98], based on the Mellin-space formalism. In NNPDF3.1, as was already the case in the charm study of Ref. [23], PDF evolution and DIS structure functions are computed using the APFEL public code [99], based instead on the *x*-space formalism. The two codes have been extensively benchmarked against each other; see Appendix A. DIS structure functions are computed at NLO in the FONLL-B general-mass variable-flavor number scheme, and at NNLO in the FONLL-C scheme [100]. All computations include target-mass corrections.

In NNPDF3.1 we now parametrize charm independently, and thus the FONLL GM-VFN has been extended in order to include initial-state heavy quarks. This is accomplished using the formalism of Refs. [21, 22]. Within this formalism, a massive correction to the charm-initiated contribution is included alongside the contribution of fitted charm as a non-vanishing boundary condition to PDF evolution. At NNLO this correction requires knowledge of massive charm-initiated contributions to the DIS coefficient functions up to $\mathcal {O}\left( \alpha _S^2\right)$, which are currently only known to $\mathcal {O}\left( \alpha _S\right)$ [101]. Therefore, in the NNLO PDF determination, the NLO expression for this correction is used: this corresponds to setting the unknown $\mathcal {O}\left( \alpha ^2_S\right)$ contribution to the massive charm-initiated term to zero. Such an approximation was used in Ref. [23], where it was shown that it is justified by the fact that even setting to zero the full correction (i.e. using the LO expression for the massive correction) has an effect which at the PDF level is much smaller than PDF uncertainties (see in particular Fig. 10 of Ref. [23]).

**Fig. 2:**
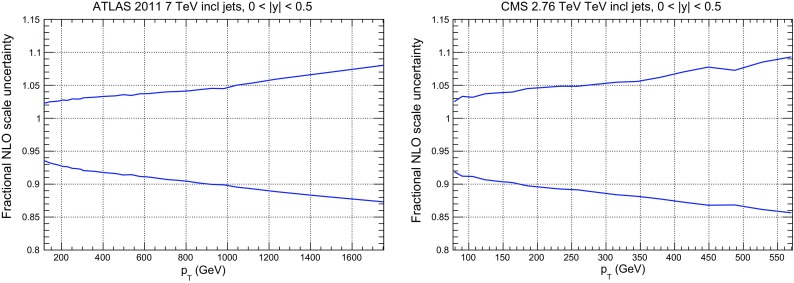
The fractional scale uncertainty on NLO single-inclusive jet production, as a function of the jet $p_T$ for the central rapidity bins of ATLAS 7 TeV 2011 (left) and the CMS 2.76 TeV (right)

Finally, as in previous NNPDF studies, no nuclear corrections are applied to the deuteron structure function and neutrino charged-current cross-section data taken on heavy nuclei, in particular NuTeV and CHORUS. We will return to this issue in Sect. 4.11.

### Fixed-target Drell–Yan production

In NNPDF3.1 we have included the same fixed-target Drell–Yan (DY) data as in NNPDF3.0, namely the Fermilab E605 and E866 datasets; in the latter case both the proton–proton data and the ratio of cross-sections between deuteron and proton targets, $\sigma _\mathrm{DY}^d/\sigma _{\mathrm{DY}}^p$ are included. However, the kinematic cuts applied to these two experiments differ from those in NNPDF3.0, based on the study of [102], which showed that theoretical predictions for data points too close to the production threshold become unstable. Requiring reliability of the fixed-order perturbative approximation leads to the cuts

2.1 $$\begin{aligned} \tau \le 0.08 \, \quad \mathrm{and} \quad |y/y_{\mathrm{max}}|\le 0.663 \, , \end{aligned}$$

where $\tau =M_{ll}^2/s$ and $y_{\mathrm{max}}=-{{1}\over {2}} \ln \tau$, with $M_{ll}$ the dilepton invariant mass distribution and $\sqrt{s}$ the center-of-mass energy of the collision.

As in the case of DIS, NLO fixed-target Drell–Yan cross-sections were computed in NNPDF3.0 using the Mellin-space FKgenerator code, while in NNPDF3.1 they are obtained using APFEL. The two computations are benchmarked in Appendix A. NNLO corrections are determined using Vrap [103]. Once more, as in previous NNPDF studies, no nuclear corrections are applied; again we will return to this issue in Sect. 4.11 below.

### Single-inclusive jets

Four single-inclusive jet cross-section measurements were part of the NNPDF3.0 dataset: CDF Run II $k_T$ [44], CMS 2011 [59], ATLAS 7 TeV 2010 and ATLAS 2.76 TeV, including correlations to the 7 TeV data [57, 58]. On top of these, in NNPDF3.1 we also include the ATLAS 7 TeV 2011 [76] and CMS 2.76 TeV [80] data. Some of these measurements are available for different values of the jet *R* parameter; the values used in NNPDF3.1 are listed in Table 5.

**Table 5:** Values of the jet *R* parameter used for the jet production datasets included in NNPDF3.1

Dataset	Ref.	Jet radius
CDF Run II $k_t$ incl jets	[87]	$R=0.7$
ATLAS 7 TeV jets 2010	[57]	$R=0.4$
ATLAS 2.76 TeV jets	[58]	$R=0.4$
ATLAS 7 TeV jets 2011	[76]	$R=0.6$
CMS 7 TeV jets 2011	[59]	$R=0.7$
CMS 2.76 TeV jets	[80]	$R=0.7$

No cuts are applied to any of the jet datasets included in NNPDF3.1, except for the ATLAS 2011 7 TeV data, for which achieving a good description turns out to be impossible if all five rapidity bins are included simultaneously. We can obtain good agreement between data and theory when using only the central rapidity bin, $|\eta ^\mathrm{jet}|<0.4$. The origin of this state of affairs is not understood: we have verified that a reasonable description can be obtained if some of the systematic uncertainties are decorrelated, but we have no justification for such a procedure. We have therefore chosen to only include in NNPDF3.1 data from the central rapidity bin, $|\eta ^\mathrm{jet}|<0.4$ for this set. This is also the rapidity bin with the largest PDF sensitivity [104].

In NNPDF3.1, all NLO jet cross-sections are computed using NLOjet++ [105] interfaced to APPLgrid [106]. The jet $p_T$ is used as the central factorization and renormalization scale in all cases, as this choice exhibits improved perturbative convergence compared with other scale choices such as the leading jet $p_T^{1}$ [107, 108].

While the NNLO calculation of inclusive jet production has been recently published [20, 108], results are not yet available for all datasets included in NNPDF3.1. Therefore, jet data are included as default in the NNPDF3.1 NNLO determination using NNLO PDF evolution but NLO matrix elements, while adding to the covariance matrix an additional fully correlated theoretical systematic uncertainty estimated from scale variation of the NLO calculation. The NLO scale variations are performed using APPLgrid interfaced to HOPPET [109]. We take the associated uncertainty as the envelope of the result of seven-point scale variation $\mu _\mathrm{F} \in \left[ p_T/2,2p_T\right]$ and $\mu _\mathrm{R} \in \left[ p_T/2,2p_T\right]$ with $1/2 \le \mu _\mathrm{F}/\mu _\mathrm{R} \le 2$. The NNLO corrections are generally well within this scale variation band when the jet $p_T$ is chosen as a central scale [108]. This scale uncertainty is shown in Fig. 2 for ATLAS 7 TeV 2011 and CMS 2.76 TeV as a function of the jet $p_T$ for the central rapidity bin. It is seen to range between a few percent at low $p_T$ up to around 10% at the largest $p_T$. A similar behavior is observed in other rapidity bins, with a more asymmetric band at forward rapidity.

**Fig. 3:**
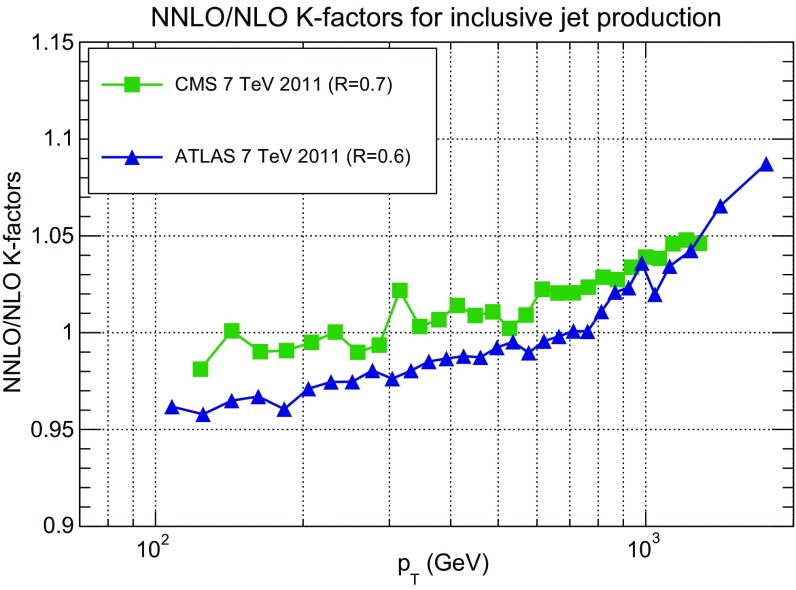
The NNLO/NLO cross-section ratio [110] for the central rapidity bin ($0\le |y_{\mathrm{jet}}|\le 0.5$) of the ATLAS and CMS 7 TeV 2011 jet data, with the values of *R* of Table 5, plotted vs. $p_T$

In order to gauge the reliability of our approximate treatment of the jet data, we have produced a PDF determination in which all data for which NNLO corrections are known, namely the 7 TeV ATLAS and CMS datasets, are included using exact NNLO theory. This will be discussed in Sect. 4.4. Representative NNLO corrections are shown in Fig. 3, where we show the NNLO/NLO ratio for the central rapidity bin ($0\le |y_{\mathrm{jet}}|\le 0.5$) of the ATLAS and CMS 7 TeV 2011 datasets, plotted as a function of $p_T$ [110]: note (see Table 5) that the values of *R* are different, thereby explaining the different size of the correction, which for CMS is ${\sim }-2\%$ for $p_T\sim 100$ GeV, increasing up to ${\sim }5\%$ for $p_T\sim 2$ TeV, and for ATLAS it ranges from ${\sim }-4\%$ increasing up to ${\sim }9\%$ as a function of $p_T$. Unlike in the case of the *Z* transverse momentum distribution, to be discussed in Sect. 2.6, the lack of smoothness of the corrections seen in Fig. 3 is not problematic as the fluctuations are rather smaller than typical uncorrelated uncertainties on these data.

### Drell–Yan production at hadron colliders

The NNPDF3.0 determination already included a wide set of collider Drell–Yan data, both at the *W* and *Z* peak and off-shell. This dataset has been further expanded in NNPDF3.1. We discuss here invariant mass and rapidity distributions; transverse momentum distributions will be discussed in Sect. 2.6.

In NNPDF3.1 we include for the first time D0 legacy *W* asymmetry measurements based on the complete dataset in the electron [14] and muon [13] channels. The only cut applied to this dataset is at NNLO, where we remove data with $\mathcal {A}_l(y_l)\le 0.03$ in both the electron and the muon channel data. This is due to the fact that when the asymmetry is very close to zero, even with high absolute accuracy on the NNLO theoretical calculation, it is difficult to achieve high percentage accuracy, thereby making the NNLO correction to the asymmetry unreliable. The NLO computation is performed using APPLgrids from the HERAfitter study of [70], which we have cross-checked using Sherpa [111] interfaced to MCgrid [112]. NNLO corrections are computed using FEWZ [113–115].

**Fig. 4:**
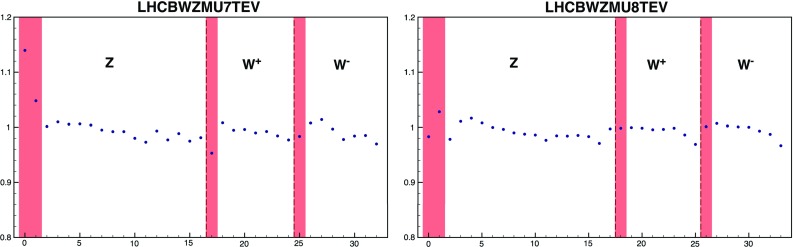
The NNLO/NLO cross-section for the LHCb 7 (left) and 8 TeV (right) data. The central rapidity region which is cut is shaded in red

New results are included for ATLAS, CMS and LHCb. For ATLAS, NNPDF3.0 included 2010 *W* and *Z* 7 TeV rapidity distributions and their cross-correlations [49]. A recent update of the same measurement [72], based on the entire 7 TeV integrated luminosity of 4.6 fb$^{-1}$ is included in NNPDF3.1, albeit partially. This measurement provides differential distributions in lepton pseudo-rapidity $|\eta _l|$ in the range $0\le |\eta _l|\le 2.5$ for on-shell $W^+$ and $W^-$ production. For $Z/\gamma ^*$ production results are provided either with both leptons measured in the range $0\le |\eta _l|\le 2.5$, or with one lepton with $0\le |\eta _l|\le 2.5$ and the other with $2.5\le |\eta _l|\le 4.9$. The central rapidity data are given for three bins in the dilepton invariant mass $46<m_{ll}<66$, $66<m_{ll}<116$ and $116<m_{ll}<150$ GeV, and the forward rapidity data in the last two mass bins (on-peak and high-mass). We only include the on-shell, $0\le |\eta _l|\le 2.5$ data, thereby neglecting the two low- and high-mass *Z* production bins in the central rapidity region, and the on-peak and high-mass *Z* production bins at forward rapidity. The full dataset will be included in future NNPDF releases. No other cuts are applied to the dataset. Theoretical predictions are obtained using NLO APPLgrids [106] generated using MCFM [116], while the NNLO corrections are taken from the xFitter analysis of Ref. [72].

Also new to NNPDF3.1 is the ATLAS low-mass Drell–Yan data from Ref. [77]. We use only the low-mass DY cross-sections in the muon channel measured from $35~{\mathrm {pb}}^{-1}$ 2010 dataset, which extends down to $M_{ll}=12$ GeV. The 2011 7 TeV data with invariant masses between 26 GeV and 66 GeV are not included because they are affected by large electroweak corrections and are therefore excluded by our cuts. Furthermore, two datapoints are removed from the NLO datasets because NNLO corrections exceed experimental uncertainties. Theoretical predictions are obtained at NLO using APPLgrids [106] constructed using MCFM, and at NNLO corrections are computed using FEWZ.

For CMS, NNPDF3.1 includes 8 TeV $W^+$ and $W^-$ rapidity distributions, including information on their correlation [79]. No cuts have been applied to this dataset. Theoretical predictions are obtained using the NLO APPLgrids generated with MCFM and the NNLO correction factors are computed using FEWZ in the context of the xFitter [117] analysis presented in Ref. [79]. Double-differential rapidity $y_{ll}$ and invariant mass $M_{ll}$ distributions for $Z/\gamma ^*$ production from the 2012 8 TeV data [84] have been studied by including them in a specialized PDF determination. However, the dataset has been left out of default NNPDF3.1 dataset, for reasons to be discussed in Sect. 4.8. The only cut applied to this dataset, based on a previous MMHT analysis [89] is $M_{\ell \ell }\ge 30$ GeV, because in the lowest-mass bin the leading-order prediction vanishes. Theoretical predictions are obtained at NLO using APPLgrids constructed using MCFM, and at NNLO corrections have been computed [89] using FEWZ.

For LHCb, previous data included in NNPDF3.0 are replaced by the final 7 TeV and 8 TeV $W^+$, $W^-$ and *Z* rapidity distributions in the muon channel [85, 86]. The NNLO/NLO cross-section ratios are shown in Fig. 4. The date with $|y_l|\le 2.25$ from this set have been cut because the anomalously large size of the NNLO corrections suggests that they may be unreliable. Theoretical predictions are obtained at NLO using APPLgrids constructed using MCFM, and at NNLO corrections computed using FEWZ.

### The transverse momentum of *Z* bosons

The transverse momentum distribution of the *Z* boson is included for the first time in a global PDF determination thanks to the recent computation of the process at NNLO [18, 118–120]. In the NNPDF3.1 determination we include recent datasets from ATLAS and CMS following the detailed study in Ref. [121].

ATLAS has published measurements of the spectrum of the *Z* transverse momentum at 7 TeV [78] and at 8 TeV [71]. Measurements are performed in the $Z/\gamma ^*\rightarrow e^+e^-$ and $Z/\gamma ^*\rightarrow \mu ^+\mu ^-$ channels which are then combined. The 7 TeV data are based on an integrated luminosity of 4.7 $\mathrm{fb}^{-1}$, while the 8 TeV data are based on an integrated luminosity of 20.3 fb$^{-1}$. We now discuss each of these two datasets in turn.

The 7 TeV data are taken at the *Z* peak, reaching values of the *Z* transverse momentum of up to $p_T^Z=800$ GeV. They are given inclusively for $Z/\gamma ^*$ rapidities up to $|y_Z|=2.4$, as well as in three separated rapidity bins given by $0.0\le |y_Z| \le 1.0$, $1.0 \le |y_Z| \le 2.0$ and $2.0 \le |y_Z| \le 2.4$. In order to maximize the potential constraint on PDFs, only the differential measurement will be considered. The measurement is presented in terms of normalized cross-sections $(1/\sigma _Z)\,\mathrm{d}\sigma (Z)/\mathrm{d} p_T^Z$, where $\sigma _Z$ is the fiducial cross-section in the corresponding di-lepton rapidity bin. This dataset has been left out of the default NNPDF3.1 dataset, for reasons to be discussed in Sect. 4.2.

The 8 TeV dataset, which reaches $p_T^Z$ values as high as 900 GeV, is presented in three separate invariant mass bins: low mass below the *Z*-peak, on-peak, and high mass above the *Z*-peak up to $M_{ll}=$ 150 GeV. In addition, the measurement taken at the *Z*-peak is provided both inclusively in the whole rapidity range $0.0<|y_Z|<2.4$ as well as exclusively in six separate rapidity bins $0<y_Z<0.4$, $0.4<|y_Z|<0.8$, $0.8<|y_Z|<1.2$, $1.2<|y_Z|<1.6$, $1.6<|y_Z|<2.0$ and $2.0<|y_Z|<2.4$. Once again, here the more differential measurement will be used. In contrast to the 7 TeV data, the dataset is given both in terms of normalized and absolute distributions. We will use the latter, not only because of the extra information on the cross-section normalization, but also as problems can occur whenever the data used to compute the normalization are provided in a range which differs from that of the data used for PDF determination. This problem is discussed in detail in Ref. [121] and described in Sect. 4.2.

**Fig. 5:**
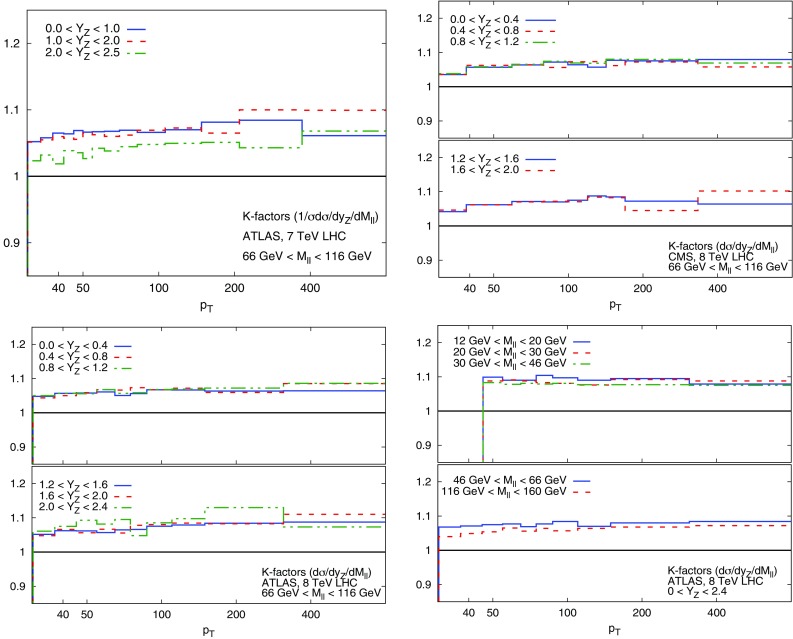
The NNLO/NLO cross-section for the $Z\,p_T$ data corresponding to the acceptance cuts and binning of the ATLAS 7 TeV (top left), CMS 8 TeV (top right), and the ATLAS 8 TeV (bottom) rapidity (left) and invariant mass (right) distributions

CMS has measured the cross-sections differentially in $p_T$ and rapidity $y_Z$ at 8 TeV [83], based on an integrated luminosity of 19.7 fb$^{-1}$ in the muon channel. Data is provided in five rapidity bins $0.0<|y_Z|<0.4$, $0.4<|y_Z|<0.8$, $0.8<|y_Z|<1.2$, $1.2<|y_Z|<1.6$ and $1.6<|y_Z|<2.0$. We do not consider a previous CMS measurement at 7 TeV [122], which is based on a smaller dataset, and would constitute double counting of the double-differential distributions [54] already included in NNPDF3.0, and retained in NNPDF3.1.

Three sets of kinematic cuts are applied to the data. Firstly, ensuring the reliability of fixed-order perturbation theory imposes a cut of $p_T^Z \ge$ 30 GeV (resummation would be required for smaller $p_T$) [121]. Secondly, removing regions in which electroweak corrections are large and comparable to the experimental data imposes a cut of $p_T^Z \le 150~(170)$ GeV for the ATLAS (CMS) data [121]. Finally, the CMS dataset in the largest rapidity bin is discarded due to an apparent incompatibility with both the corresponding ATLAS measurement in the same bin and the theoretical prediction. The origin of this incompatibility remains unclear [121].

Theoretical predictions have been obtained from Ref. [121], based upon the NNLO computation of *Z*+jet production of Refs. [119, 120]. Factorization and renormalization scales are chosen as

2.2 $$\begin{aligned} \mu _\mathrm{R}=\mu _\mathrm{F} = \sqrt{(p_T)^2+M_{ll}^2} \, , \end{aligned}$$

where $M_{ll}$ is the invariant mass of the final-state lepton pair. The calculation includes the *Z* and $\gamma ^*$ contributions, their interference and decay to lepton pairs. The NNLO/NLO ratio is shown in Fig. 5 for the observables with the ATLAS and CMS acceptance cuts, computed using NNPDF3.0 PDFs, with $\alpha _s(m_Z)=0.118$; the NNLO correction varies from around 2–3% at low $p_T$ up to around 10% at high $p_T$ and is therefore required in order to describe data with sub-percent accuracy.

**Fig. 6:**
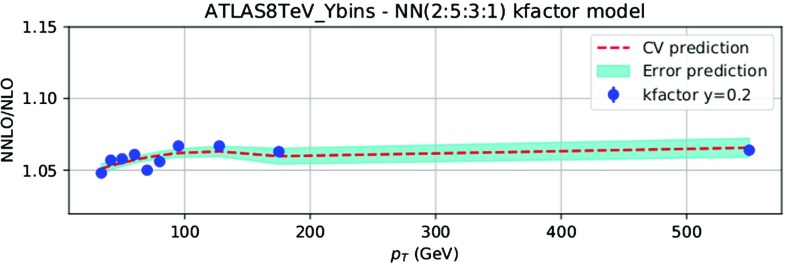
The NNLO/NLO cross-section ratio in the central rapidity bin of the 8 TeV ATLAS $Z\, p_T$ distribution. The result of a fit and its associate uncertainty are also shown

Even the most accurate results for the NNLO/NLO correction factor still display fluctuations, as shown in Fig. 5 where we plot the NNLO/NLO cross-section ratio for the central rapidity bin of the 8 TeV ATLAS data. The points are shown together with their nominal Monte Carlo integration uncertainty [121]. The point-to-point statistical fluctuation of the theoretical prediction appears to be larger than the typical uncorrelated statistical uncertainty on the ATLAS dataset, which is typically at the sub-percent or even permille level. In order to check this, we have fitted an ensemble of neural networks to the cross-section ratio, as a function of $p_T^Z$ for fixed rapidity. The fit has been performed in each of the rapidity bins for the ATLAS and CMS data; more details are given in Ref. [123]. The result of the fit and its one-sigma uncertainty are shown in Fig. 6 for the central rapidity bin of the ATLAS data.

The one-sigma uncertainty of the fit, which is determined by the point-to-point fluctuation of the NNLO computation, is at the percent level, which is rather larger than the statistical uncertainty of the data. Indeed, it is clear by inspection of Figs. 5 and 6 that the point-to-point fluctuations of the NNLO/NLO ratio are much larger than those of the data themselves (as seen in Refs. [78, 121]). We conclude that there is a residual theoretical uncertainty on the NNLO prediction which we estimate to be of order of 1% for all datasets. This conclusion has been validated and cross-checked by repeating the fit with cuts or different functional forms. We have therefore added an extra 1% fully uncorrelated theoretical uncertainty to this dataset (see also Ref. [121]).

### Differential distributions and total cross-sections in $t\bar{t}$ production

Differential distributions for top-pair production have been included in NNPDF3.1 following the detailed study of Ref. [124]. ATLAS and CMS have performed measurements of these distributions with a variety of choices of kinematic variables, including the top-quark rapidity $y_t$, the rapidity of the top pair $y_{t\bar{t}}$, the transverse momentum of the top-quark $p_T^t$, and the invariant mass of the top–anti-top system $m_{t\bar{t}}$. For ATLAS both absolute and normalized differential distributions are provided, whereas CMS only provides normalized results. Perturbative QCD corrections for all these distributions have been computed at NNLO [15, 16]. In order to avoid double counting, only one distribution per experiment can be included in the dataset, as the statistical correlations between different distributions are not available. The choice of differential distributions adopted in NNPDF3.1 follows the recommendation of Ref. [124], where a comprehensive study of the impact on the gluon PDF of various combinations of differential top-pair distributions was performed. It was found that the normalized rapidity distributions have the largest constraining power and lead to good agreement between theory and data for ATLAS and CMS. The use of rapidity distributions has some further advantages. First, it reduces the risk of possible contamination by BSM effects. For example, heavy resonances would be kinematically suppressed in the rapidity distributions, but not in the tails of the $m_{t\bar{t}}$ and $p_T^t$ distributions. Second, rapidity distributions exhibit a milder sensitivity upon variations of the value of $m_t$ than the $p_T^t$ and $m_{t\bar{t}}$ distributions [125].

We therefore include the 8 TeV normalized rapidity distributions in the lepton+jets final state from ATLAS [73] and CMS [81], which correspond, respectively, to an integrated luminosity of 20.3 and 19.7 fb$^{-1}$. We consider measurements in the full phase space, with observables reconstructed in terms of the top or top-pair kinematic variables, because NNLO results are available only for stable top quarks. We also include, again following Ref. [124], the most recent total cross-sections measurements at 7, 8 and 13 TeV from ATLAS [74, 75] and CMS [82, 88]. They replace previous measurements from ATLAS [61–63] and CMS [64–66] included in NNPDF3.0.

**Fig. 7:**
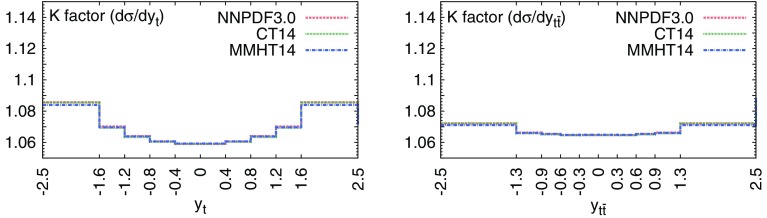
The NNLO/NLO cross-section ratio for the top-quark rapidity $y_t$ (left) and top-quark pair rapidity $y_{t\bar{t}}$ (right) corresponding to the 8 TeV ATLAS and CMS data. Results obtained with three different input PDF sets, NNPDF3.0, CT14, and MMHT14, are shown

At NLO theoretical predictions have been generated with Sherpa [111], in a format compliant to APPLgrid [106], using the MCgrid code [112] and the Rivet [126] analysis package, with OpenLoops [127] for the one-loop matrix elements. All calculations have been performed with large Monte Carlo integration statistics in order to ensure that residual numerical fluctuations are negligible. Our results have been carefully benchmarked against those obtained from the code of [16]. Renormalization and factorization scales, $\mu _\mathrm{R}$ and $\mu _\mathrm{F}$ respectively, have been chosen based on the recommendation of Ref. [16] as

2.3 $$\begin{aligned}&\mu _\mathrm{R}=\mu _\mathrm{F}=\mu =H_T/4 \, , \qquad H_T \equiv \sqrt{m_t^2+\left( {p_T^t}\right) ^2} \nonumber \\&\quad \qquad +\, \sqrt{m_t^2+\left( {p_T^{\bar{t}}}\right) ^2} \, , \end{aligned}$$

where $m_t=173.3$ GeV is the PDG world average for the top-quark pole mass [128], and $p_T^t$ ($p_T^{\bar{t}}$) is the top (anti-top) transverse momentum. NLO theoretical predictions for normalized differential distributions have been obtained by dividing their absolute counterparts by the cross-section integrated over the kinematic range of the data.

The NNLO correction factors have been computed separately for the absolute differential cross-sections and their normalizing total cross-sections. Differential cross-sections have been determined using the code of [16], with the scale choice Eq. (2.3). Results for the NNLO/NLO ratio are shown in Fig. 7, where it can be seen that the size of the NNLO corrections is between 6% and $9\%$, actually smaller than the data uncertainty, with a reasonably flat shape in the kinematic region covered by the data. We also show explicitly the dependence of the results on the PDF set used in the calculation by using three different global PDF sets: it is clear that this dependence is completely negligible.

Total cross-sections have been computed with the top++ code [129] at NNLO+NNLL, and with fixed scales $\mu _\mathrm{R}=\mu _\mathrm{F}=m_t$, following the recommendation of Ref. [16] which suggests that NNLO+NNLL resummed cross-sections should be used in conjunction to NNLO differential distributions if the latter are determined using a dynamical scale choice.

## The NNPDF3.1 global analysis

We now present the results of the NNPDF3.1 global analysis at LO, NLO and NNLO, and compare them with the previous release NNPDF3.0 and with other recent PDF sets. Here we present results obtained using the complete dataset of Tables 1, 2, 3, discussed in Sect. 2. Studies of the impact of individual measurements will be discussed along with PDF determinations from reduced datasets in Sect. 4.

After a brief methodological summary, we discuss the fit quality, and then examine individual PDFs and their uncertainties. We compare NNPDF3.1 PDFs with NNPDF3.0 and with CT14 [6], MMHT2014 [7] and ABMP16 [8]. We next examine the impact of independently parametrizing charm, the principal methodological improvement in NNPDF3.1. Finally, we discuss theoretical uncertainties, both related to QCD parameters and to missing higher-order corrections to the theory used for PDF determination.

In this section all NLO and NNLO NNPDF3.1 results are produced using the CMC [25] optimized 100 replica Monte Carlo sets, see Sect. 6.2 below: despite only including 100 replicas, these sets reproduce the statistical features of a set of at least about 400 replicas (see Sect. 6.1). We present here only a selection of results: a more extensive set of results is available from a public repository; see Sect. 6.2.

### Methodology

NNPDF3.1 PDFs are determined with largely the same methodology as in NNPDF3.0: the only significant change is that now charm is independently parametrized. The PDF parametrization is identical to that discussed in Sect. 3.2 of Ref. [5], including the treatment of preprocessing, but with the PDF basis in Eq. (3.4) of that reference now supplemented by an extra PDF for charm, parametrized like all other PDFs (as per Eq. (2) of Ref. [23]). PDFs are parametrized at the scale $Q_0=1.65$ GeV whenever the charm PDF is independently parametrized. For the purposes of comparison we also provide PDF sets constructed with perturbatively generated charm; in these sets, PDFs are parametrized at the scale $Q_0=1.0$ GeV. This ensures that the parametrization scale is always above the charm mass when charm is independently parametrized, and below it when it is perturbatively generated.

As in Ref. [5] we use $\alpha _s(m_Z)=0.118$ as a default throughout the paper, though determinations have also been performed for several different values of $\alpha _s$ (see Sect. 6.2). Heavy-quark pole masses are used throughout, with the main motivation that for the inclusive observables used for PDF determination $\overline{\mathrm{MS}}$ masses are inappropriate, since they distort the perturbative expansion in the threshold region [130]. The default values of the heavy-quark pole masses are $m_\mathrm{c}=1.51$ GeV for charm and $m_\mathrm{b}=4.92$ GeV for bottom, following the recommendation of the Higgs cross-section working group [131]; PDF sets for different charm mass values, corresponding to the ±one-sigma uncertainty band from Ref. [131], are also provided; see Sect. 6.2.

### Fit quality

**Table 6:** The values of $\chi ^2/N_{\mathrm{dat}}$ for the global fit and for all the datasets included in the NNPDF3.1 LO, NLO and NNLO PDF determinations. Values obtained using the NNPDF3.0 NLO and NNLO PDFs are also shown: numbers in brackets correspond to data not fitted in NNPDF3.0. Note that NNPDF3.0 values are produced using NNPDF3.1 theory settings, and they are thus somewhat worse than those quoted in Ref. [5]

Dataset	NNPDF3.1			NNPDF3.0	
	NNLO	NLO	LO	NNLO	NLO
NMC	1.30	1.35	3.25	1.29	1.36
SLAC	0.75	1.17	3.35	0.66	1.08
BCDMS	1.21	1.17	2.20	1.31	1.21
CHORUS	1.11	1.06	1.16	1.11	1.14
NuTeV dimuon	0.82	0.87	4.75	0.69	0.61
HERA I+II inclusive	1.16	1.14	1.77	1.25	1.20
HERA $\sigma _c^\mathrm{NC}$	1.45	1.15 ()	1.21	[1.61]	[2.57]
HERA $F_2^b$	1.11	1.08	11.2	[1.13]	[1.12]
DY E866 $\sigma ^d_{\mathrm{DY}}/\sigma ^p_{\mathrm{DY}}$	0.41	0.40	1.06	0.47	0.53
DY E886 $\sigma ^p$	1.43	1.05	0.81	1.69	1.17
DY E605 $\sigma ^p$	1.21	0.97	0.66	1.09	0.87
CDF *Z* rap	1.48	1.619	1.54	1.55	1.28
CDF Run II $k_t$ jets	0.87	0.84	1.07	0.82	0.95
D0 *Z* rap	0.60	0.67	0.65	0.61	0.59
D0 $W\rightarrow e\nu$ asy	2.70	1.59	1.75	[2.68]	[4.58]
D0 $W\rightarrow \mu \nu$ asy	1.56	1.52	2.16	[2.02]	[1.43]
ATLAS total	**1.09**	**1.36**	**5.34**	**1.92**	**1.98**
ATLAS *W*, *Z* 7 TeV 2010	0.96	1.04	2.38	1.42	1.39
ATLAS high-mass DY 7 TeV	1.54	1.88	4.05	1.60	2.17
ATLAS low-mass DY 2011	0.90	0.69	2.86	[0.94]	[0.81]
ATLAS *W*, *Z* 7 TeV 2011	2.14	3.70	27.2	[8.44]	[7.6]
ATLAS jets 2010 7 TeV	0.94	0.92	1.22	1.12	1.07
ATLAS jets 2.76 TeV	1.03	1.03	1.50	1.31	1.32
ATLAS jets 2011 7 TeV	1.07	1.12	1.59	[1.03]	[1.12]
ATLAS $Z\,p_T$ 8 TeV $(p_T^{ll},M_{ll})$	0.93	1.17	–	[1.05]	[1.28]
ATLAS $Z\;p_T$ 8 TeV $(p_T^{ll},y_{ll})$	0.94	1.77	–	[1.19]	[2.49]
ATLAS $\sigma _{tt}^\mathrm{tot}$	0.86	1.92	53.2	0.67	1.07
ATLAS $t\bar{t}$ rap	1.45	1.31	1.99	[3.32]	[1.50]
CMS total	**1.06**	**1.20**	**2.13**	**1.19**	**1.33**
CMS *W* asy 840 pb	0.78	0.86	1.55	0.73	0.85
CMS *W* asy 4.7 fb	1.75	1.77	3.16	1.75	1.82
CMS $W+c$ tot	–	0.54	16.5	–	0.93
CMS $W+c$ ratio	–	1.91	3.21	–	2.09
CMS Drell–Yan 2D 2011	1.27	1.23	2.15	1.20	1.19
CMS *W* rap 8 TeV	1.01	0.70	4.32	[1.24]	[0.96]
CMS jets 7 TeV 2011	0.84	0.84	0.93	1.06	0.98
CMS jets 2.76 TeV	1.03	1.01	1.09	[1.22]	[1.18]
CMS $Z\,p_T$ 8 TeV $(p_T^{ll},M_{ll})$	1.32	3.65	–	[1.59]	[3.86]
CMS $\sigma _{tt}^\mathrm{tot}$	0.20	0.59	53.4	0.56	0.10
CMS $t\bar{t}$ rap	0.94	0.96	1.32	[1.15]	[1.01]
LHCb total	**1.47**	**1.62**	**5.16**	**2.11**	**2.67**
LHCb *Z* 940 pb	1.49	1.27	2.51	1.29	0.91
LHCb $Z\rightarrow ee$ 2 fb	1.14	1.33	6.34	1.21	2.31
LHCb $W,Z \rightarrow \mu$ 7 TeV	1.76	1.60	4.70	[2.59]	[2.36]
LHCb $W,Z \rightarrow \mu$ 8 TeV	1.37	1.88	7.41	[2.40]	[3.74]
Total dataset	1.148	1.168	2.238	1.284	1.307

**Fig. 8:**
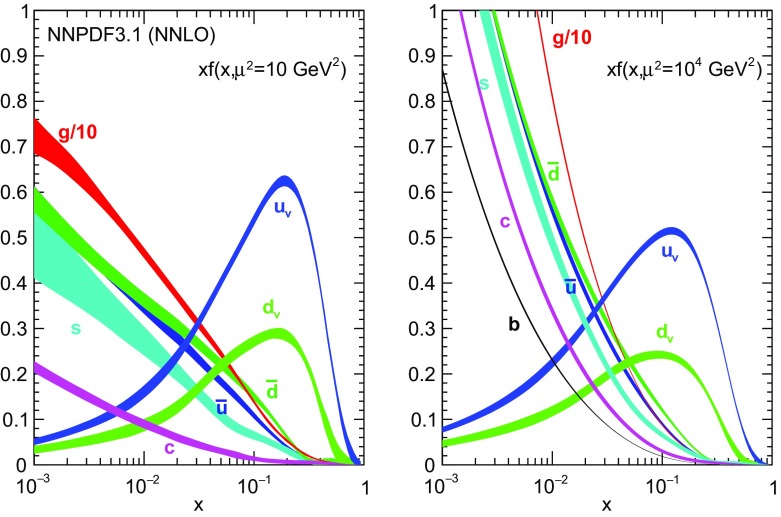
The NNPDF3.1 NNLO PDFs, evaluated at $\mu ^2=10~\mathrm{GeV}^2$ (left) and $\mu ^2=10^4~\mathrm{GeV}^2$ (right)

In Table 6 we provide values of $\chi ^2/N_{\mathrm{dat}}$ both for the global fit and individually for all the datasets included in the NNPDF3.1 LO, NLO and NNLO PDF determinations. These are compared with their NNPDF3.0 NLO and NNLO counterparts. The $\chi ^2$ is computed using the covariance matrix including all correlations, as published by the corresponding experiments. Inspection of this table shows that the fit quality improves from LO to NLO to NNLO: not only is there a significant improvement between LO and NLO, but there is also a marked improvement when going from NLO to NNLO. It is interesting to note that this was not the case in NNPDF3.0 where the fit quality at NNLO was in fact slightly worse than at NLO (see Table 9 of Ref. [5]). This reflects the increased proportion of hadronic processes included in NNPDF3.1, for which NNLO corrections are often substantial, and also, possibly, methodological improvements.

The overall fit quality with NNPDF3.1 is rather better than that obtained using NNPDF3.0 PDFs. Whereas this is clearly expected for LHC measurements which were not included in NNPDF3.0, it is interesting to note that the HERA measurements which were already present in 3.0 (though in a slightly different uncombined form) are also better fitted. The quality of the description with the previous NNPDF3.0 PDFs is nevertheless quite acceptable for all the new data, indicating a general compatibility between NNPDF3.0 and NNPDF3.1. Note that NNPDF3.0 values in Table 6 are computed using the NNPDF3.1 theory settings, thus in particular with different values of the heavy-quark masses than those used in the NNPDF3.0 PDF determination. Because of this, the NNPDF3.0 fit quality shown in Table 9 of Ref. [5] is slightly better than that shown in Table 6, yet even so the fit quality of NNPDF3.1 is better still. Specifically, concerning HERA data, the fit quality of NNPDF3.0 with consistent theory settings can be read off Table 7 of Ref. [124]: it corresponds to $\chi ^2/N_{\mathrm{dat}}=1.21$ thereby showing that indeed NNPDF3.1 provides a better description. The reasons for this improvement will be discussed in Sect. 3.4 below.

For many of the new LHC measurements, achieving a good description of the data is only possible at NNLO. The total $\chi ^2/N_{\mathrm{dat}}$ for the ATLAS, CMS and LHCb experiments is 1.09, 1.06 and 1.47 respectively at NNLO, compared with 1.36, 1.20 and 1.62 at NLO. The datasets exhibiting the largest improvement when going from NLO to NNLO are those with the smallest experimental uncertainties. For example the ATLAS *W*, *Z* 2011 rapidity distributions (from 3.70 to 2.14), the CMS 8 TeV $Z\, p_T$ distributions (from 3.65 to 1.32) and the LHCb 8 TeV $W,Z\rightarrow \mu$ rapidity distributions (from 1.88 to 1.37); in these experiments uncorrelated statistical uncertainties are typically at the sub-percent level. It is likely that this trend will continue as LHC measurements become more precise.

### Parton distributions

We now inspect the baseline NNPDF3.1 parton distributions, and compare them to NNPDF3.0 and to MMHT14 [7], CT14 [6] and ABMP16 [8]. The NNLO NNPDF3.1 PDFs are displayed in Fig. 8. It can be seen that although charm is now independently parametrized, it is still known more precisely than the strange PDF. The most precisely determined PDF over most of the experimentally accessible range of *x* is now the gluon, as will be discussed in more detail below.

**Fig. 9:**
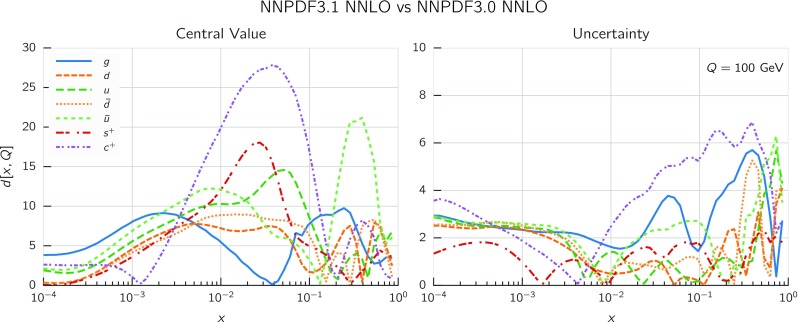
Distances between the central values (left) and the uncertainties (right) of the NNPDF3.0 and NNPDF3.1 NNLO PDF sets, evaluated at $Q=100$ GeV. Note the different in scale on the *y* axis between the two plots

In Fig. 9 we show the distance between the NNPDF3.1 and NNPDF3.0 PDFs. According to the definition of the distance given in Ref. [98], $d\simeq 1$ corresponds to statistically equivalent sets. Comparing two sets with $N_\mathrm{rep}=100$ replicas, a distance of $d\simeq 10$ corresponds to a difference of one sigma in units of the corresponding variance, both for central values and for PDF uncertainties. For clarity only the distance between the total strangeness distributions $s^+=s+\bar{s}$ is shown, rather than the strange and antistrange separately. We find important differences both at the level of central values and of PDF errors for all flavors and in the entire range of *x*. The largest distance is found for charm, which is independently parametrized in NNPDF3.1, while it was not in NNPDF3.0. Aside from this, the most significant distances are seen in light-quark distributions at large *x* and strangeness at medium *x*.

**Fig. 10:**
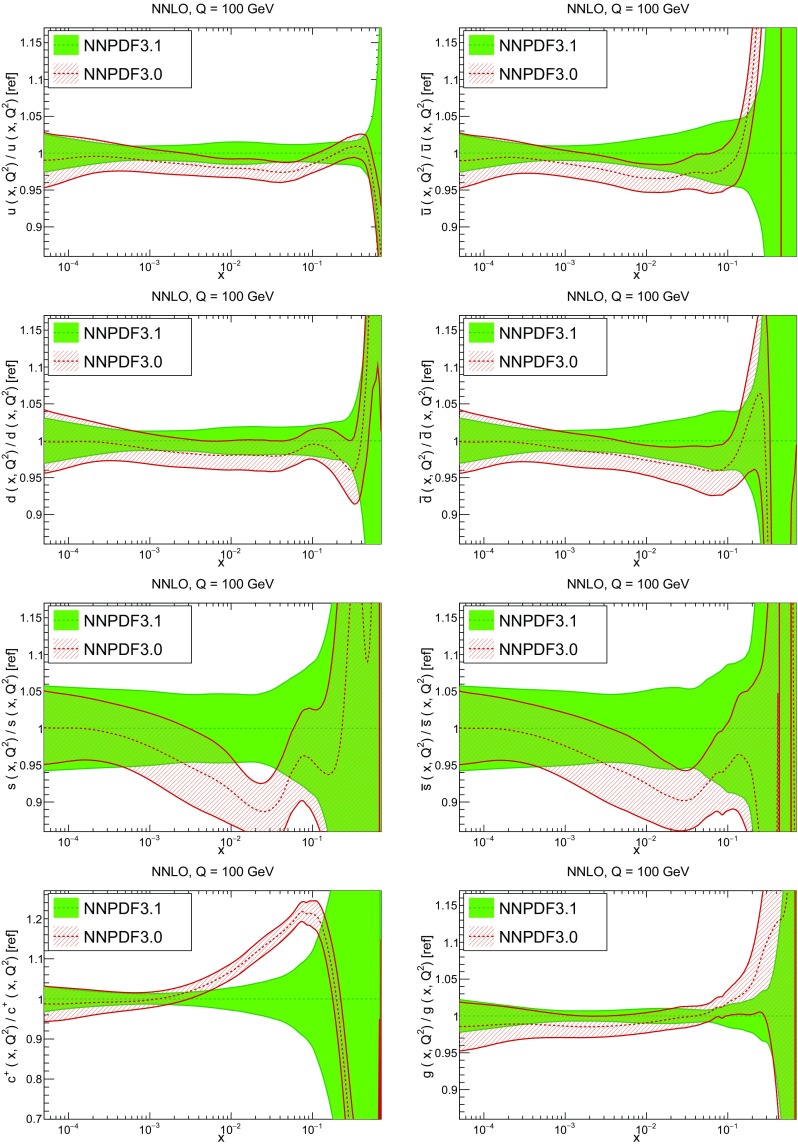
Comparison between NNPDF3.1 and NNPDF3.0 NNLO PDFs at $Q=100$ GeV. From top to bottom up and anti-up, down and antidown, strange and antistrange, charm and gluon are shown

In Fig. 10 we compare the full set of NNPDF3.1 NNLO PDFs with NNPDF3.0. The NNPDF3.1 gluon is slightly larger than its NNPDF3.0 counterpart in the $x\lesssim 0.03$ region, while it becomes smaller at larger *x*, with significantly reduced PDF errors. The NNPDF3.1 light quarks and strangeness are larger than NNPDF3.0 at intermediate *x*, with the largest deviation seen for the strange and antidown PDFs, while at both small and large *x* there is good agreement between the two PDF determinations. The best-fit charm PDF of NNPDF3.1 is significantly smaller in the intermediate-*x* region compared with the perturbative charm of NNPDF3.0, while at larger *x* it has significantly increased uncertainty.

**Fig. 11:**
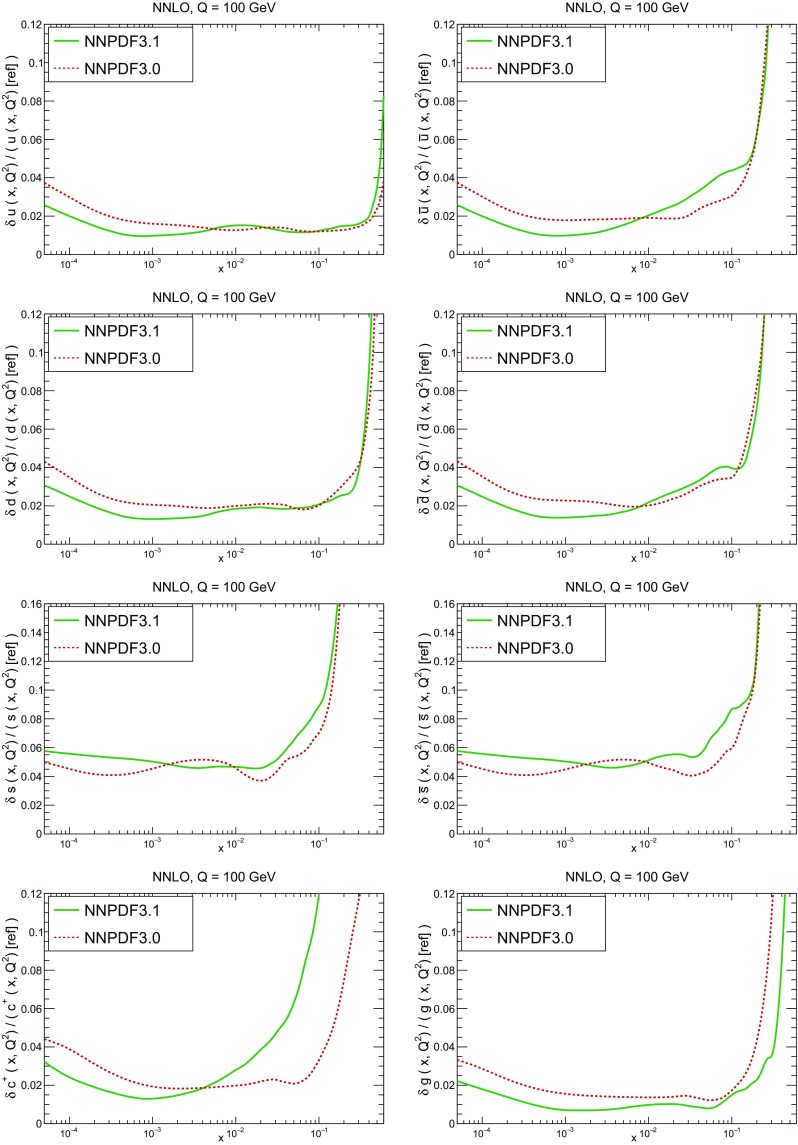
Comparison between NNPDF3.1 and NNPDF3.0 relative PDF uncertainties at $Q=100$; the PDFs are as in Fig. 10. The uncertainties shown are all normalized to the NNPDF3.1 central value

A detailed comparison of the corresponding uncertainties is presented in Fig. 11, where we compare the relative uncertainty on each PDF, defined as the ratio of the one-sigma PDF uncertainty to the central value of the NNPDF3.1 set. NNPDF3.1 uncertainties are either comparable to those of NNPDF3.0, or are rather smaller. The only major exception to this is the charm PDF at intermediate and large *x* for which uncertainties are substantially increased. On the other hand, the uncertainties in the gluon PDF are smaller in NNPDF3.1 over the entire range of *x*. This is an important result, since one may have expected generally larger uncertainties in NNPDF3.1 due to the inclusion of one additional freely parametrized PDF. The fact that the only uncertainty which has enlarged significantly is that of the charm PDF suggests that not parametrizing charm may be a source of bias. The fact that central values change by a non-negligible amount, though compatible within uncertainties, while the uncertainties themselves are significantly reduced, strongly suggests that NNPDF3.1 is more accurate than NNPDF3.0, as would be expected from the substantial amount of new data included in the fit. The effect of parametrizing charm on PDFs and their uncertainties will be discussed in more detail in Sect. 3.4, while the effects of the new data on both central values and uncertainties will be discussed in Sect. 4.1.

**Fig. 12:**
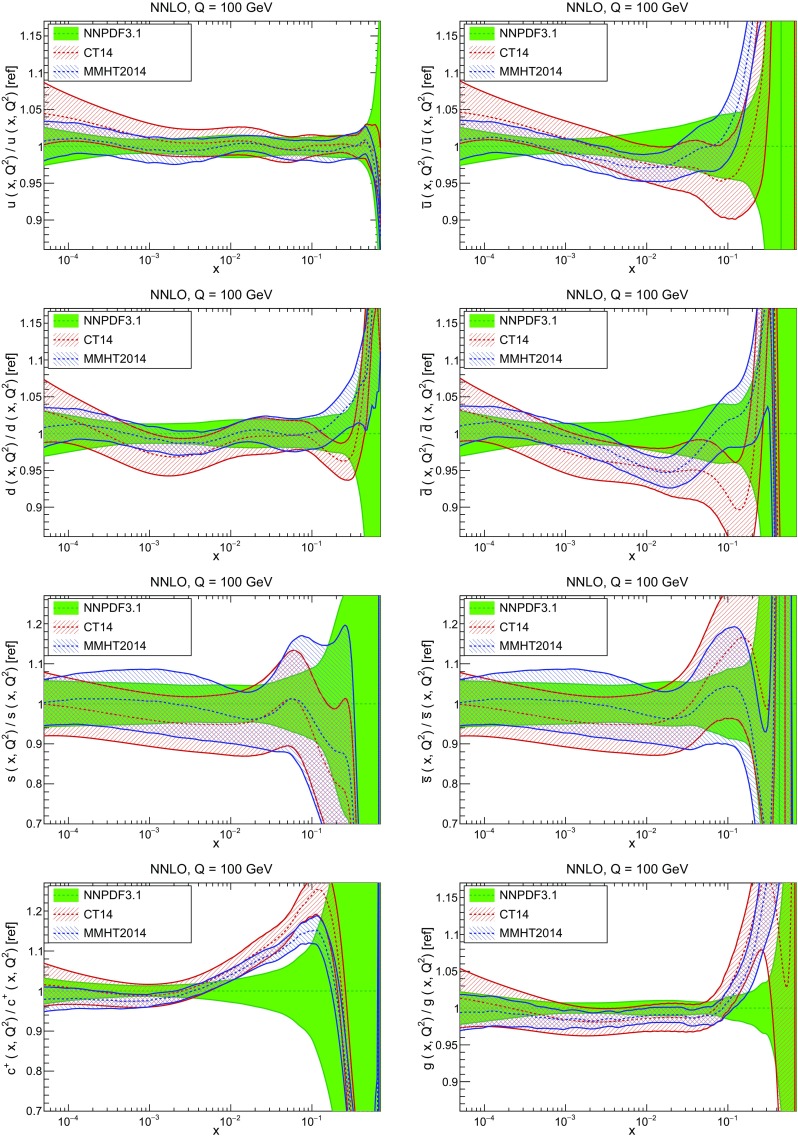
Comparison between NNPDF3.1, CT14 and MMHT2014 NNLO PDFs. The comparison is performed at $Q=100$ GeV, and results are shown normalized to the central value of NNPDF3.1; the PDFs are as in Fig. 10

**Fig. 13:**
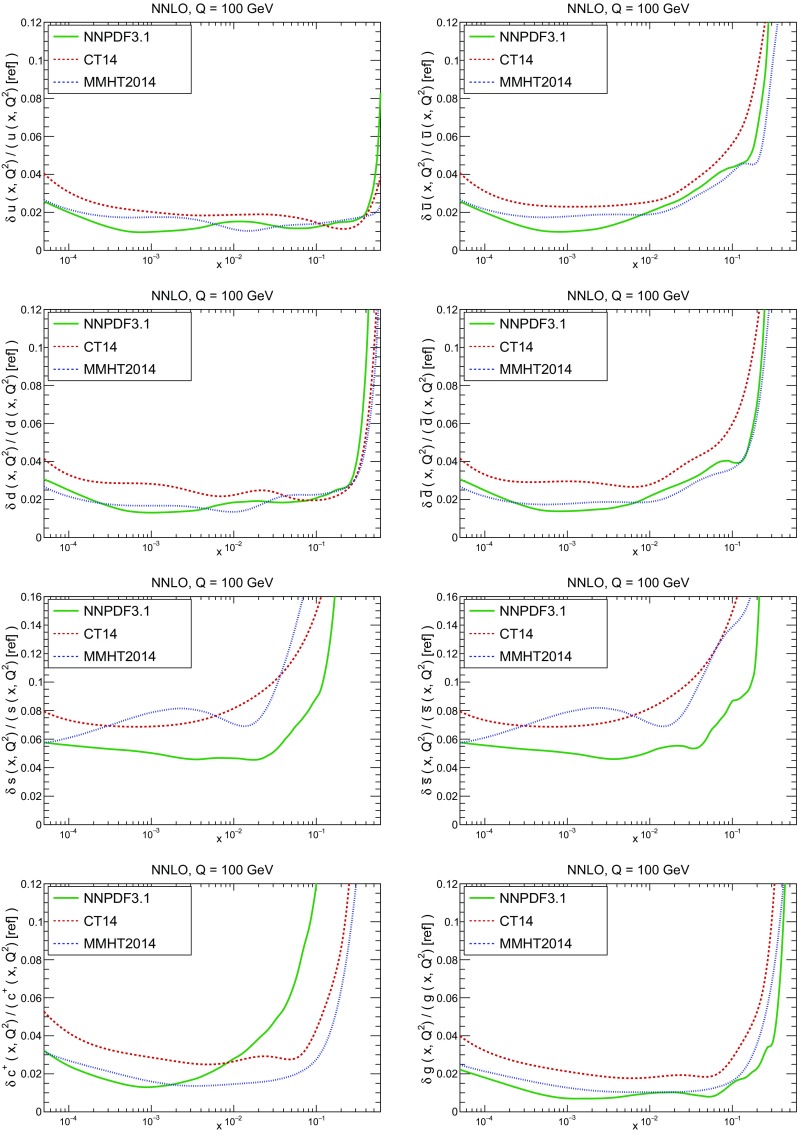
Comparison between NNPDF3.1, CT14 and MMHT2014 relative PDF uncertainties at $Q=100$; the PDFs are as in Fig. 12

In Fig. 12 we compare the NNPDF3.1 PDFs to the other global PDF sets included in the PDF4LHC15 combination along with NNPDF3.0, namely CT14 and MMHT14. This comparison is therefore indicative of the effect of replacing NNPDF3.0 with NNPDF3.1 in the combination. The relative uncertainties in the three sets are compared in Fig. 13. Comparing Fig. 12 to Fig. 10, it is interesting to observe that several aspects of the pattern of differences between NNPDF3.1 and the other global fits are similar to those between NNPDF3.1 and NNPDF3.0, and therefore they are likely to have a similar origin. This is particularly clear for the charm and gluon. The gluon in the region $x\lesssim 0.03$, relevant for Higgs production, is still in good agreement between the three sets. However, now NNPDF3.1 is at the upper edge of the one-sigma range, i.e. the NNPDF3.1 gluon in this region is enhanced. At large *x* the NNPDF3.1 gluon is instead suppressed in comparison to MMHT14 and CT14. As we will show in Sects. 3.4 and 4.1 the enhancement is a consequence of parametrizing charm, while as we will show in Sect. 4.3 the large-*x* suppression is a direct consequence of including the 8 TeV top differential data. The uncertainty in the NNPDF3.1 gluon PDF is now noticeably smaller than that of either CT14 or MMHT14.

For the quark PDFs, for up and down we find good agreement in the entire range of *x*. For the antidown PDF, agreement is marginal, with NNPDF3.1 above MMHT14 and CT14 for $x\lesssim 0.1$ and below them for larger *x*. The strange fraction of the proton is larger in NNPDF3.1 than CT14 and MMHT14, and has rather smaller PDF uncertainties. The best-fit NNPDF3.1 charm is suppressed at intermediate *x* in comparison to the perturbatively generated ones of CT14 and MMHT14, but has a much larger uncertainty at large *x* as would be expected, with the differences clearly traceable to the fact that in NNPDF3.1 charm is freely parametrized.

**Fig. 14:**
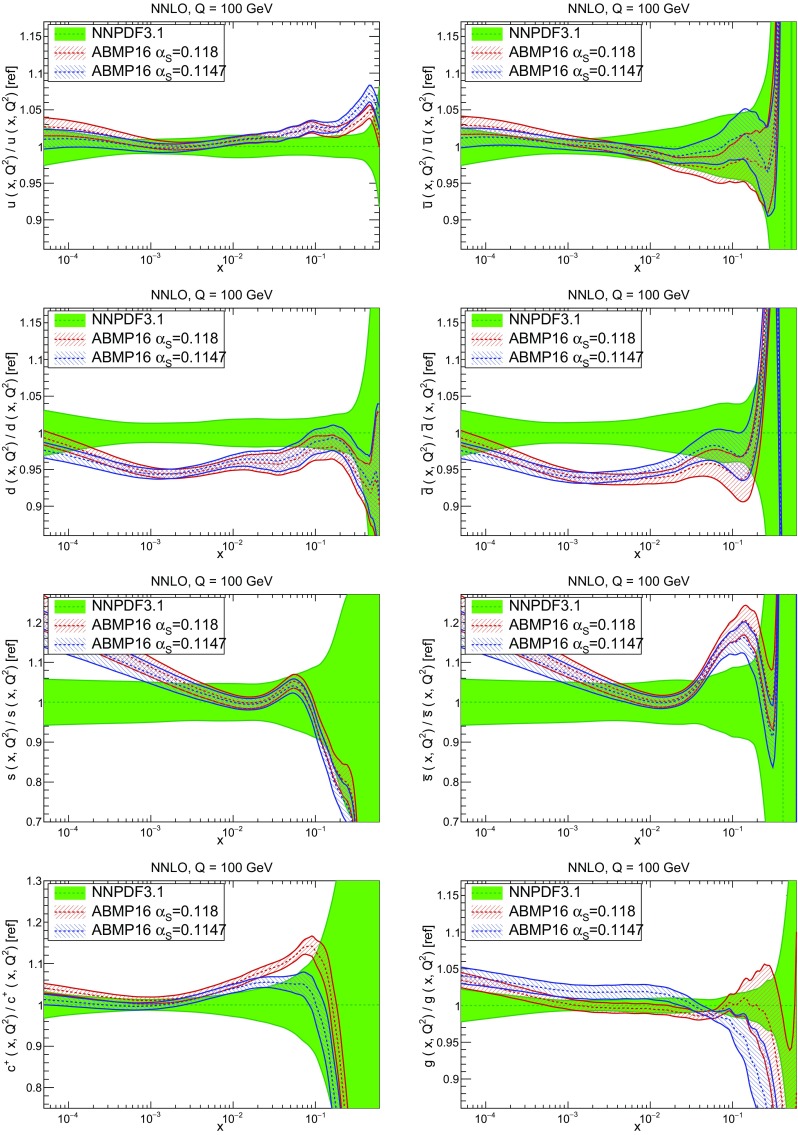
Same as Fig. 12 but now comparing to the ABMP16 NNLO $n_f=5$ sets both with their default $\alpha _s(m_Z)=0.1147$, and $\alpha _s(m_Z)=0.118$

Finally, in Fig. 14 we compare NNPDF3.1 to the recent ABMP16 set. This set is released in various fixed-flavor number schemes. Because we perform the comparison at a scale $Q^2=10^4$ GeV$^2$, we choose the $n_f=5$ NNLO ABMP16 sets, both with their default value $\alpha _s(m_Z)=0.1147$ and with $\alpha _s(m_Z)=0.118$. When a common value of $\alpha _s(m_Z)=0.118$ is adopted, there is generally reasonable agreement for the gluon PDF, except at large *x* where ABMP16 undershoots NNPDF3.1. Differences are larger in the case of light quarks: the ABMP16 up distribution overshoots NNPDF3.1 at large *x*, while the down quark undershoots in the whole *x* range. Differences are largest for the strange PDF, though comparing with Fig. 12 it is clear that ABMP16 differs by a similarly large amount from MMHT14 and CT14. In general the ABPM16 sets have rather smaller uncertainties than NNPDF3.1. This is especially striking for strangeness, where the difference in uncertainty is particularly evident. This is to be contrasted with the CT14 and MMHT14 sets, which have qualitatively similar uncertainties to NNPDF3.1 throughout the data region. The fact that the uncertainties for ABMP16 are so small can be traced to their overly restrictive parametrization, and the fact that this set is produced using a Hessian methodology, but unlike MMHT14 and CT14, with no tolerance (see Refs. [6, 7]).

### Methodological improvements: parametrizing charm

The main methodological improvement in NNPDF3.1 over NNPDF3.0 is the fact that the charm PDF is now parametrized in the same way as the light and strange quark PDFs. To quantify the effect of this change, we have performed a repeat of the NNPDF3.1 analysis but with charm treated as in all previous NNPDF PDF determinations, i.e., generated entirely perturbatively through matching conditions implemented at NLO or NNLO.

**Table 7:** Same as Table 6, but now comparing the default NNPDF3.1 NNLO and NNLO sets to the variant in which charm is perturbatively generated. For HERA $\sigma _c^\mathrm{NC}$ the number in parentheses refer to the subset of data to which the NNLO FC cut of Table 4 is applied

Dataset	NNPDF3.1 pert. charm		NNPDF3.1	
	NNLO	NLO	NNLO	NLO
NMC	1.38	1.38	1.30	1.35
SLAC	0.70	1.22	0.75	1.17
BCDMS	1.27	1.24	1.21	1.17
CHORUS	1.10	1.07	1.11	1.06
NuTeV dimuon	1.27	1.01	0.82	0.87
HERA I+II inclusive	1.21	1.15	1.16	1.14
HERA $\sigma _c^\mathrm{NC}$	1.20 (1.42)	1.21 (1.35)	1.45	1.15 (1.35)
HERA $F_2^b$	1.16	1.12	1.11	1.08
DYE866 $\sigma ^d_{\mathrm{DY}}/\sigma ^p_{\mathrm{DY}}$	0.46	0.48	0.41	0.40
DYE886 $\sigma ^p$	1.38	1.09	1.43	1.05
DYE605 $\sigma ^p$	1.05	0.83	1.21	0.97
CDF *Z* rap	1.44	1.46	1.48	1.62
CDF Run II $k_t$ jets	0.86	0.86	0.87	0.84
D0 *Z* rap	0.60	0.64	0.60	0.67
D0 $W\rightarrow e\nu$ asy	2.71	1.63	2.70	1.59
D0 $W\rightarrow \mu \nu$ asy	1.42	1.38	1.56	1.52
ATLAS total	**1.17**	**1.45**	**1.09**	**1.3**
ATLAS *W*, *Z* 7 TeV 2010	1.04	1.08	0.96	1.04
ATLAS high-mass DY 7 TeV	1.66	2.08	1.54	1.88
ATLAS low-mass DY 2011	0.83	0.70	0.90	0.69
ATLAS *W*, *Z* 7 TeV 2011	2.74	4.29	2.14	3.70
ATLAS jets 2010 7 TeV	0.96	0.95	0.94	0.92
ATLAS jets 2.76 TeV	1.06	1.13	1.03	1.03
ATLAS jets 2011 7 TeV	1.11	1.14	1.07	1.12
ATLAS $Z\, p_T$ 8 TeV $(p_T^{ll},M_{ll})$	0.94	1.19	0.93	1.17
ATLAS $Z\, p_T$ 8 TeV $(p_T^{ll},y_{ll})$	0.96	1.84	0.94	1.77
ATLAS $\sigma _{tt}^\mathrm{tot}$	0.80	2.03	0.86	1.92
ATLAS $t\bar{t}$ rap	1.39	1.18	1.45	1.31
CMS total	**1.09**	**1.2**	**1.06**	**1.20**
CMS *W* asy 840 pb	0.69	0.80	0.78	0.86
CMS *W* asy 4.7 fb	1.75	1.76	1.75	1.77
CMS $W+c$ tot	–	0.49	–	0.54
CMS $W+c$ ratio	–	1.92	–	1.91
CMS Drell–Yan 2D 2011	1.33	1.27	1.27	1.23
CMS *W* rap 8 TeV	0.90	0.65	1.01	0.70
CMS jets 7 TeV 2011	0.87	0.86	0.84	0.84
CMS jets 2.76 TeV	1.06	1.05	1.03	1.01
CMS $Z\, p_T$ 8 TeV $(p_T^{ll},y_{ll})$	1.29	3.50	1.32	3.65
CMS $\sigma _{tt}^\mathrm{tot}$	0.21	0.67	0.20	0.59
CMS $t\bar{t}$ rap	0.96	0.96	0.94	0.96
LHCb total	**1.48**	**1.77**	**1.47**	**1.62**
LHCb *Z* 940 pb	1.31	1.08	1.49	1.27
LHCb $Z\rightarrow ee$ 2 fb	1.47	1.66	1.14	1.33
LHCb $W,Z \rightarrow \mu$ 7 TeV	1.54	1.51	1.76	1.60
LHCb $W,Z \rightarrow \mu$ 8 TeV	1.51	2.28	1.37	1.88
Total dataset	**1.187**	**1.197**	**1.148**	**1.168**

In Table 7 we show the $\chi ^2/N_{\mathrm{dat}}$ values when charm is perturbatively generated at NLO and NNLO. Unsurprisingly the fit quality deteriorates when charm is not an independently parametrized PDF. This is what one would naively expect since perturbative charm imposes a constraint upon the fit, thereby reducing the number of free parameters.

However, it is interesting to observe that the fit quality to the inclusive HERA data (1306 data points) significantly deteriorates when going from NLO to NNLO with perturbative charm, whereas it remains stable when charm is independently parametrized. Concerning the charm structure function data, note that, as discussed in Sect. 2.2 above, a further cut is applied to the HERA $\sigma _c^\mathrm{NC}$ data at NNLO when charm is independently parametrized. In order to allow for a consistent comparison, in Table 7 we show in parentheses the value of $\chi ^2/N_{\mathrm{dat}}$ computed for the 37 (out of 47) data points that survive this cut also for all other cases. Hence, for this data the fit quality is similar to perturbative and parametrized charm, and also similar at NLO and NNLO (slightly worse at NNLO, by an amount compatible with a statistical fluctuation). The fact that when parametrizing charm there no longer is a deterioration of fit quality when going from NLO to NNLO suggests that this resolves a tension present at NNLO, with perturbative charm, between HERA and hadron collider data. Likewise, a purely perturbative charm leads to a substantial deterioration at NNLO for BCDMS, NMC and especially for the NuTeV dimuon cross-sections. This can be traced to the fact that independently parametrizing charm is essential to reconcile the HERA data with the constraints on the strange content of the proton imposed by the ATLAS *W*, *Z* 2011 rapidity distributions.

**Fig. 15:**
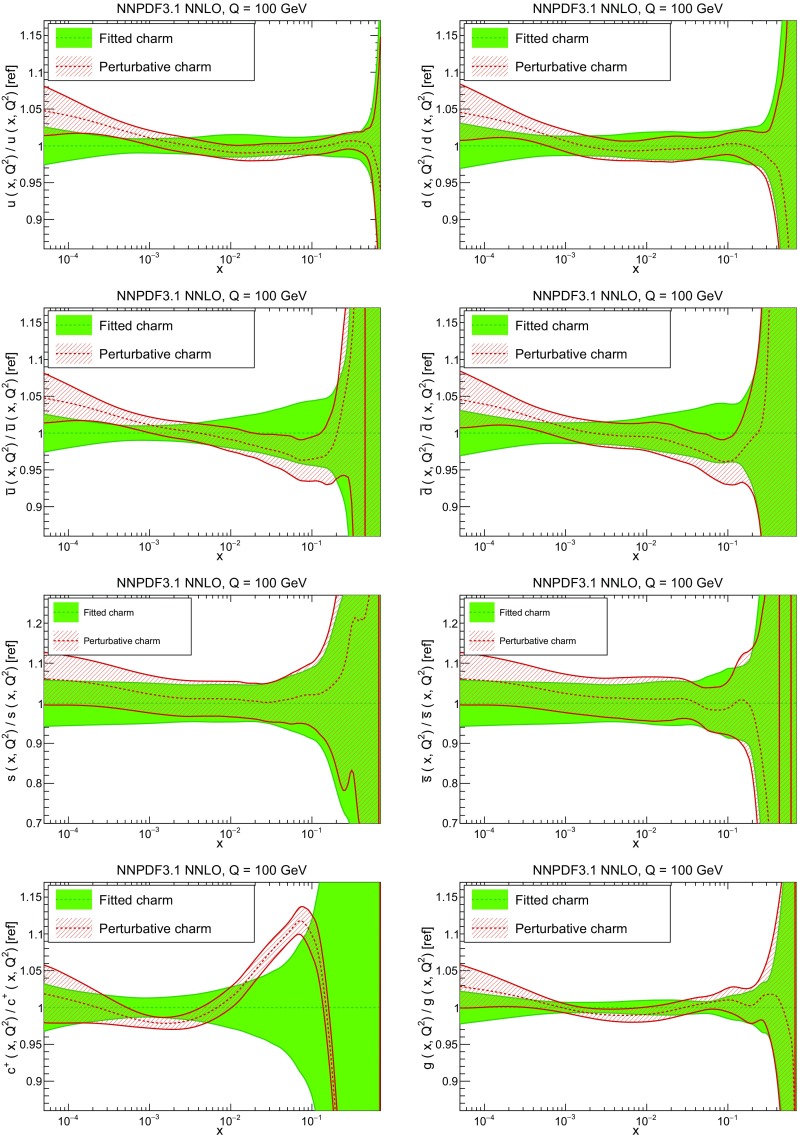
Comparison of NNPDF3.1 NNLO PDFs to a variant in which charm is generated entirely perturbatively (and everything else is unchanged)

In Fig. 15 we directly compare the PDFs with parametrized and perturbative charm. The light-quark PDFs and the gluon are generally enhanced for $x\gtrsim 0.003$ and reduced for smaller *x* when charm is independently parametrized. The largest differences can be seen in the up quark, while the strange and gluon distributions are more stable. The best-fit charm distribution has a distinctly different shape and significantly larger uncertainty than its perturbatively generated counterpart. As argued in Ref. [23] this shape might well be compatible with a charm PDF generated perturbatively at high perturbative orders.

**Fig. 16:**
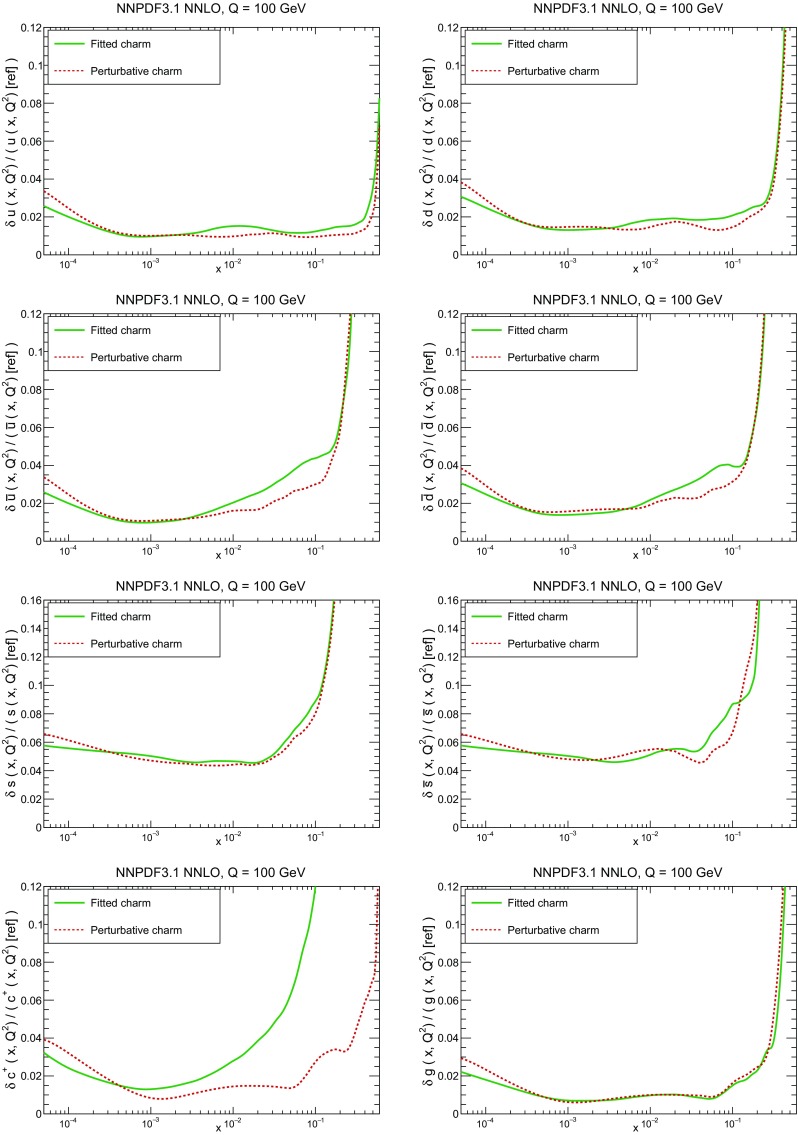
Comparison of the fractional one-sigma PDF uncertainties in NNPDF3.1 NNLO with the corresponding version where charm is generated perturbatively (and everything else is unchanged). The PDF comparison plot was shown in Fig. 15

In Fig. 16 we directly compare PDF uncertainties. It is remarkable that the uncertainties other than for charm are essentially unchanged when charm is independently parametrized, with only a slight increase in sea quark PDF uncertainties for $10^{-3}\lesssim x \lesssim 10^{-2}$. The uncertainty on the gluon is almost completely unaffected. The PDF uncertainty on charm when it is independently parametrized is in line with that of other sea quark PDFs, while the uncertainty of the perturbatively generated charm follows that of the gluon and is consequently much smaller.

**Fig. 17:**
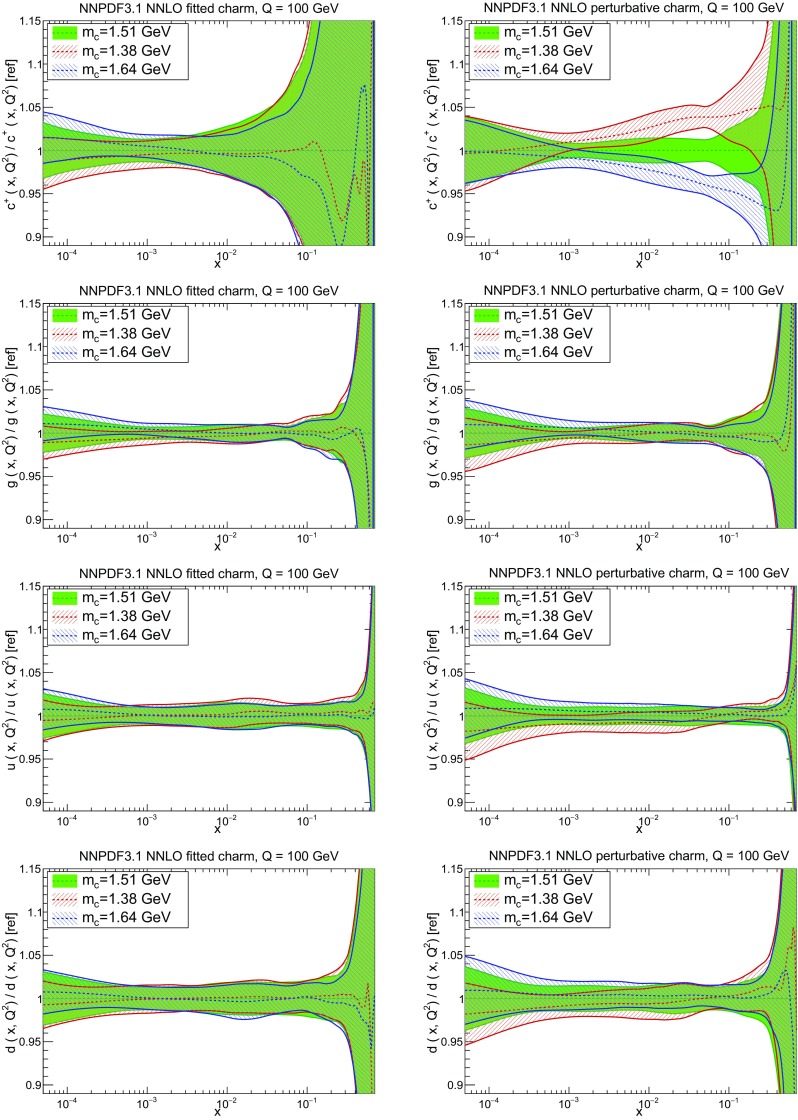
Dependence of the NNPDF3.1 NNLO PDFs on the charm mass. Results are shown both for parametrized charm (left) and perturbative charm (right), for (from top to bottom) charm, gluon, up and down PDFs

**Fig. 18:**
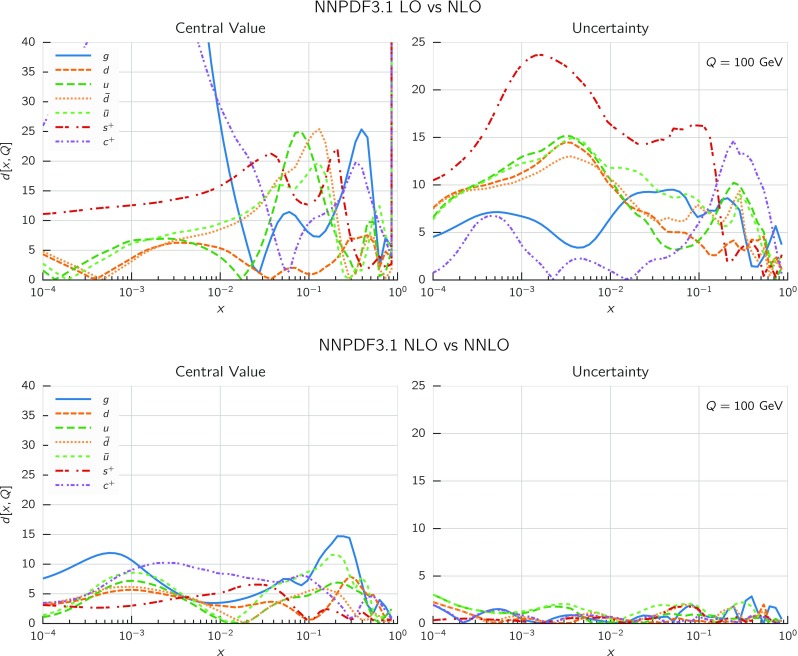
Distances between the LO and NLO (top) and the NLO and NNLO (bottom) NNPDF3.1 NNLO PDFs at $Q=100$ GeV. Note the difference in scale on the *y* axis between the two plots

**Fig. 19:**
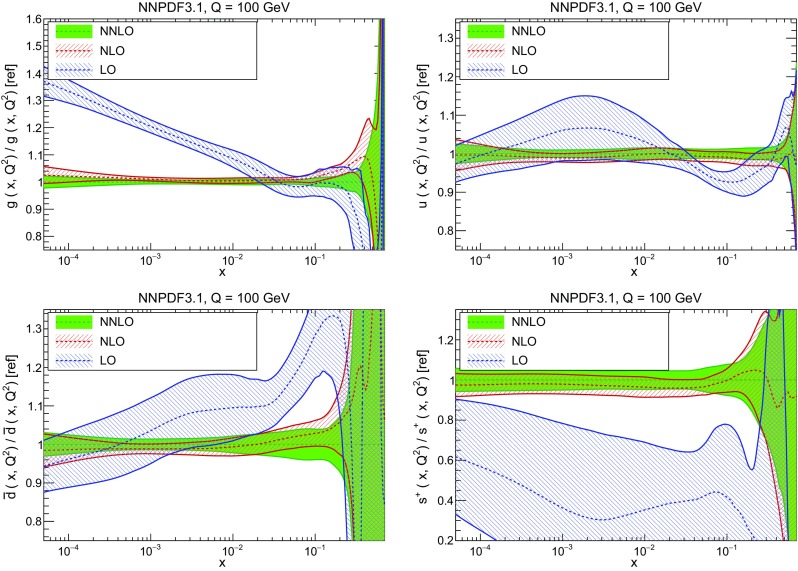
Comparison between some of the LO, NLO and NNPDF3.1 NNLO PDFs: gluon and up (top), antidown and total strangeness (bottom). All results are shown at $Q=100$ GeV, normalized to the NNLO central value

**Fig. 20:**
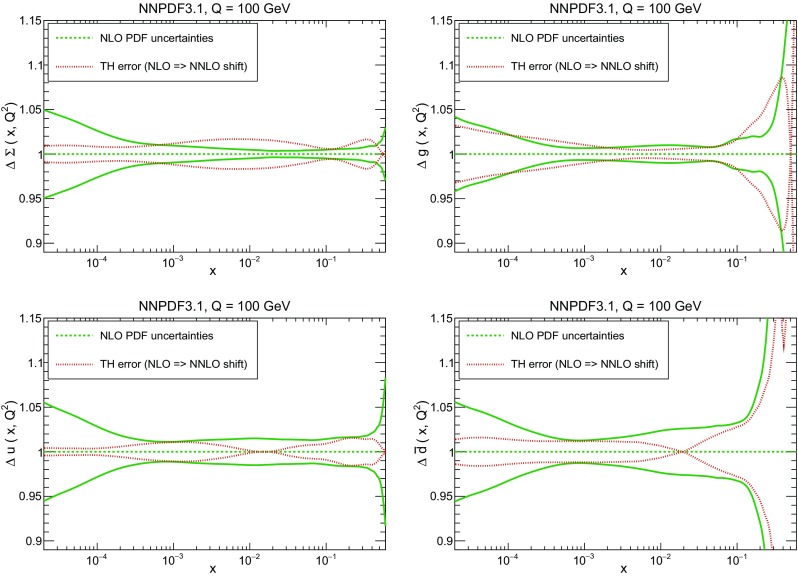
Comparison between the NLO PDF uncertainties and the shift between the NLO and NNLO PDFs. All results are shown as ratios to the NLO PDFs, for $Q=100$ GeV. The shift is symmetrized. We show results for the singlet, gluon (top); up and antidown (bottom) PDFs

**Fig. 21:**
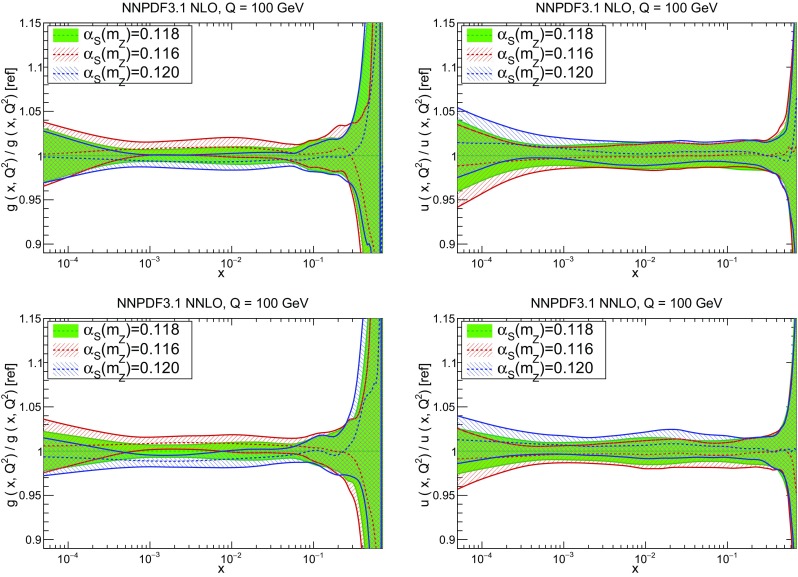
Dependence of NNPDF3.1 NLO (top) and NNLO (bottom) PDFs on the value of $\alpha _s$. The gluon (left) and up quark (right) are shown at $Q=100$ GeV, normalized to the central value

**Fig. 22:**
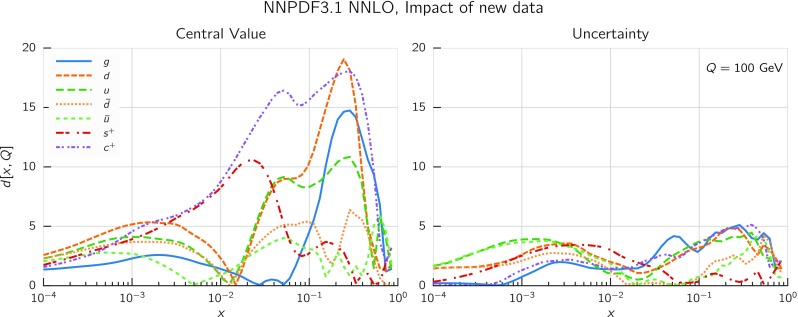
Same as Fig. 9, but now comparing the NNPDF3.1 NNLO global PDFs to PDFs determined using exactly the same methodology but with the NNPDF3.0 dataset

**Fig. 23:**
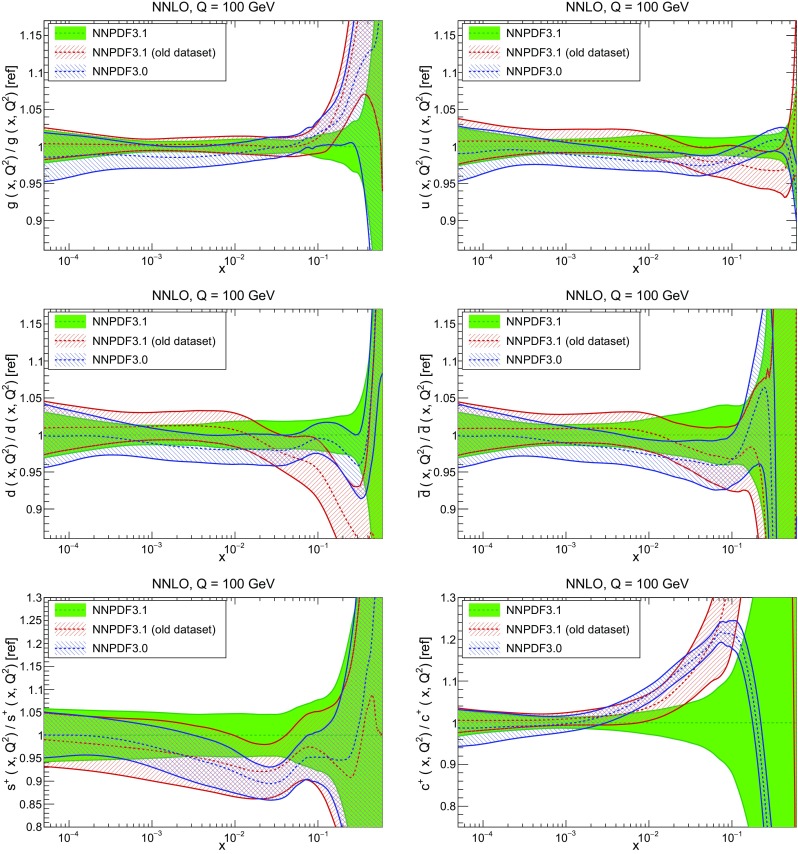
Same as Fig. 10, but now also including PDFs determined using NNPDF3.1 methodology with the NNPDF3.0 dataset. From left to right and from top to bottom the gluon, up, down, antidown, total strangeness and charm are shown

**Fig. 24:**
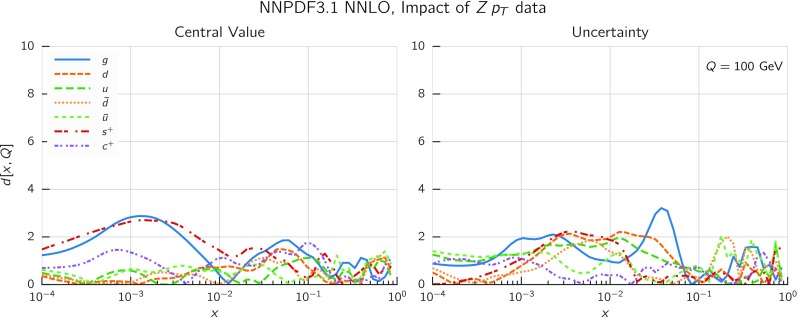
Same as Fig. 9, but now comparing the default NNPDF3.1 to a version of it with the 8 TeV $Z\, p_T$ data from ATLAS and CMS not included

**Fig. 25:**
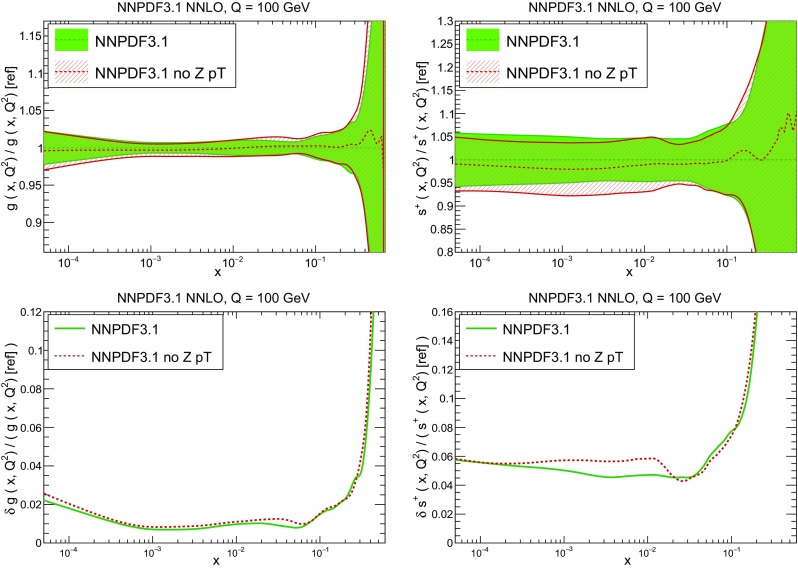
Same as Fig. 10 (top) and as Fig. 11 (bottom), but now comparing the default NNPDF3.1 to a version of it with the 8 TeV $Z\, p_T$ data from ATLAS and CMS not included. Results are shown for the gluon (left) and total strangeness (right)

A previous comparison of PDFs determined with parametrized or perturbative charm was presented in Ref. [23] and led to the conclusion that parametrizing charm and determining it from the data greatly reduces the dependence on the charm mass thereby reducing the overall PDF uncertainty when the uncertainty due to the charm mass is kept into account. As mentioned, NNPDF3.1 PDFs are determined using heavy-quark pole mass values and uncertainties recommended by the Higgs Cross-Section Working Group [131]. For charm, this corresponds to $m_\mathrm{c}^\mathrm{pole}=1.51 \pm 0.13\,\mathrm{GeV}$. In order to estimate the impact of this uncertainty, we have produced NNPDF3.1 NNLO sets with $m_\mathrm{c}^\mathrm{pole}=1.38$ GeV and $m_\mathrm{c}^\mathrm{pole}=1.64$ GeV. Results are shown in Fig. 17 for some representative PDFs, both for the default NNPDF3.1 and for the version with perturbative charm. It is clear that the very strong dependence of the charm PDF on $m_\mathrm{c}$ which is found when charm is perturbatively generated all but disappears when charm is independently parametrized. While the gluon is always quite stable, the dependence of perturbatively generated charm on $m_\mathrm{c}$ propagates to the light-quark distributions. These are therefore significantly stabilized by parametrizing charm. Indeed, if charm is generated perturbatively, the shift in up and down quark distributions upon one-sigma variation of the charm mass is comparable to (though somewhat smaller than) the PDF uncertainty. When charm is independently parametrized this dependence is considerably reduced. With parametrized charm, collider observables at high scales become essentially independent of the charm mass, in line with the expectation from decoupling arguments.

### Theoretical uncertainties

PDF uncertainties on global PDF sets entering the PDF4LHC15 combination consist only of the uncertainty propagated from experimental data and uncertainties due to the methodology. These can be controlled through closure testing. There are, however, further sources of uncertainty due to the theory used in PDF determination, which we briefly assess here. These can be divided into two main classes:

- Missing higher-order uncertainties (MHOU), arising due to the truncation of the QCD perturbative expansion at a given fixed order (LO, NLO or NNLO) in the theory used for PDF determination.
- Parametric uncertainties, due to the uncertainties on the values of parameters of the theory used for PDF determination: the main ones are the values of $\alpha _s(m_Z)$ and of $m_\mathrm{c}^\mathrm{pole}$.

A full assessment of MHOU is an open problem, which we leave to future investigations. For the time being, a first assessment can be obtained by studying the perturbative stability of our results. In Fig. 18 we show the distances at $Q=100$ GeV between all the PDFs in the LO and NLO sets, and in the NLO and NNLO sets. Some of the LO, NLO and NNLO PDFs are then compared directly in Fig. 19. Differences between the LO and NLO sets are very large, both for central values and uncertainties, the latter being substantial at LO due to the poor fit quality. The shift in quark PDFs can be as large as two sigma ($d\simeq 20$), while the gluon at small *x* is completely different between LO and NLO due to the fact that the singular small-*x* behavior of the quark to gluon splittings only starts at NLO, and due to the vanishing of gluon initiated DIS and DY processes at LO. On the other hand, when going from NLO to NNLO, PDF uncertainties are essentially unaffected. Central values are also reasonably stable: the largest shifts, in the large-*x* gluon and down quark and small-*x* gluon, remain at or below the one-sigma level.

A quantitative estimate of the MHOU can be obtained by computing the shift between the central values of the NLO and NNLO NNPDF3.1 PDFs. The result is shown in Fig. 20 for some PDF combinations. In the plot, the shift has been symmetrized, and is compared with the NLO standard PDF uncertainty. In the quark singlet $\Sigma$ for $x\lesssim 10^{-3}$ the shift is larger than the PDF uncertainty, while it is smaller for individual flavors (as illustrated by the two quark distributions shown). This suggests that, for individual quark flavors and the gluon at NNLO, MHOU can be reasonably neglected at the current level of precision. However, for particular combinations (such as the singlet at small *x*) it is unclear whether MHOU can be neglected even at NNLO, given that at NLO they are larger than the PDF uncertainty.

We finally turn to parametric uncertainties. As we have discussed in Sect. 3.4, the dependence of PDFs upon the charm mass is almost entirely removed by parametrizing charm. The dependence on the *b*-quark mass is minor, except for the bottom PDFs themselves [94, 132]. Therefore, the only significant residual parametric uncertainty is on the value of the strong coupling. This uncertainty is routinely included along with the PDF uncertainty; in order to do this consistently, one needs PDF sets produced with different central values of $\alpha _s$ (see e.g. Ref. [12]). We have determined NNPDF3.1 NLO and NNLO PDFs with $\alpha _s(m_Z)$ varied in the range $0.108\le \alpha _s(m_Z)\le 0.124$ (see Sect. 6.2).

In Fig. 21 we compare the up and gluon PDFs as $\alpha _s(m_Z)$ is varied by $\Delta \alpha _s=\pm 0.002$ about its central value. As is well known, the gluon is anti-correlated to $\alpha _s(m_Z)$ at small and medium *x*, but positively correlated to it at large *x*. The dependence on $\alpha _s$ is rather milder for quark PDFs, with positive correlation at small *x*, and very little dependence altogether at large *x*.

## The impact of the new collider data

We now study the dependence of the NNPDF3.1 PDF set upon the experimental information on which it is based. Firstly we disentangle the effects of new data from the effects of methodological changes. Then we systematically quantify the impact on PDFs of each new piece of experimental information added in NNPDF3.1. Finally we discuss PDF determinations based on particular data subsets; PDFs determined only from collider data (i.e. excluding all fixed-target data), only from proton data (i.e. excluding all nuclear data), or excluding all LHC data. As these PDF sets based on reduced dataset can also be useful for specific phenomenological applications, they are also made available (see Sect. 6.2 below). As in the previous section, here we will only present a selection of representative plots, the interested reader is referred to a much larger set of plots available online as discussed in Sect. 6.2.

### Disentangling the effect of new data and methodology

In Sect. 3.4 we have studied the impact of the main methodological improvement introduced in NNPDF3.1, namely, independently parametrizing the charm PDF and determining it from the data. In order to completely disentangle the effect of data and methodology we have performed a PDF determination using NNPDF3.1 methodology, but the NNPDF3.0 dataset: specifically, we have removed from the NNPDF3.1 dataset all the new data. There remain some small residual differences between this restricted dataset and that of NNPDF3.0, specifically in some small differences in cuts and in the use of the combined HERA data instead of the separate HERA-I and HERA-II sets. However, these differences are expected to be minor [93].

In Fig. 22 we show the distances between the NNPDF3.1 NNLO PDF set, and that based on the NNPDF3.0 dataset using the same methodology. We see that the impact of the new data is mostly localized at large *x*, for the up, down and charm quarks and the gluon, and at medium *x* for strangeness. As far as uncertainties are concerned, we observe improvements of up to half a sigma across a wide range in *x* and for all PDF flavors. In Fig. 23 we compare some representative PDFs for NNPDF3.1, the set based on NNPDF3.0 data with NNPDF3.1 methodology, and the original NNPDF3.0. We see that the overall effect of the new data and the new methodology are comparable, but that they act in different regions and for different PDFs. For instance, for the light quarks and the gluon the impact of the new methodology dominates for all $x\lesssim 10^{-2}$, where it produces an enhancement, and specifically the enhancement of the gluon for $x\lesssim 0.03$, which was discussed in Sect. 3.3. At large *x* instead the dominant effect is from the new data, which lead to a reduction of the gluon and an enhancement of the quarks. Whereas of course charm is very significantly affected by the change in methodology—it was not independently parametrized in NNPDF3.0 – for $x\gtrsim 0.1$, the new data also have a big impact. In fact, while strangeness is mostly affected by the new data in the medium and small *x* regions, charm and gluon are most affected by them at large *x*.

### The transverse momentum of the *Z* boson

The use of transverse momentum distributions has been advocated for a long time (see e.g. Ref. [2]) as a clean and powerful constraint on PDFs, particularly the gluon. As discussed in Sect. 2, it is now possible to include such data at NNLO thanks to the availability of the computation of this process up to NNLO QCD, along with precise data on $Z\, p_T$ from ATLAS and CMS at 8 TeV. The impact of this dataset on PDFs has recently been studied in detail in Ref. [121].

NNPDF3.1 is the first global PDF determination to include this data. In order to assess the impact of this dataset, we have repeated the NNLO determination, excluding all $Z\, p_T$ data. In Fig. 24 we show the distances between this PDF set and the default: it is clear that the effect on all PDFs is moderate, with changes below one third of a sigma. The largest differences are seen in the gluon, as expected, and the strange distributions. The reason for this state of affairs can be best understood by directly comparing PDFs and their uncertainties; see Fig. 25. It is clear that central values move very little while uncertainties are slightly reduced, therefore demonstrating the excellent consistency of the constraint from these measurements with the existing dataset. The $Z\, p_T$ dataset therefore reinforces the reliability of our gluon determination. It also reduces somewhat the uncertainty on the total strangeness. While in Ref. [121] this dataset was found to have a rather stronger impact than shown here, it should be noted that this was the case when determining PDFs from the NNPDF3.0 dataset (less the jet data). In NNPDF3.1 more data are added, specifically top-pair differential distributions: a smaller impact of the $Z\, p_T$ dataset when added to a wider prior is not unexpected.

**Table 8:** The values of $\chi ^2/N_{\mathrm{dat}}$ for the LHC $Z\, p_T$ data using the NNPDF3.1 NNLO PDF set, and for a new PDF determination which also includes the ATLAS $Z\, p_T$ 7 TeV data

	NNPDF3.1 NNLO	+ ATLAS $Z\, p_T$ 7 TeV data
ATLAS $Z\, p_T$ 7 TeV $(p_T^{ll},y_{ll})$	[6.78]	3.40
ATLAS $Z\, p_T$ 8 TeV $(p_T^{ll},M_{ll})$	0.93	0.98
ATLAS $Z\, p_T$ 8 TeV $(p_T^{ll},y_{ll})$	0.93	1.17
CMS $Z\, p_T$ 8 TeV $(p_T^{ll},M_{ll})$	1.32	1.33

**Fig. 26:**
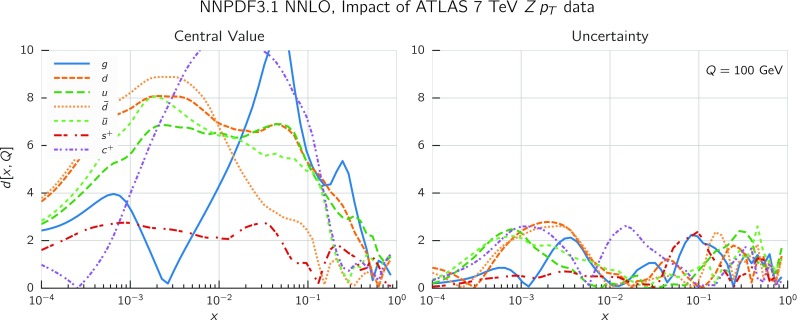
Same as Fig. 9, but now comparing the default NNPDF3.1 to a version of it with the 7 TeV $Z\, p_T$ ATLAS data also included

**Fig. 27:**
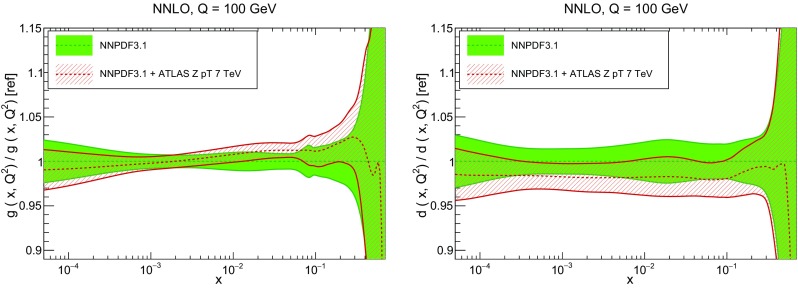
Same as Fig. 10 but now comparing the default NNPDF3.1 to a version of it with the 7 TeV $Z\, p_T$ ATLAS data also included. Results are shown for the gluon (left) and down quark (right)

In addition to the 8 TeV measurements from ATLAS and CMS there also exists a measurement of the normalized distribution at 7 TeV from ATLAS. The inclusion of this dataset is problematic because the covariance matrix for a normalized distribution depends on the cuts imposed on the dataset, and only the covariance matrix for the full dataset is available. This issue was studied in detail in Ref. [121]. Furthermore, this dataset is superseded by the more precise 8 TeV measurement. Therefore, it has not been included in the NNPDF3.1 dataset. However, we have studied its potential impact by including it in a dedicated PDF determination, with its nominal published covariance matrix unmodified despite the cuts.

In Table 8 we provide the $\chi ^2/N_{\mathrm{dat}}$ values for all the LHC $Z\, p_T$ measurements, for the NNPDF3.1 NNLO baseline (including the ATLAS and CMS $Z\, p_T$ 8 TeV data), and also from the determination including the ATLAS $Z\, p_T$ 7 TeV data.

In the first column, the value of the $\chi ^2/N_{\mathrm{dat}}$ for the 7 TeV data is in parentheses to indicate that, unlike all other values, it is a prediction and not the outcome of a fit. It is clear that the ATLAS $Z\, p_T$ 7 TeV dataset is very poorly reproduced by the default NNPDF3.1 set, and even after its inclusion in the dataset it cannot be accommodated. In fact, its inclusion is accompanied by a deterioration in the fit quality to ATLAS 8 TeV data, which are more accurate and supersede them. Furthermore, there are also indications of tension between this dataset and the ATLAS *W*/*Z* rapidity distributions, whose total $\chi ^2$ deteriorates by 12 units (with 46 datapoints). The distances between these two PDF sets, displayed in Fig. 26, show that the gluon and quarks are shifted by almost one sigma by the inclusion of the ATLAS $Z\, p_T$ 7 TeV data. This is explicitly shown in Fig. 27 for the gluon and down quark. It is apparent that uncertainties are, however, almost unchanged by the inclusion of this dataset.

**Fig. 28:**
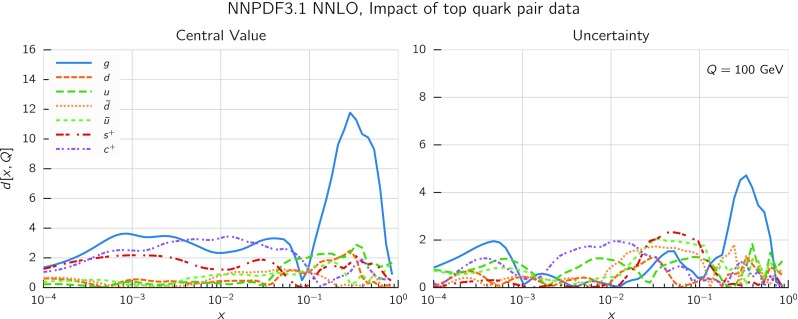
Same as Fig. 24 but now excluding all top data (total cross-sections and differential distributions). Note the different scale on the *y* axis in the left plot

While we cannot say how a better treatment of the covariance matrix would affect the results, we must conclude that within our current level of understanding, inclusion of the ATLAS 7 TeV $Z\, p_T$ dataset would have a significant impact on PDFs, without an improvement in precision, and with signs of tension between this dataset and both the remaining $Z\, p_T$ datasets, and the other *W* and *Z* production data. Therefore its inclusion in the global dataset does not appear to be justified.

### Differential distributions for top pair production

The impact of differential top-pair production on PDFs and the optimal selection of top datasets has been discussed extensively in Ref. [124]. Here we briefly study the impact of the top data on NNPDF3.1 by comparing with PDFs determined removing the top data from the dataset. In Fig. 28 we show the distances between these PDF sets. Large differences can be seen in the gluon central value and uncertainty for $x\gtrsim 0.1$: these data constrain the gluon for values as large as $x\simeq 0.6$ [124], a region in which constraints from other processes are not available. The effect on other PDFs is moderate, with the largest impact seen on charm at small *x*.

**Fig. 29:**
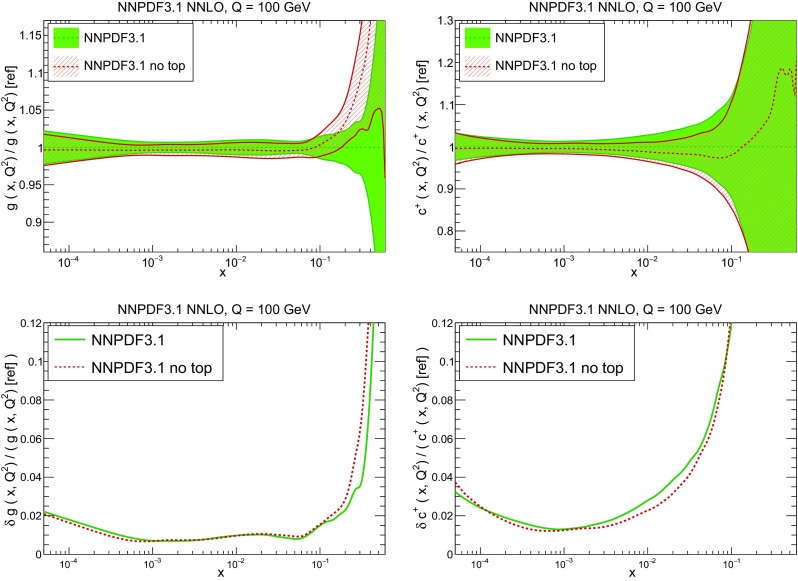
Same as Fig. 25 but now excluding all top data (total cross-sections and differential distributions). Results are shown for the gluon (left) and charm (right), the PDFs above and their uncertainties below

The differences between the two PDF sets are demonstrated in Fig. 29, where the gluon and the charm quark are shown. There is a substantial reduction in the uncertainty of the large *x* gluon, with the central value without top data being considerably higher than the narrow error band of the result when top is included. This suggests a significant increase in the precision of the gluon determination due to the top data. For the large *x* gluon the differences between NNPDF3.1 and NNPDF3.0 seen in Figs. 10, 11, 12, 13, 14, 15, 16, 17, 18, 19, 20, 21, 22 and 23 are therefore partly driven by the top data. The impact on quark PDFs is marginal, as can be seen in the case of charm.

As already mentioned in Sect. 2.7, it has been shown in Ref. [125] that the sensitivity of the rapidity distribution on the top mass is minimal. In fact, in Ref. [124] it was shown that if the top mass is varied by 1 GeV, NLO theoretical predictions for the normalized rapidity distributions at the LHC 8 TeV vary by 0.6% at most in the kinematic range covered by the data, which is much less than the uncertainty on the data, or the size of the NNLO corrections. This strongly suggests that our results are essentially independent of the value of the top mass.

### Inclusive jet production

While jet data have been used for PDF determination for a long time, their full NNLO treatment is only becoming possible now, thanks to the recent completion of the relevant computation [20, 108]. However, as discussed in Sect. 2.4, NNLO corrections are not yet available for all datasets included in NNPDF3.1. Consequently in the default NNPDF3.1 PDF determination, jets have been included using NNLO PDF evolution and NLO matrix elements supplemented by an extra theory uncertainty determined through scale variation. Here we assess generally the effect of jet data, and in particular the possible impact of this approximation.

**Fig. 30:**
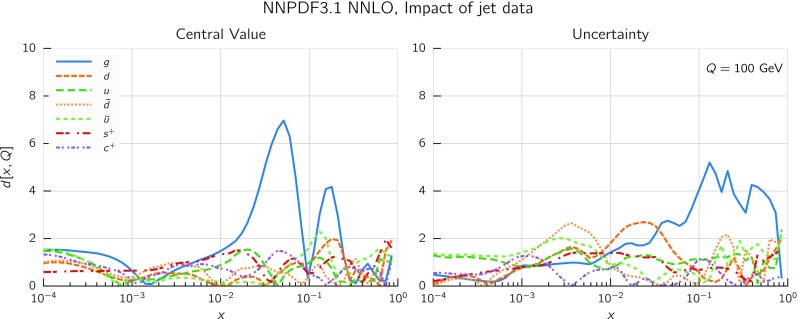
Same as Fig. 24 but now excluding all jet data

**Fig. 31:**
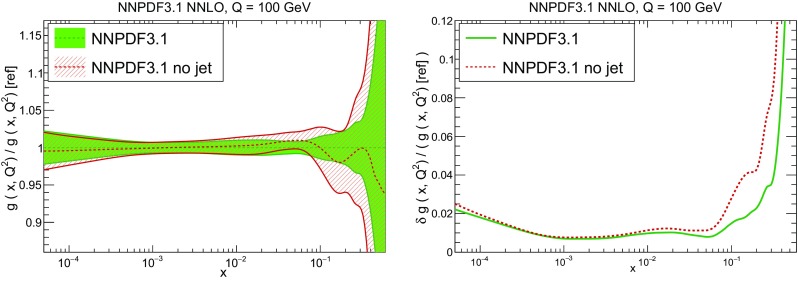
Comparison between the default NNPDF3.1 NNLO PDFs an alternative determination in which all jet data have been removed: the gluon (left) and the percentage uncertainty on it (right) are shown

To this end, we first repeat the NNPDF3.1 determination but excluding jet data. The distances between these PDFs and the default are shown in Fig. 30. It is clear that jet data have a moderate and very localized impact, on the gluon in the region $0.1\lesssim x\lesssim 0.6$, at most at the half-sigma level, with essentially no impact on other PDFs. The changes in all other PDFs are compatible with a statistical fluctuation. A direct comparison of the gluon PDFs and their uncertainties in Fig. 31 confirms this. The uncertainty on the gluon is reduced by up to a factor of two by the jet data in this region, with the central value of the gluon within the narrower uncertainty band of the default set.

It is interesting to observe that in NNPDF3.0 the impact of the jet data was rather more significant, with uncertainties being reduced by a large factor for all $x\gtrsim 0.1$. In NNPDF3.1 the gluon at large *x* is strongly constrained by the top data, as discussed in Sect. 4.3. Specifically, the addition of the jet data leaves the gluon unchanged in this region, see Fig. 31, but addition of the top data produces a significant shift, as seen in Fig. 29. This suggests excellent compatibility between the jet and top data, with the large *x* gluon now mostly determined by the top data. This also explains the insensitivity to the NNLO correction to jet production, to be discussed shortly.

**Fig. 32:**
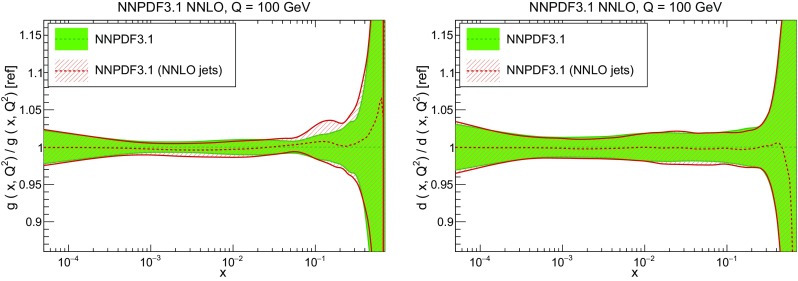
Same as Fig. 10 but now comparing the default NNPDF3.1 NNLO PDFs to an alternative determination in which ATLAS and CMS 7 TeV jet data have been included using exact NNLO theory. The gluon (left) and down (right) PDFs are shown

Despite their reduced impact, the jet data still play a non-negligible role. One may therefore worry about the reliability of the theoretical treatment, based on NLO matrix elements with theory uncertainties. In order to assess this, we have repeated the PDF determination but now using full NNLO theory in the case of the 2011 7 TeV LHC jet data where it is available. The other jet datasets, namely the CDF Run II $k_T$ jets, the ATLAS and CMS $\sqrt{s}=2.76$ TeV datasets, and the ATLAS 2010 7 TeV dataset, are treated as in the baseline. Essentially no change in PDFs is found, as illustrated in Fig. 32 where the gluon and down PDFs are shown. Such a result is consistent with the percent-level NNLO corrections found when using our choice of the jet $p_T$ as the central scale, shown in Fig. 2.4.

Also, as mentioned in Sect. 2.4, only the central rapidity bin of the ATLAS 2011 7 TeV data has been included, because we have found that, while a good description can be achieved if each of the rapidity bins is included in turn, or if the uncertainties are decorrelated between rapidity bins, it is impossible to achieve a good description of all rapidity bins with correlations included. One may therefore wonder whether the inclusion of other rapidity bins would lead to different results for the PDFs, despite the fact that they have less PDF sensitivity [104]. In order to check this, we have compared with the data the prediction for all of the ATLAS 2011 7 TeV data using the default NNPDF3.1 set, and determined the $\chi ^2$ for each rapidity bin separately. For the five rapidity bins which have not been included, from central to forward, we find $\chi ^2/N_\mathrm{dat}=1.27,\>0.95,\>1.06,\>0.97,\>0.73$, with, respectively, $N_\mathrm{dat}=29,\>26,\>23,\>19,\>12$, to be compared with the value $\chi ^2/N_{\mathrm{dat}}=1.06$ for $N_{\mathrm{dat}}=31$ of Table 9 for the central rapidity bin which is included. We conclude that all rapidity bins are well reproduced, and thus none of them can have a significant pull on PDFs that might change the result if they were included.

**Fig. 33:**
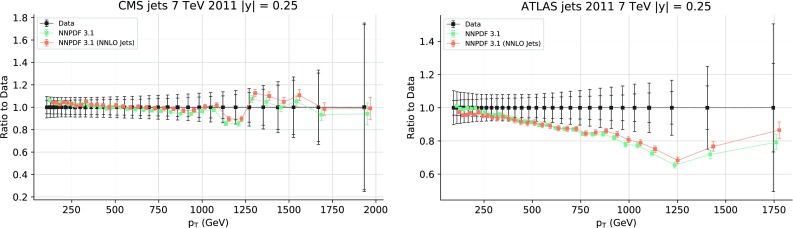
Comparison between CMS (left) and ATLAS (right) one-jet inclusive data at 7 TeV from 2011, and best-fit results obtained using NLO theory supplemented by scale uncertainties or exact NNLO theory. The uncertainties shown on the best-fit prediction is the PDF uncertainty, while that on the data is the diagonal (outer error bar) and the scale uncertainty on the NLO prediction (inner error bar)

In order to understand better the impact of the NNLO corrections and their effect on the PDFs, in Fig. 33 we compare the best-fit prediction to the 7 TeV 2011 CMS and ATLAS data for the two PDF sets compared in Figs. 30, 31, and 32. The corresponding values of $\chi ^2/N_{\mathrm{dat}}$ are collected in Table 9, both for these and all other jet data. First of all, one should note that, for all experiments for which an exact NNLO computation is not yet available, listed on the top part of Table 9, the $\chi ^2$ values obtained with these two PDF sets are almost identical. Because the predictions for these experiments are computed using the same theory (NLO with extra scale uncertainty), this shows that the change in PDFs is very small. For the experiments for which NNLO theory is available, $\chi ^2$ values also change very little. A comparison of the data to theory (see Fig. 33) shows that the NLO and NNLO predictions are, as expected, quite close. Nevertheless, the NNLO prediction is in slightly better agreement with the data, and this is reflected by the better value of the NNLO $\chi ^2$ shown in Table 9, despite the extra scale uncertainty (shown as an inner error bar on the data in Fig. 33) that is added to the NLO prediction only.

As a final check, we have repeated the PDF determination but with a cut excluding large $p_T$ jet data, for which the NNLO corrections are larger (compare Fig. 3). Specifically, we have only kept 7 TeV jet data with $p_T\le 240$ GeV, 2.76 TeV jet data with $p_T\le 95$ GeV, 1.96 TeV jet data with $p_T\le 68$ GeV. We found that the ensuing PDFs are statistically equivalent (distances of order one–two) to those from the default set. We conclude that the impact of the approximate treatment of jet data on the default NNPDF3.1 set is very small.

**Table 9:** The values of $\chi ^2/N_{\mathrm{dat}}$ for all the jet datasets obtained using either of the two PDF sets compared in Fig. 32, and in each case the theory used in the corresponding PDF determination. For the datasets in the top part of the table the exact NNLO computations are not yet available and NLO theory with scale uncertainty is used throughout, while for those in the bottom part of the table NNLO theory is used for the right column

	NNPDF3.1	exact NNLO
CDF Run II $k_t$ jets	0.84	0.85
ATLAS jets 2.76 TeV	1.05	1.03
CMS jets 2.76 TeV	1.04	1.02
ATLAS jets 2010 7 TeV	0.96	0.95
ATLAS jets 2011 7 TeV	1.06	0.91
CMS jets 7 TeV 2011 7 TeV	0.84	0.79

### Electroweak boson production in the forward region

**Fig. 34:**
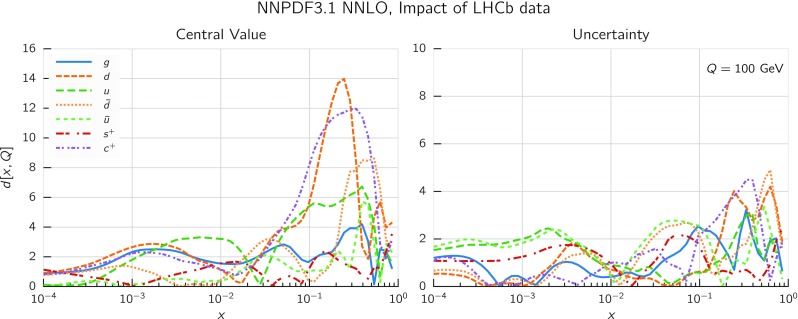
Same as Fig. 24 but now excluding all LHCb data. Note the different scale on the *y* axis in the left plot

**Fig. 35:**
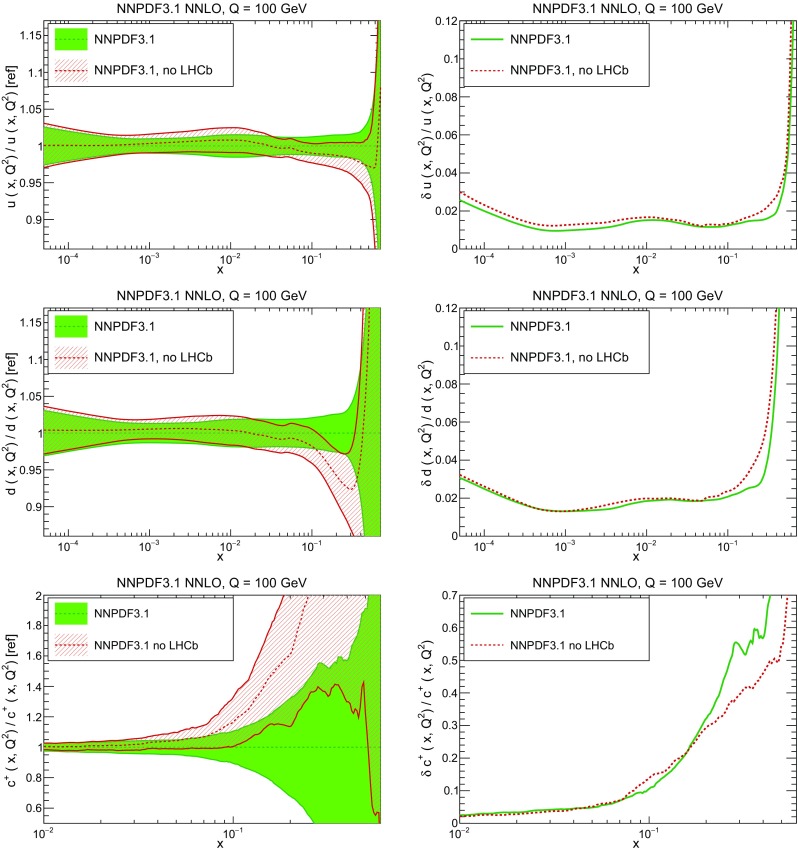
Same as Fig. 25 but now excluding all LHCb data. Results are presented, from top to bottom, for the up, down and charm PDFs. Both PDFs (left) and uncertainties (right) are shown

**Fig. 36:**
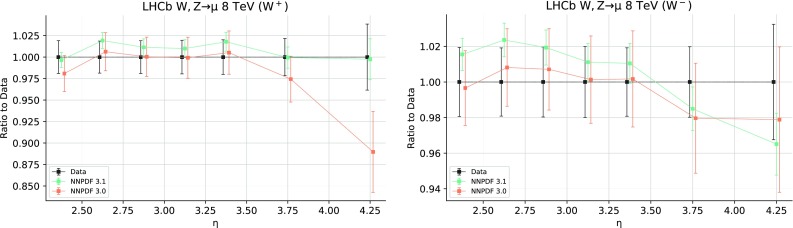
Comparison between 8 TeV LHCb muon $W^+$ (left) and $W^-$ (right) production data to NNLO predictions obtained using NNPDF3.1 and NNPDF3.0. The uncertainties shown are the diagonal experimental uncertainty for the data, and the PDF uncertainty for the best-fit prediction

**Fig. 37:**
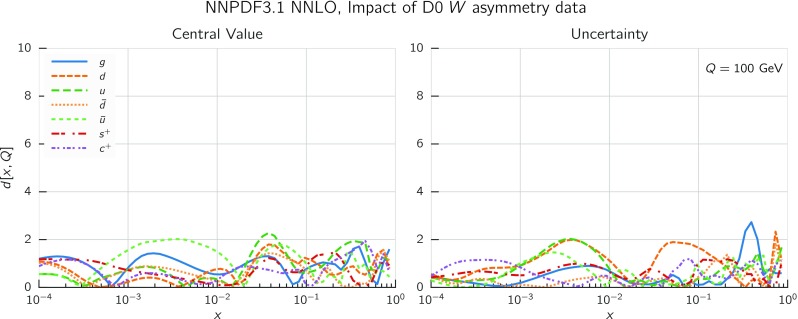
Same as Fig. 24 but now excluding D0 *W* asymmetry data

**Fig. 38:**
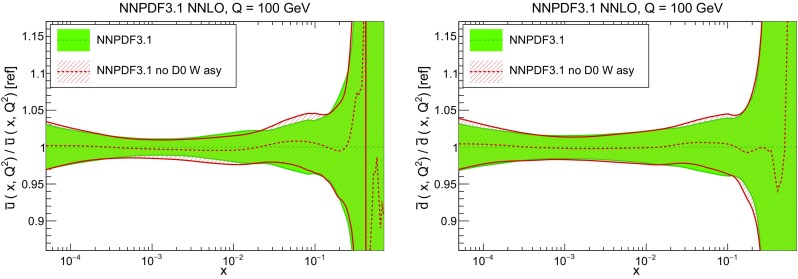
Same as Fig. 25 but now excluding D0 *W* asymmetries. The anti-up (left) and antidown (right) PDFs are shown

**Fig. 39:**
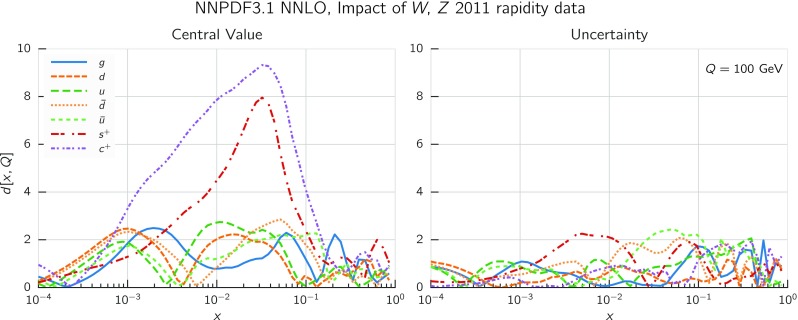
Same as Fig. 24 but now excluding 2011 ATLAS *W*, *Z* rapidity distributions

**Fig. 40:**
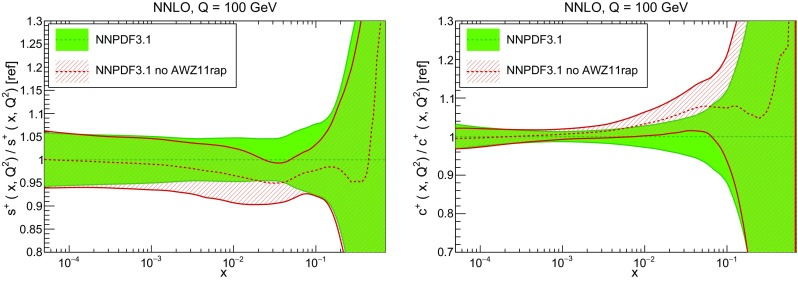
Same as Fig. 25 but now excluding 2011 ATLAS *W*, *Z* rapidity distributions. The total strange (left) and charm (right) PDFs are shown

**Fig. 41:**
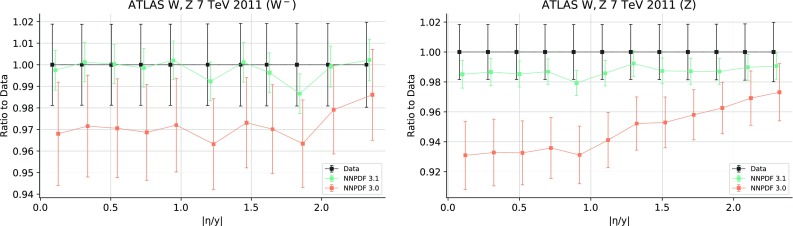
Comparison between the 2011 ATLAS 7 TeV $W^-$ (left) and *Z* (right) data to NNLO predictions obtained using NNPDF3.1 and NNPDF3.0; *W* production data are plotted versus the pseudo-rapidity of the forward lepton $\eta _l$, while *Z* production data are plotted vs. the dilepton rapidity $y_{ll}$

Electroweak production data from the LHCb experiment open up a new kinematic region and therefore provide new constraints on flavor separation at large and small *x*. While LHCb data were included in NNPDF3.0, the release of the legacy Run I LHCb measurements at 7 TeV and 8 TeV, which include all correlations between *W* and *Z* data, greatly increases their utility in PDF determination. In Fig. 34 distances are shown between the NNPDF3.1 NNLO default and PDFs determined excluding all LHCb data. The impact is significant for all quark PDFs, especially in the valence region: hence this data has a substantial impact on flavor separation, most notably at large *x*.

This is explicitly demonstrated for the up, down and charm PDFs in Fig. 35, where the percentage PDF uncertainties are also shown. The LHCb data play a significant role in the data-driven large-*x* enhancement of light-quark PDFs discussed in Sect. 4.1 (see Fig. 23), and are largely responsible for the sizable impact of new data on charm for $x\gtrsim 0.1$, where they significantly reduce the uncertainty. Effects are more marked at medium and large *x*, peaking at around $x\simeq 0.3$: in this region the PDF uncertainty is also substantially reduced; the reduction in uncertainty is especially marked for the down PDF.

In order to see the impact of the LHCb data directly, in Fig. 36 we compare the 8 TeV LHCb muon $W^+$ and $W^-$ data to predictions obtained using NNPDF3.0 and NNPDF3.1. The improvement is clear, particularly for large rapidities. There is also a noticeable reduction in PDF uncertainty on the prediction.

### *W* asymmetries from the Tevatron

*W* production data from the Tevatron have for many years been the leading source of information on quark flavor decomposition. The final legacy D0 *W* asymmetry measurements in the electron and muon channels are included in NNPDF3.1, superseding all previous data. In Fig. 37 we perform a distance comparison between the default NNPDF3.1 and PDFs determined excluding this dataset. Distances are generally small, an observation confirmed by direct PDF comparison in Fig. 38. However, we have seen in Table 6 that the fit quality for this dataset is rather better with NNPDF3.1 than with the previous NNPDF3.0. The moderate impact of this dataset is due to its excellent consistency with the abundant LHC data, which are now driving flavor separation. This data thus provides further evidence for the reliability of the flavor separation in NNPDF3.1.

### The ATLAS *W*, *Z* production data and strangeness

ATLAS *W* and *Z* production data were already included in NNPDF3.0, but recent measurements based on the 2011 dataset [72] have much smaller statistical uncertainties. This dataset, like the previous ATLAS measurement, has been claimed to have a large impact on strangeness. This is borne out by the plot, Fig. 39, of the distance between the default NNPDF3.1 and a version from which this dataset has been excluded. Indeed the largest effect—almost at the one-sigma level on central values—is seen on the strange and charm PDFs, with a rather smaller impact on all other PDFs.

A direct comparison of the strange and charm PDFs in Fig. 40 shows that strangeness is significantly enhanced in the medium/small *x* region by the inclusion of the ATLAS data, while charm is suppressed. As discussed in Sect. 3.4 and shown in Fig. 15, this suppression of charm cannot be accommodated when charm is perturbatively generated, and therefore parametrizing charm is important in order to be able to reconcile the ATLAS data with the global dataset which in this *x* range is severely constrained by HERA data. The strange and charm content of the proton will be discussed in detail in Sects. 5.2 and 5.3 below.

**Fig. 42:**
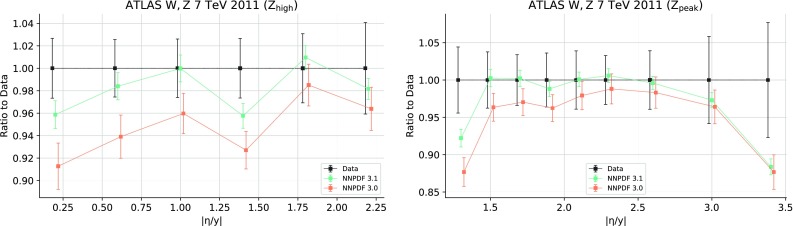
Same as Fig. 41 but now for two of the four data bins which have not been included in the NNPDF3.1 determination: high-mass *Z* production at central rapidity (left) and on-shell *Z* production at forward rapidity (right)

NNPDF3.1 achieves a good description of the data, as illustrated in Fig. 41 where the NNLO prediction obtained using NNPDF3.0 is also shown. It is clear that the agreement is greatly improved, as demonstrated in the $\chi ^2$ values shown in Table 6. It is also interesting to note the significant reduction in PDF uncertainties. As mentioned in Sect. 2.5, these data have been included only partially in the NNPDF3.1 determination: specifically *Z* production data off peak or at forward rapidity have not been included. We have checked, however, that the description of these data in NNPDF3.1 is equally good, and similarly improved in comparison to NNPDF3.0, with $\chi ^2/N\mathrm{dat}$ of order unity. A comparison of these data for two of the four bins which have not been included into the NNPDF3.1 and NNPDF3.0 is shown in Fig. 42.

### The CMS 8 TeV double-differential Drell–Yan distributions

Like ATLAS, CMS has also published updated electroweak boson production data. The NNPDF3.0 PDF determination already included double-differential (in rapidity and invariant mass) Drell–Yan data at 7 TeV from the CMS 2011 dataset [54]. An updated version of the same measurement at 8 TeV based on 2012 data was presented in Ref. [84], including both the absolute cross-sections and the ratio of 8 TeV and 7 TeV measurements.

**Fig. 43:**
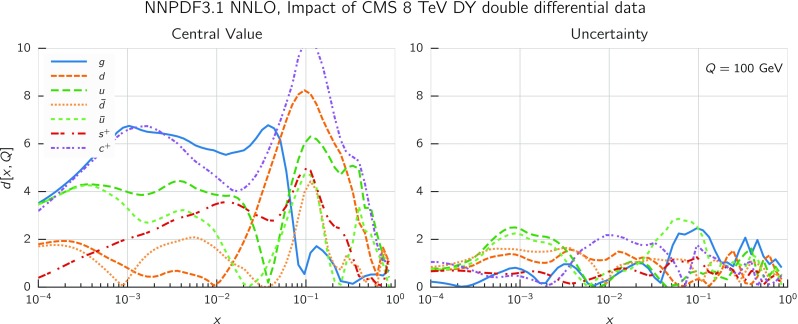
Same as Fig. 9 but now comparing the default NNPDF3.1 to a version of it with the 8 TeV CMS double-differential Drell–Yan data also included.

This data has very small uncorrelated systematic uncertainties. Unfortunately, only the full covariance matrix, with no breakdown of individual correlated systematics, has been made available. The combination of these two facts makes it impossible to include this experiment in the NNPDF3.1 dataset, as we now explain. In Fig. 43 we show the distances between the NNPDF3.1 NNLO PDF set and a modified version of it where this dataset has been included. While the impact on uncertainties is moderate, clearly this dataset has a significant impact at the level of central values on all PDFs for almost all *x* values, with a particularly important impact on the medium/small *x* gluon. This is somewhat surprising, given that Drell–Yan production only provides an indirect handle on the gluon PDF.

**Fig. 44:**
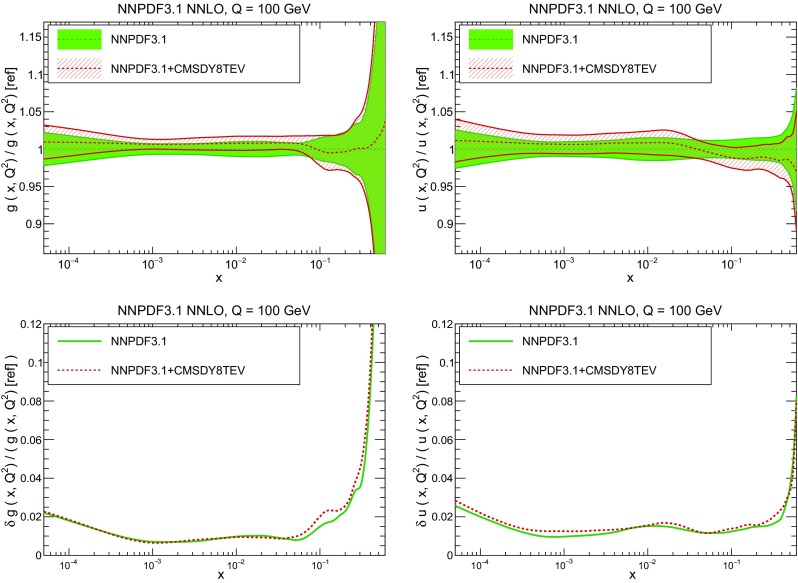
Same as Fig. 10 (top) but now comparing the default NNPDF3.1 to a version of it with the 8 TeV CMS double-differential Drell–Yan data also included. The corresponding percentage uncertainties are also shown (bottom). Results are shown for the gluon (left) and up quark (right)

A direct comparison of PDFs and their uncertainties in Fig. 44 shows that these data induce an upwards shift by up to one sigma of the gluon for $x\lesssim 0.1$, and a downward shift of the light-quark PDFs for $x\gtrsim 0.1$, by a comparable amount. This, however, is not accompanied by a reduction of PDF uncertainties, which increase a little, as also shown in Fig. 44.

Furthermore, while the fit quality of the 8 TeV CMS double-differential Drell–Yan data remains poor after their inclusion in the fit, with a value of $\chi ^2/N_{\mathrm{dat}}=2.88$, there is a certain deterioration in fit quality of all other experiments. Indeed, the total $\chi ^2$ to all the other data deteriorates by $\Delta \chi ^2=11.5$. A more detailed inspection shows that the most marked deterioration is seen in the HERA combined inclusive DIS data, with $\Delta \chi ^2=19.7$. This means that there is tension between the CMS data and the rest of the global dataset, and more specifically tension with the HERA data, which are most sensitive to the small *x* gluon.

We must conclude that this experiment appears to be inconsistent with the global dataset, and particularly with the data with which we have the least reasons to doubt, namely the combined HERA data and their determination of the gluon. In the absence of more detailed information on the covariance matrix it is not possible to further investigate the matter, and the 8 TeV CMS double-differential Drell–Yan data have consequently not been included in the global dataset.

**Fig. 45:**
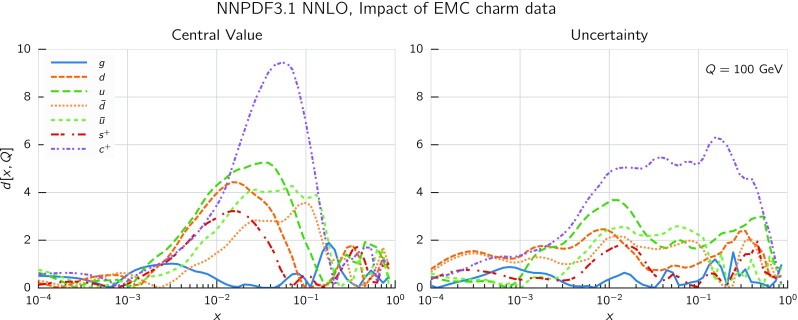
Same as Fig. 26, but now comparing the default NNPDF3.1 to a version of it with the EMC $F_2^c$ dataset also included

### The EMC $F_2^c$ data and intrinsic charm

The advantages of introducing an independently parametrized charm PDF were advocated in Ref. [23], where a first global PDF determination including charm was presented, based on the NNPDF3.0 methodology and dataset. The default NNPDF3.0 dataset was supplemented by charm deep-inelastic structure function data from the EMC Collaboration [69]. This dataset is quite old, but it remains the only measurement of the charm structure function in the large *x* region. With the wider NNPDF3.1 dataset the EMC dataset is no longer quite so indispensable, specifically in view of phenomenology at the LHC: it has thus been omitted from the default NNPDF3.1 determination as doubts have been raised about its reliability.

**Fig. 46:**
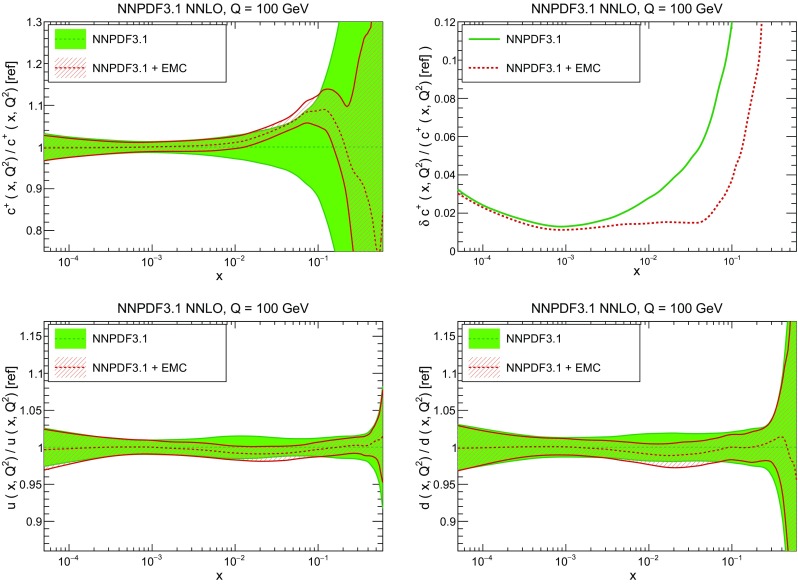
Same as Fig. 27 but now comparing the default NNPDF3.1 to a version of it with the EMC $F_2^c$ dataset also included. Results are shown for the charm (top left), up (bottom left) and down (bottom right) PDFs. The relative PDF uncertainty on charm is also shown (top right)

However, a number of checks performed in Refs. [23, 95], such as variations of kinematical cuts and systematic uncertainties, do not suggest any serious compatibility issues, and rather confirmed this dataset as being as reliable as the other older datasets with fixed nuclear targets routinely included in global PDF determinations. Therefore it is interesting to revisit the issue of the impact of this dataset within the context of NNPDF3.1. To this purpose we have produced a modified version of the global NNPDF3.1 NNLO analysis in which the EMC dataset [69] is added to the default NNPDF3.1 dataset. In Fig. 45 the distances between this PDF set and the default are shown. The EMC dataset has a non-negligible impact on charm, at the one-sigma level, and also, to a lesser extent, at the half-sigma level, on all light quarks, with only the gluon left essentially unaffected. The bulk of the effect is localized in the region $0.01\lesssim x\lesssim 0.3$. The PDFs are directly compared in Fig. 46. The EMC data lead to an increase in the charm distribution towards the upper edge of its error band in the default PDF set for $0.02\lesssim x\lesssim 0.2$, while reducing the uncertainty on it by a sizable factor. The light-quark PDFs are correspondingly slightly suppressed, and their uncertainties also reduced a little.

The values of $\chi ^2/N_{\mathrm{dat}}$ for the deep-inelastic scattering experiments and for the total dataset before and after inclusion of the EMC data in the NNPDF3.1 dataset are shown in Table 10. The fit quality of the EMC charm dataset is greatly improved by its inclusion, without any significant change of the fit quality for any other DIS data: the hadron collider data are even less sensitive. The inclusion of EMC charm data therefore appears to give a more accurate charm determination, with no cost elsewhere, and so usage of this PDF set is recommended when precise charm PDFs at large *x* are required. The phenomenological implications of the charm PDF will be discussed in Sect. 5.3 below.

### The impact of LHC data

**Fig. 47:**
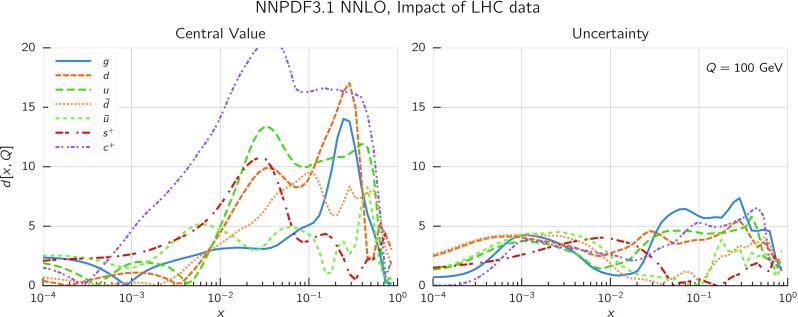
Same as Fig. 24 but now excluding all LHC data

We have seen that the LHC data have a significant impact on various PDFs. Both in order to precisely gauge this impact, and in view of possible applications in which usage of PDFs without LHC data is required, we have produced a PDF set in which all LHC data are excluded from the NNPDF3.1 dataset. The distance between the ensuing PDF set and the default are shown in Fig. 47.

**Table 10:** The values of $\chi ^2/N_{\mathrm{dat}}$ for the deep-inelastic scattering experiments, as well as for the total dataset, for the NNPDF3.1 NNLO PDF set and for a new PDF determination which also includes the EMC charm structure function data

	NNPDF3.1 NNLO	+ EMC charm data
NMC	1.30	1.29
SLAC	0.75	0.76
BCDMS	1.21	1.24
CHORUS	1.11	1.10
NuTeV dimuon	0.82	0.88
HERA I+II inclusive	1.16	1.16
HERA $\sigma _c^\mathrm{NC}$	1.45	1.42
HERA $F_2^b$	1.11	1.11
EMC $F_2^c$	[4.8]	0.93
Total dataset	1.148	1.145

**Fig. 48:**
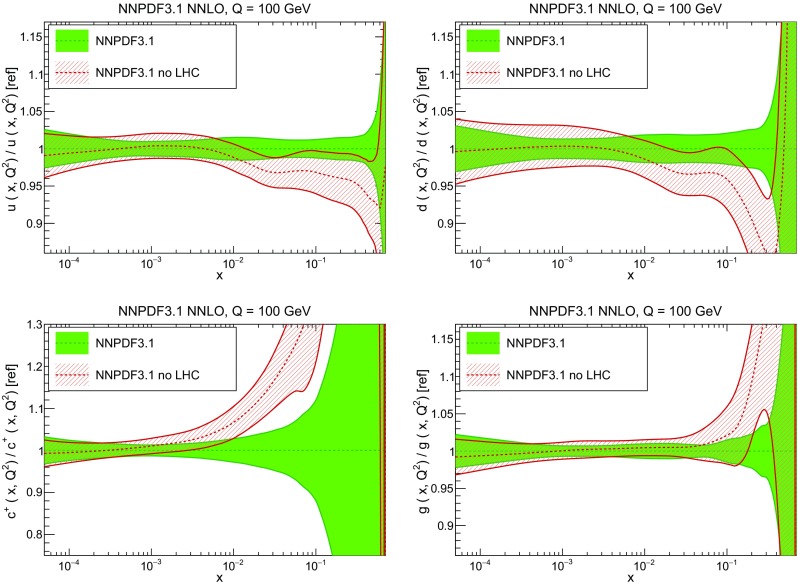
Same as Fig. 25 but now excluding all LHC data. Results are shown for the up (top left), down (top right), charm (bottom left) and gluon (bottom right) PDFs

The cumulative effect of the data which were discussed in Sects. 4.2–4.5 and 4.7 is considerable. Most PDFs are affected at the one-sigma level and in some cases (such as the down and charm quarks) at up to the two-sigma level. This is confirmed by direct comparison of the PDFs; see Fig. 48. The difference between the two fits appears to be mostly driven by the CHORUS, BCDMS and fixed-target Drell–Yan data, whose $\chi ^2$ improves, respectively, by 84, 32 and 38 units when removing the LHC data. Other datasets display much smaller differences, typically compatible with statistical fluctuations, and in some cases (such as for the SLAC data) the fit quality is actually somewhat better in the global fit.

On the other hand, it is clear that the shifts between PDFs without LHC data and those including them are compatible with the respective PDF uncertainties, and that the uncertainties on the PDFs determined without LHC data are not so large as to render them useless for phenomenology. We conclude that the default set remains considerably more accurate and should be used for precision phenomenology. However, the use of PDFs determined without some or all the LHC data may be mandatory in searches for new physics, in order to make sure that possible new physics effects are not reabsorbed in the PDFs. Under such circumstances, we conclude that even though the uncertainty in the PDFs without LHC data is not competitive, the level of deterioration is not so great as to make searches for new physics altogether impossible.

### Nuclear targets and nuclear corrections

The NNPDF3.1 dataset includes several measurements taken upon nuclear targets. DIS data from the SLAC, BCDMS and NMC experiments along with the E886 fixed-target Drell–Yan data involve measurements of deuterium. All neutrino data and the fixed-target E605 Drell–Yan data, are obtained with heavy nuclear targets. All of these data were already included in previous PDF determinations, including NNPDF3.0. The impact of nuclear corrections was studied in Ref. [5] and found to be under control. However, the much wider dataset might now permit the removal of these data from the global dataset: whereas removing data inevitably entails some loss of precision, this might be more than compensated by the increase in accuracy due to the complete elimination of any dependence on uncertain nuclear corrections.

**Fig. 49:**
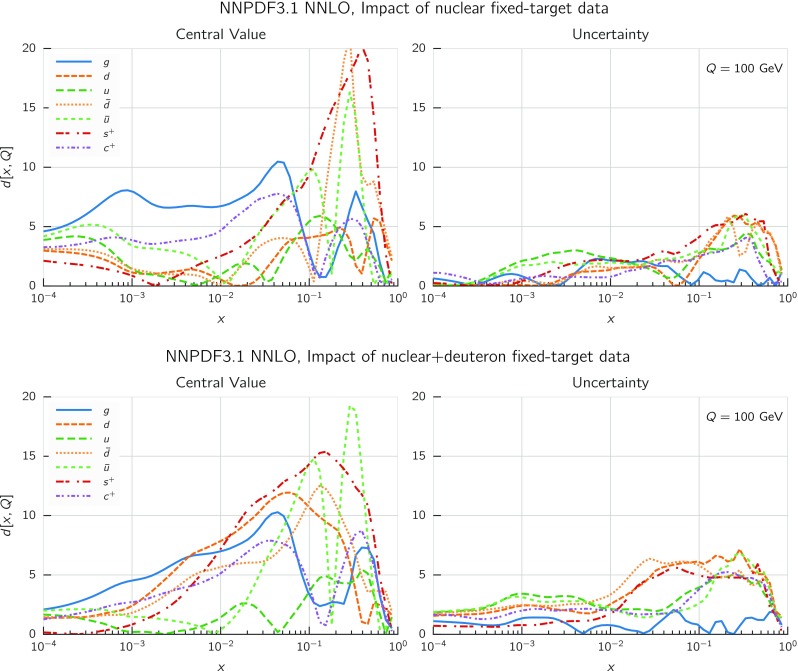
Same as Fig. 24 but now excluding all data with heavy nuclear targets, but keeping deuterium data (top) or excluding all data with any nuclear target and only keeping proton data (bottom)

In order to assess this, we performed two additional PDF determinations with the NNPDF3.1 methodology. Firstly, by removing all heavy nuclear target data but keeping deuterium data, and secondly removing all nuclear data and only keeping proton data. The distances between the default and these two PDF sets are shown in Fig. 49. At large *x* the impact of nuclear target data is significant, at the one to two-sigma level, mostly on the flavor separation of the sea. The deuterium data also have a significant impact, particularly in the intermediate *x* range.

**Fig. 50:**
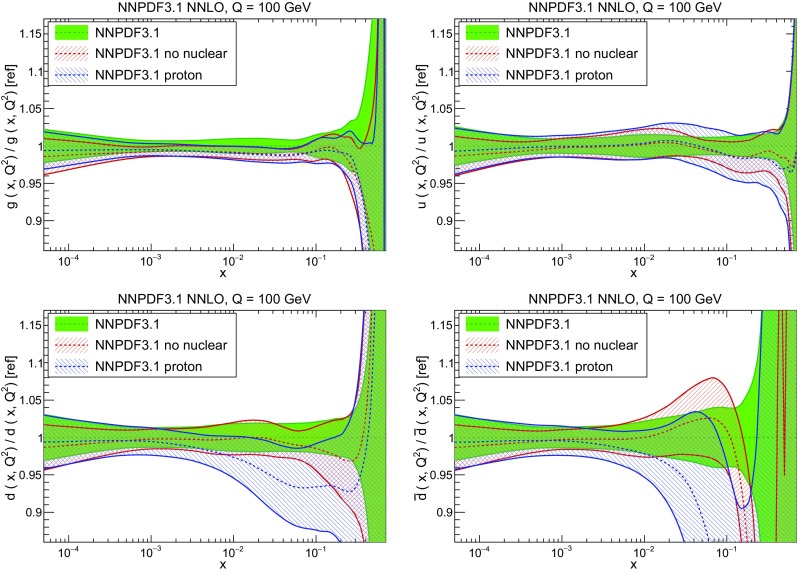
Same as Fig. 25 but now excluding all data with heavy nuclear targets, but keeping deuterium data, or excluding all data with any nuclear target and only keeping proton data. Results are shown for the gluon (top left), up (top right), down (bottom left) and antidown (bottom right)

**Fig. 51:**
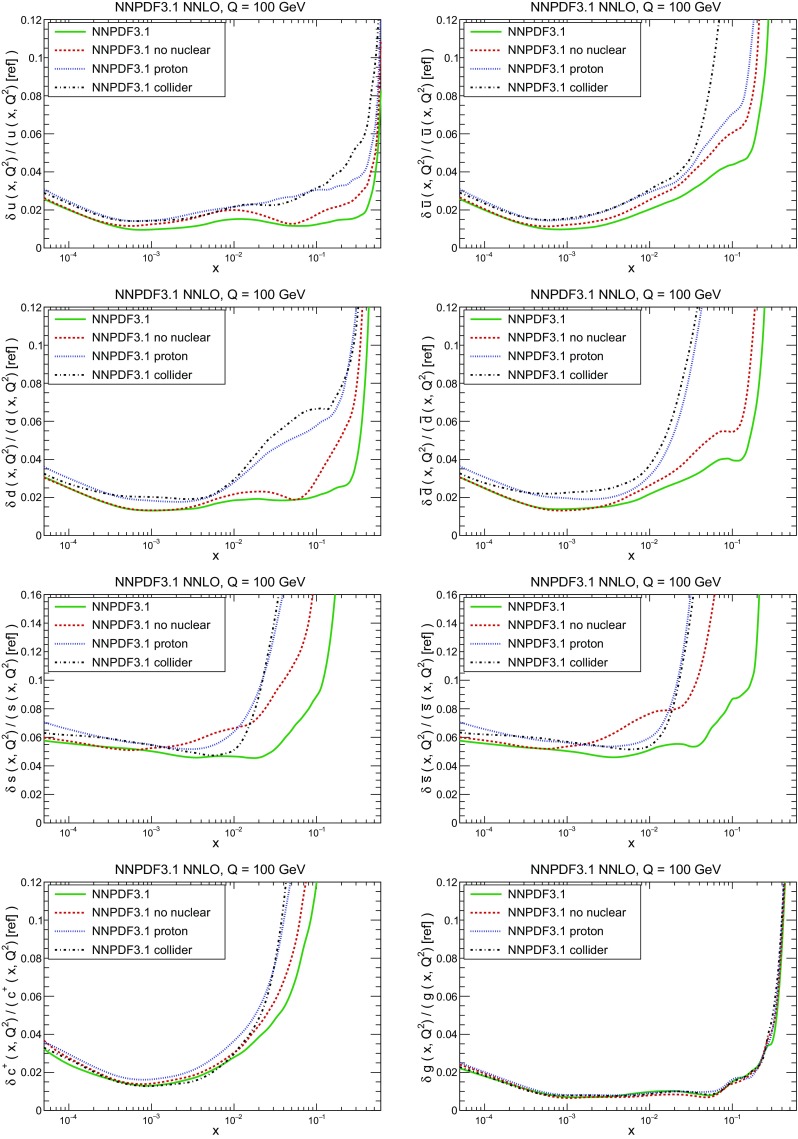
Comparison of the relative PDF uncertainties at $Q=100$ between the NNPDF3.1 and the no heavy nuclei, proton-only and collider-only PDF determinations. The uncertainties shown are all normalized to the NNPDF3.1 central value

A direct comparison of PDFs, in Fig. 50, and their uncertainties, in Fig. 51, shows that indeed PDFs determined with no heavy nuclear target data are reasonably compatible with the global set, though with rather larger uncertainties, especially for strangeness. Indeed, best-fit results without heavy nuclear targets, or even without deuterium data, are all compatible within their respective uncertainties, which is consistent with the previous conclusion that the absence of nuclear corrections for these data does not lead to significant bias at the level of current PDF uncertainties. On the other hand, PDFs determined with only proton data while compatible to within one sigma with the global set within their larger uncertainties, show a substantial loss of precision. This is particularly notable for down quarks, due to the importance of deuterium data in pinning down the isospin triplet PDF combinations.

Because deuterium data have a significant impact on the fit, one may worry that nuclear corrections to the deuterium data are now no longer negligible, at the accuracy of the present PDF determination. In order to investigate this issue in greater detail, we have performed a variant of the NNPDF3.1 NNLO default PDF determination in which all deuterium data are corrected using the same nuclear corrections as used by MMHT14 (specifically, Eqs. (9,10) of Ref. [7]).

**Fig. 52:**
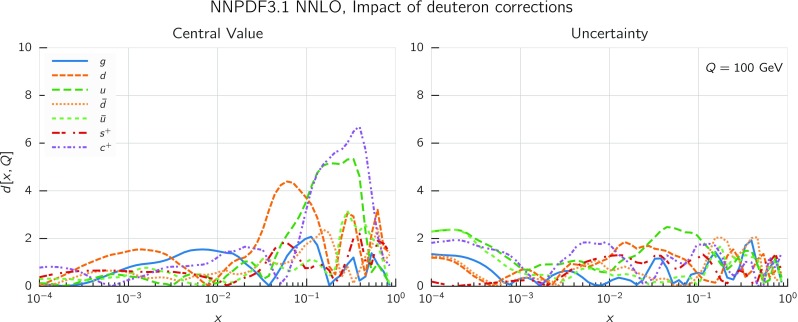
Same as Fig. 24 but now comparing the default NNPDF3.1 to a version in which all deuterium data have been corrected using the nuclear corrections from Ref. [7]

**Fig. 53:**
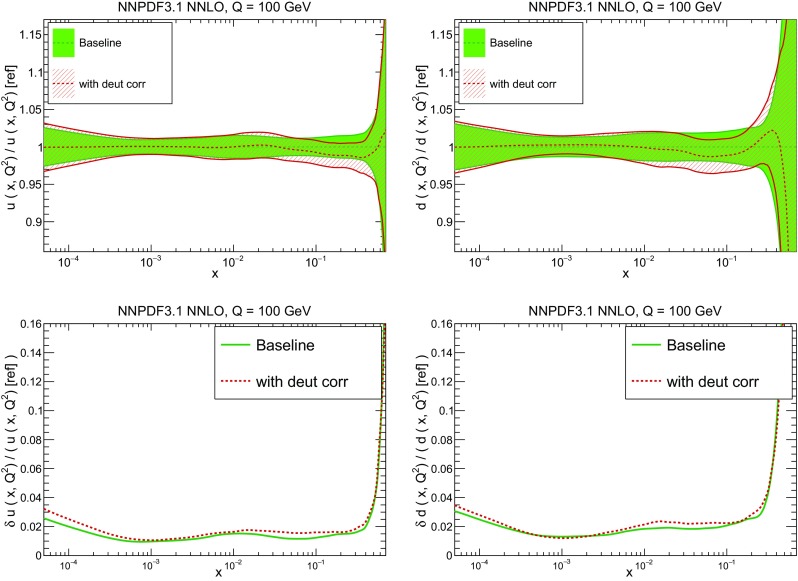
Same as Fig. 25 but now comparing the default NNPDF3.1 to a version in which all deuterium data have been corrected using the nuclear corrections from Ref. [7]. Results are shown for the up (left = and down (right) PDFs. The uncertainties are also shown (bottom row)

In terms of fit quality we find that the inclusion of nuclear corrections leads to a slight deterioration in the quality of the fit, with a value of $\chi ^2/N_{\mathrm{dat}}=1.156$, to be compared with the default $\chi ^2/N_{\mathrm{dat}}=1.148$ (see Table 6). In particular we find that for the NMC, SLAC, and BCDMS data the values of $\chi ^2/N_{\mathrm{dat}}$ with (without) nuclear corrections are, respectively, 0.94(0.95), 0.71(0.70), and 1.11(1.11). Therefore, the addition of deuterium corrections has no significant impact on the fit quality to these data.

The distances between PDFs determined including deuterium corrections and the default are shown in Fig. 52. They are seen to be moderate and always below the half-sigma level, and confined mostly to the up and down PDFs, as expected. These PDFs are shown in Fig. 53, which confirms the moderate effect of the deuterium correction. It should be noticed that the PDF uncertainty, also shown in Fig. 53, is somewhat increased when the deuterium corrections are included. The relative shifts for other PDFs are yet smaller since they are affected by larger uncertainties, which are also somewhat increased by the inclusion of the nuclear corrections.

In view of the theoretical uncertainty involved in estimating nuclear corrections, and bearing in mind that we see no evidence of an improvement in fit quality while we note a slight increase in PDF uncertainties when including deuterium corrections using the model of Ref. [7], we conclude that the impact of deuterium corrections on the NNPDF3.1 results is sufficiently small; they may be safely ignored even within the current high precision of PDF determination. Nevertheless, more detailed dedicated studies of nuclear corrections, also in relation to the construction of nuclear PDF sets, may well be worth pursuing in future studies.

In conclusion, for the time being it still appears advantageous to retain nuclear target data in the global dataset for general-purpose PDF determination. However, if very high accuracy is required (such as, for instance, in the determination of standard model parameters) it might be preferable to use PDF sets from which all data with nuclear targets have been omitted.

**Fig. 54:**
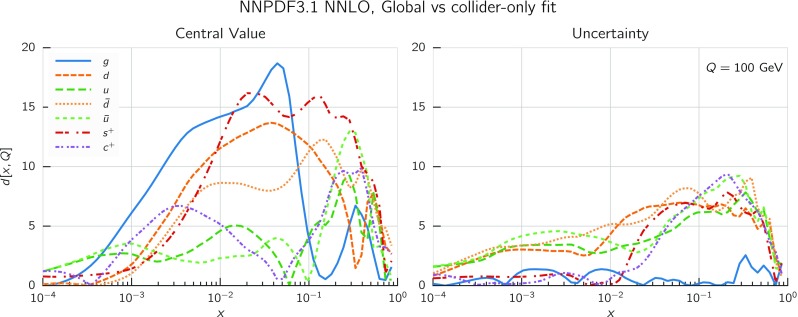
Same as Fig. 24 but now only keeping collider data

**Fig. 55:**
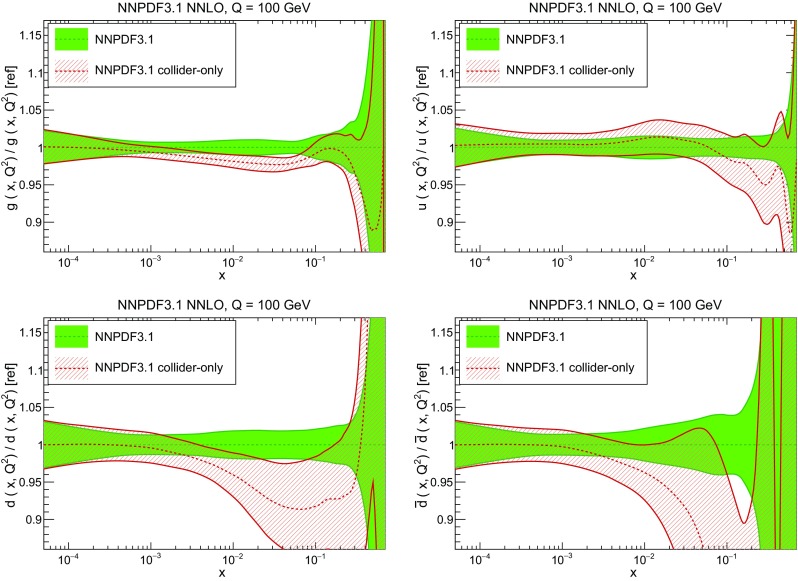
Same as Fig. 25 but now only keeping collider data. Results are shown for the gluon (top left), up (top right), down (bottom left) and antidown (bottom right)

### Collider-only parton distributions

A yet more conservative option to that discussed in the previous section is to retain only collider data from HERA, the Tevatron and the LHC. The motivation for this suggestion, first presented in the NNPDF2.3 study [133], is that this excludes data taken at low scales, which may be subject to potentially large perturbative and non-perturbative corrections. Furthermore, data taken on nuclear targets, and all of the older datasets are eliminated, thereby leading to a more reliable set of PDFs. However, previous collider-only PDFs had very large uncertainties, due to the limited collider dataset then available.

In order to re-assess the situation with the current, much wider LHC dataset, we have repeated a collider-only PDF determination. This amounts to repeating the proton-only PDF determination described in the previous section, but now with the proton fixed-target data also removed. The distances between the ensuing PDFs and the default NNPDF3.1 are shown in Fig. 54. Comparing to Fig. 49 we notice that distances are similar for most PDFs, the main exception being the gluon, which in the intermediate *x* region has now shifted by almost two sigma.

This is confirmed by a direct comparison of PDFs in Fig. 55 and their uncertainties in Fig. 51. The valence quarks (especially up) are reasonably stable, but the sea now is quite unstable upon removal of all the fixed-target data, visibly more than in the proton-only set Fig. 50, with in particular a substantial increase in the uncertainty of the anti-up quark distribution at large *x*. Furthermore, the gluon, which in Fig. 50 was quite stable in the proton-only PDF set, now undergoes a significant downward shift at intermediate *x*, even though its uncertainty is not substantially increased.

We conclude that, despite impressive improvements due to recent LHC measurements, a collider-only PDF determination is still not very useful for general phenomenological applications.

## Implications for phenomenology

We now present some initial studies of the phenomenological implications of NNPDF3.1 PDFs. Firstly, we summarize the status of PDF uncertainties building upon the discussion in the previous section; we discuss the status of PDF uncertainties, and then we focus on the strange and charm PDFs. We then discuss PDF luminosities which are the primary input to hadron collider processes, and predictions for LHC processes, specifically *W*, *Z* and Higgs production at the LHC. As elsewhere, only a selection of results is presented here, with a much larger set available online as discussed in Sect. 6.2.

### Improvements in PDF uncertainties

**Fig. 56:**
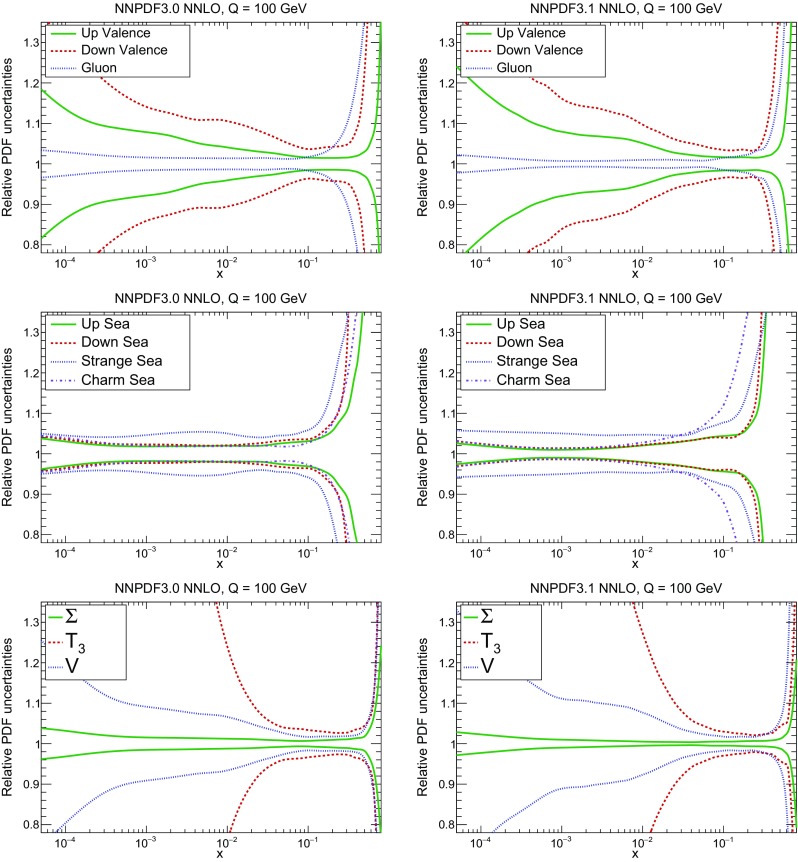
Comparison of relative uncertainties on NNPDF3.0 (left) and NNPDF3.1 (right) NNLO PDFs, normalized to the NNPDF3.1 NNLO central value. The two light-quark valence PDFs and the gluon are shown (top) along with all individual sea PDFs (center) and the singlet, valence and isospin triplet combinations (bottom)

After discussing in Sect. 4 the impact on NNPDF3.1 PDFs of each individual new piece of data and after separating off the effect of the new methodology, we can now study the combined effect of all the new data by comparing PDF uncertainties in NNPDF3.0 and NNPDF3.1. This is done in Fig. 56, where we compare relative PDF uncertainties (all computed with a common normalization) on PDFs in the NNPDF3.0 and NNPDF3.1 sets, shown as valence (i.e. $q-\bar{q}$), and sea (i.e. $\bar{q}$) for up and down and $q+\bar{q}$ for strange and charm. Results are shown both for individual PDF flavors, and for the singlet, valence, and triplet combinations defined in Eq. (3.4) of Ref. [23]).

The most visible effect is the very considerable reduction in gluon uncertainty, which is now at the percent level for almost all *x*. As discussed in Sect. 4, this is due to the combination of many mutually consistent constraints on the gluon from DIS (especially at HERA), *Z* transverse momentum distributions, jet production, and top-pair production, which taken together cover a very wide kinematic range. The singlet quark combination, which mixes with the gluon, shows a comparable improvement for all $x\lesssim 0.1$, but less marked at large *x*.

Interestingly, for quark PDFs the pattern of uncertainties is different in the flavor basis versus the “evolution” basis as given in Eq. (3.4) of Ref. [23]). Specifically, the aforementioned reduction in uncertainty on the singlet combination is not seen in any of the light-quark valence distributions, which generally have comparable uncertainties in NNPDF3.1 and NNPDF3.0. This is due to the fact that flavor separation is somewhat more uncertain in NNPDF3.1, due to the fact that charm is now independently parametrized. This is compensated by the availability of more experimental information (in particular LHCb and ATLAS data), but not at small and very large *x*. Indeed, the valence and triplet distributions have generally somewhat larger uncertainties in NNPDF3.1 than in NNPDF3.0, except for $x\sim 0.1$.

This pattern is also seen in sea uncertainties. At small $x\lesssim 10^{-2}$ they are all comparable, and all (except for strangeness) rather smaller than the corresponding uncertainties in NNPDF3.0, since they are driven by the mixing of the singlet and the gluon through perturbative evolution at small *x* [134]. Even the charm PDF, now independently parametrized, has a smaller uncertainty. In the large-*x* region, instead, the less accurate knowledge of flavor separation kicks in, and relative uncertainties are larger. Clearly, in NNPDF3.0 charm had an unnaturally small uncertainty, since at large *x* perturbatively generated charm is tied to the gluon. In NNPDF3.1, instead, the hierarchy of uncertainties on sea PDFs in the valence region is what one would expect, with up and down known most accurately, and strange and charm affected by increasingly large uncertainties. The uncertainty in NNPDF3.1 on the up and especially down sea components is a little increased, but still comparable to NNPDF3.0, while the uncertainty in strangeness is stable and that on charm significantly increased, as it should be given that large-*x* charm is largely unconstrained by data. In this respect, it is interesting to observe that a more accurate determination of charm and other sea PDFs at large *x* can be achieved through the inclusion of the EMC dataset as discussed in Sect. 4.9 above. Future LHC data on processes such as $Z+c$ production may confirm the reliability of the EMC dataset.

### The strange PDF

Whereas there is broad consensus on the size, i.e. the central value, of up and down PDFs, for which there is good agreement between existing determinations within their small uncertainties, the size of the strange PDF has been the object of some controversy, which we revisit here in view of NNPDF3.1 results. Specifically, the strange fraction of the proton quark sea, defined as

5.1 $$\begin{aligned} R_\mathrm{s} (x,Q^2)={{ s(x,Q^2)+\bar{s}(x,Q^2) }\over { \bar{u}(x,Q^2)+\bar{d}(x,Q^2) }} \, . \end{aligned}$$

and the corresponding ratio of momentum fractions

5.2 $$\begin{aligned} K_\mathrm{s} (Q^2)={{\int _0^1 dx\, x \left( s(x,Q^2)+\bar{s}(x,Q^2)\right) }\over { \int _0^1 dx\, x\left( \bar{u}(x,Q^2) + \bar{d}(x,Q^2)\right) }} \, , \end{aligned}$$

have been traditionally assumed to be significantly smaller than one, and in PDF sets produced before the strange PDF could be extracted from the data, such as e.g. NNPDF1.0 [97], it was often assumed that $R_\mathrm{s}\sim {{1}\over {2}}$, for all *x*, and thus also $K_\mathrm{s}\sim {{1}\over {2}}$. This level of strangeness suppression is indeed found in many recent global PDF sets, in which the strongest handle on the strange PDF is provided by deep-inelastic neutrino inclusive $F_2$ and charm $F_2^c$ (“dimuon”) data.

**Table 11:** The strangeness fraction $R_\mathrm{s}(x,Q)$ Eq. (5.1) at $x=0.023$, at a low scale and a high scale. We show results obtained using NNPDF3.0, and NNPDF3.1 baseline, collider-only and HERA+ATLAS *W*, *Z* sets, compared with the xFitter ATLAS value Ref. [72]

PDF set	$R_\mathrm{s}(0.023,1.38~{\mathrm {GeV}})$	$R_\mathrm{s}(0.023,M_{Z})$
NNPDF3.0	0.45 ± 0.09	0.71 ± 0.04
NNPDF3.1	0.59 ± 0.12	0.77 ± 0.05
NNPDF3.1 collider-only	0.82 ± 0.18	0.92 ± 0.09
NNPDF3.1 HERA + ATLAS *W*, *Z*	1.03 ± 0.38	1.05 ± 0.240
xFitter HERA + ATLAS *W*, *Z* (Ref. [72])	$1.13 \,{}^{+0.11}_{-0.11}$	–

**Fig. 57:**
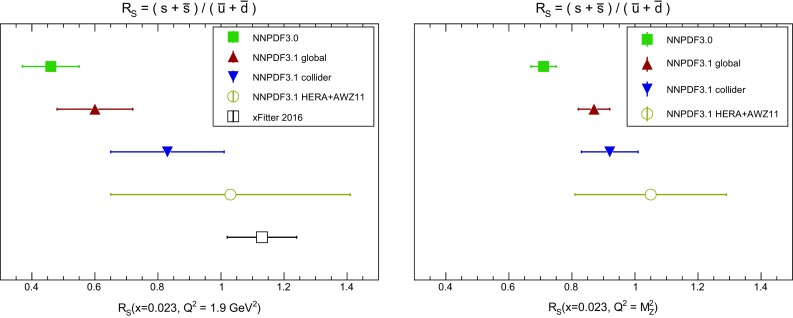
Graphical representation of the results of Table 11

This was challenged in Ref. [135] where, on the basis of ATLAS *W* and *Z* production data, combined with HERA DIS data, it was argued instead that, in the measured region, the strange fraction $R_\mathrm{s}$ is of order one. In Refs. [3, 133], respectively, based on the NNPDF2.3, and NNPDF3.0 global analyses, both of which include the data of Ref. [135], it was concluded that, whereas the ATLAS data do favor a larger strange PDF, they have a moderate impact on the global PDF determination, due to large uncertainties, and also that if the strange PDF is only determined from HERA and ATLAS data, the central value is consistent with the conclusion of Ref. [135], but the uncertainty is large enough to lead to agreement with the suppressed strangeness of the global PDF sets to within one sigma. In Ref. [3] it was also shown that the CMS $W+c$ production data [60], which were included there for the first time and which are also included in NNPDF3.1, though only in the NLO determination because of lack of knowledge of the NNLO corrections, have a negligible impact due to their large uncertainties.

As we discussed in Sect. 4.7, ATLAS *W* and *Z* production data have been supplemented by the rather more accurate dataset of Ref. [72], also claimed to favor enhanced strangeness. Indeed, we have seen in Sect. 4.7 that strangeness is significantly enhanced by the inclusion of these data and also, in Sect. 3.4, that this enhancement can be accommodated in the global PDF determination thanks to the independently parametrized charm PDF, which is a new feature to NNPDF3.1. It is thus interesting to re-assess strangeness in NNPDF3.1, by comparing theoretically motivated choices of dataset: we will thus compare with the previous NNPDF3.0 results for strangeness obtained using the default NNPDF3.1, the collider-only PDF set of Sect. 4.12, which can be considered to be theoretically more reliable, and a PDF set which we have constructed by using NNPDF3.1 methodology, but only including all HERA inclusive structure function data from Table 1 and the ATLAS data of Ref. [72]. Because inclusive DIS data alone cannot determine separately strangeness [1] this is then a determination of strangeness which fully relies on the ATLAS data.

In Table 11 we show NNLO results, obtained using these different PDF sets, for $R_\mathrm{s}(x,Q)$ Eq. 5.1 at $Q=1.38~\mathrm{GeV}$ (thus below charm threshold) and $Q=m_Z$ and $x=0.023$, an *x* value chosen by ATLAS in order to maximize sensitivity. Results are also compared with that of Ref. [72]. A graphical representation of the table is in Fig. 57.

First, comparison of the NNPDF3.1 HERA+ATLAS *W*, *Z* result with that of Ref. [72], based on the same data, shows agreement at the one-sigma level, with a similar central value and a greatly increased uncertainty, about four times larger, most likely because of the more flexible parametrization and because of independently parametrizing charm. Second, the strangeness in NNPDF3.1 is rather larger than in NNPDF3.0: as we have shown in Sects. 3.4 and 4.7, this is largely due to the effect of the ATLAS *W*, *Z* 2011 data, combined with determining charm from the data. Indeed, it is clear from Fig. 23 that the new data and new methodology both lead to strange enhancement, with the former effect dominant but the latter not negligible. This enhancement is more marked in the collider-only PDF set, which leads to a value which is very close to that coming from the ATLAS data. This suggests some tension between strangeness preferred by collider data and the rest of the dataset, i.e., most likely, neutrino data.

**Fig. 58:**
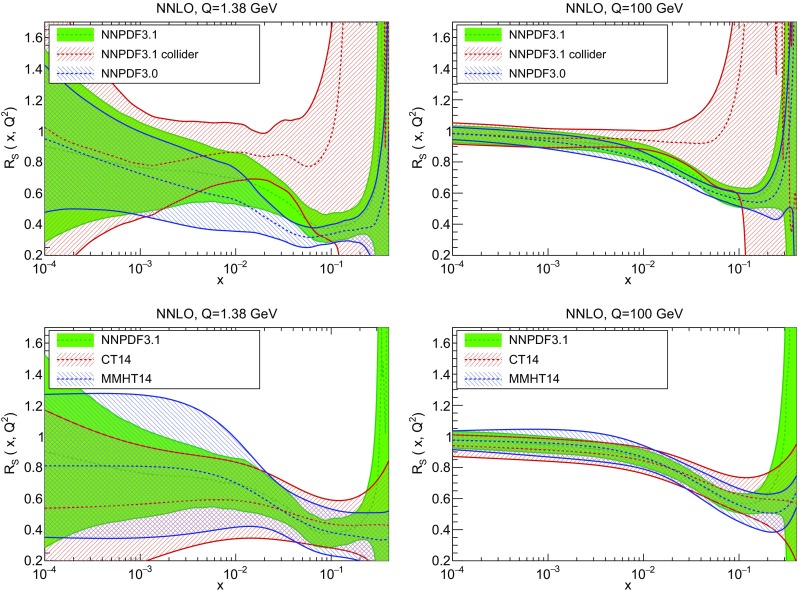
The strangeness ratio $R_\mathrm{s}(x,Q)$ Eq. (5.1) as a function of *x* for two values of *Q*, $Q=1.38$ GeV (left) and $Q=m_Z$ (right). Results are shown comparing NNPDF3.1 to NNPDF3.1 and the collider-only NNPDF3.1 (top), and to CT14 and MMHT (bottom)

It is interesting to repeat this analysis for the full *x* range. This is done in Fig. 58, where $R_\mathrm{s}(x,Q)$ Eq. (5.1) is plotted as a function of *x* again at low and high scales, now only including NNPDF3.0, and the default and collider-only versions of NNPDF3.1. It is clear that in the collider-only PDF set strangeness is largely unconstrained at large *x*, whereas the global fit is constrained by neutrino data to have a suppressed value $R_\mathrm{s}\sim 0.5$. At lower *x* we see the tension between this and the constraint from the collider data, which prefer a larger value.

In Fig. 58 we also compare the strangeness ratio $R_\mathrm{s}(x,Q)$ of NNPDF3.1 with that of CT14 and MMHT14. We find that there is good consistency in the entire range of *x*, while the PDF errors in NNPDF3.1 are typically smaller than those of the other two sets, especially at large scales. It is also interesting to note how in NNPDF3.1 the PDF uncertainties in the ratio $R_\mathrm{s}$ blow up at very large *x*, reflecting the lack of direct information on strangeness in that kinematic region.

**Table 12:** The strangeness momentum fraction Eq. (5.2) at a low scale and a high scale. We show results obtained using NNPDF3.0, and NNPDF3.1 baseline, collider-only and HERA+ATLAS *W*, *Z* PDF sets

PDF set	$K_\mathrm{s}(Q=1.38~{\mathrm {GeV}})$	$K_\mathrm{s}(Q=M_{\mathrm{Z}})$
NNPDF3.0	$0.45 \pm 0.07$	$0.72 \pm 0.04$
NNPDF3.1	$0.53 \pm 0.07$	$0.75 \pm 0.04$
NNPDF3.1 collider-only	$3.4 \pm 2.5$	$1.5 \pm 0.6$
NNPDF3.1 HERA + ATLAS *W*, *Z*	$-1.0 \pm 7.0$	$2.8 \pm 1.7$

We now turn to the strange momentum fraction $K_\mathrm{s}(Q^2)$ Eq. (5.2); values for the same PDF sets and scales are shown in Table 12. Results are quite similar to those found from the analysis of Table 11. For the NNPDF3.1 collider-only and especially the HERA + ATLAS *W*, *Z* fits, the central value of $K_\mathrm{s}$ is unphysical, with a huge uncertainty; essentially, all one can say is that the strange momentum fraction $K_\mathrm{s}$ is completely uncertain. This shows rather dramatically that the relatively precise values in Table 11 only hold in a rather narrow *x* range. It will be interesting to see whether more LHC data, possibly leading to a competitive collider-only fit, will confirm strangeness enhancement and allow for an accurate determination of strangeness in a wider range of *x*.

**Fig. 59:**
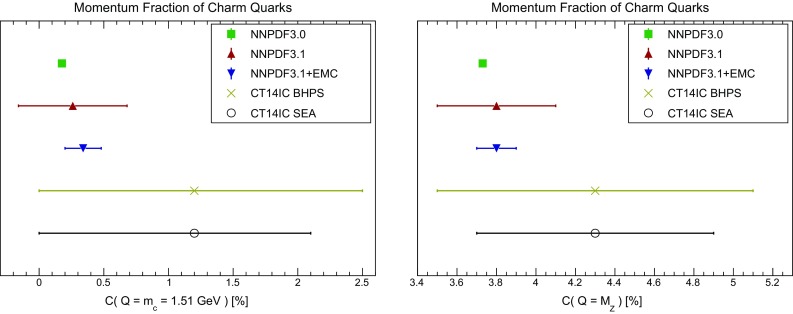
Graphical representation of the results for $C(Q^2)$ from Table 13 and $Q=m_\mathrm{c}$ GeV (left) and $Q=m_Z$ (right). Model estimates from Ref. [136] are also shown

### The charm content of the proton revisited

The charm content of the proton determined by fitting the charm PDF was quantified within the NNPDF global analysis framework in [23], where results obtained when charm is independently parametrized, or perturbatively generated, were compared for the first time. The analysis there was performed at NLO only, and the dataset was very similar to that of the NNPDF3.0 fit. We now re-examine the fitted charm PDF at NNLO in perturbative QCD, and in the context of the inclusion of the new datasets, in particular top, and LHCb and ATLAS electroweak boson production, which sizably affects and constrains the charm PDF.

Indeed, we have seen in Sect. 4.1, in particular Fig. 23, that the new data added in NNPDF3.1 considerably reduces the charm PDF uncertainty, but also affects its central value, which changes by more than one sigma at large *x*. Also, in Ref. [23] charm at large *x* was mostly constrained by the EMC data which we discussed in Sect. 4.9 and which are not included in the default NNPDF3.1 PDF determination. As we have seen in Sect. 4.9 these data still have a significant impact on charm, hence a re-assessment of charm determination is in order both when this dataset is included and when it is not. We therefore now compare results obtained using the default NNPDF3.1 NNLO set, the modified version of that in which charm is generated perturbatively as discussed in Sect. 3.4, and the modified set in which the EMC data are added to the NNPDF3.1 dataset, as discussed in Sect. 4.9.

**Table 13:** The charm momentum fraction $C(Q^2)$, Eq. (5.3), just above the charm threshold $Q=m_\mathrm{c}$ GeV and at $Q=m_Z$. Results are shown for NNPDF3.1, and its modified versions in which EMC data are added to the dataset, or charm is not fitted

PDF set	$C(m_\mathrm{c}) \%$	$C(m_Z)$ %
NNPDF3.1	$\left( 0.26 \pm 0.42\right)$	$\left( 3.8 \pm 0.3\right)$
NNPDF3.1 with pert. charm	$\left( 0.176 \pm 0.005\right)$	$\left( 3.73 \pm 0.02\right)$
NNPDF3.1 with EMC data	$\left( 0.34 \pm 0.14\right)$	$\left( 3.8 \pm 0.1\right)$

In Table 13 we show the charm momentum fraction, defined as

5.3 $$\begin{aligned} C(Q^2) \equiv \int _0^1\, \mathrm{d}x\,\left( xc(x,Q^2)+x\bar{c}(x,Q^2)\right) \ , \end{aligned}$$

for two values of $Q^2$, at the charm threshold $Q=m_\mathrm{c}$, and at $Q=m_Z$, computed from using these PDF sets. A graphical representation of the results from Table 13 is shown in Fig. 59. There is a very large difference, by two orders of magnitude, between the uncertainty on the momentum fraction, according to whether charm is independently parametrized, or perturbatively generated. However, the central values agree with each other within the large uncertainty determination when charm is parametrized, with the corresponding central value only slightly larger than that when charm is perturbative (though hugely different on the scale of the uncertainty on the perturbatively generated result). Upon adding the EMC data the uncertainty is reduced by about a factor of three, and the central value somewhat increased, consistently with the effect of this dataset on the charm PDF discussed in Sect. 4.9.

**Fig. 60:**
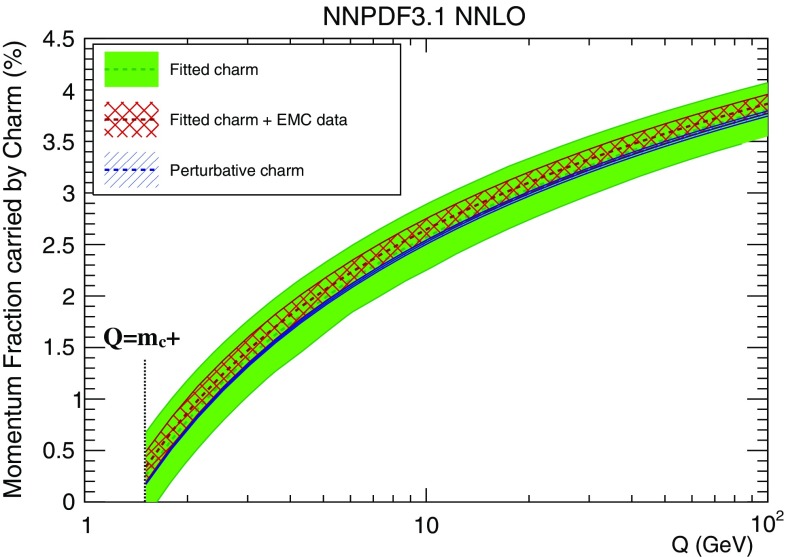
The charm momentum fraction of Table 13 plotted as a function of scale *Q*

**Fig. 61:**
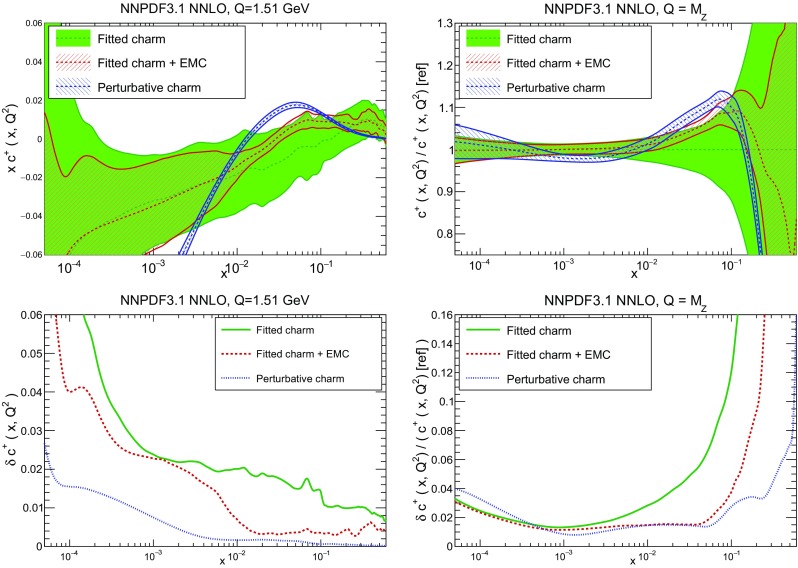
Comparison of the charm PDF at the scale and for the PDF sets of Table 13. Both the PDF (top) and the relative uncertainty (bottom) are shown

We can interpret the difference between the total momentum fraction when charm is independently parametrized and determined from the data, and that when charm is perturbatively generated, as the “intrinsic” (i.e. non-perturbative) charm momentum fraction. Including EMC data when charm is parametrized, at $Q=m_\mathrm{c}$ we find that it is given by $C(m_\mathrm{c})_{\mathrm{FC}}-C(m_\mathrm{c})_{\mathrm{PC}} = \left( 0.16 \pm 0.14\right)$%: this provides evidence for a small intrinsic charm component in the proton at the one-sigma level, somewhat improving the estimates of Ref. [23], but still with a considerable degree of uncertainty. Our estimate for the non-perturbative charm component of the proton is considerably smaller than those allowed in the CT14IC model analysis [136], also shown in Fig. 59. However, it is larger than expected in Ref. [137], where an upper bound of 0.5% at the four-sigma level is claimed. Both these analyses have difficulty fitting the EMC charm structure function data, due to an overly restrictive functional form for the charm PDF. At high scales all estimates of *C*(*Q*) are dominated by the perturbative growth of the charm PDF at small *x*, but the one-sigma excess in the fit with EMC data persists, though as an ever diminishing fraction of the whole. This is demonstrated very clearly in Fig. 60, where we plot the dependence of the charm momentum fraction $C(Q^2)$ on the scale *Q*.

**Fig. 62:**
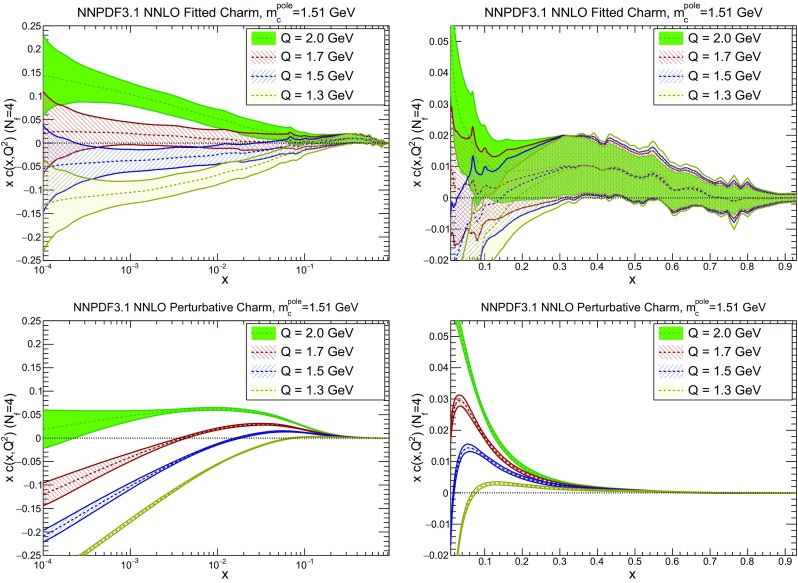
The charm PDF in the $n_f=4$ scheme at small *x* (left) and large *x* (right plot) for different values of *Q*, in the NNPDF3.1 NNLO PDF set (top) and when assuming that charm is perturbatively generated (bottom)

The origin of these values of the charm momentum fraction can be understood by directly comparing the charm PDF, which is done in Fig. 61, again just above threshold at $Q=m_\mathrm{c}$ and at $Q=m_Z$, in the latter case as a ratio to the baseline NNPDF3.1 result. The agreement of the charm momentum fraction when it is perturbatively generated or when it is parametrized and determined from the data is related to the fact that, when parametrized, the best-fit charm has qualitatively the same shape as charm generated perturbatively at NNLO, as observed in Ref. [23] and Sect. 3.4 above. However, at threshold $Q=m_\mathrm{c}$ the best-fit charm is larger than the perturbative component at large *x*, $x\gtrsim 0.2$, albeit with large uncertainties, that are somewhat reduced when the EMC dataset is added, without a significant change in shape. Upon addition of the EMC data, in the medium–small case, $10^{-2}\lesssim x\lesssim 10^{-1}$, the PDF is pushed at the upper edge of the uncertainty band before addition, with considerably reduced uncertainty. The unrealistically small uncertainty on the perturbatively generated charm PDF is apparent, and so is the reduction in uncertainty due to the EMC data for all $x\gtrsim 10^{-3}$, already discussed in Sect. 4.9.

The origin of the differences between the charm PDF when perturbatively generated, or parametrized and determined from the data, and a possible decomposition of the latter into an “intrinsic” (non-perturbative) and a perturbative component can be understood by studying their scale dependence close to threshold, in analogy to a similar analysis presented in Ref. [23]. This is done in Fig. 62, where the charm PDF is shown (in the $n_f=4$ scheme) as a function of *x* both when charm is parametrized and when it is perturbatively generated. On the one hand, the large $x\gtrsim 0.1$ component of the charm PDF is essentially scale independent: perturbative charm vanishes identically in this region, so the fitted result in this region may be interpreted as being of non-perturbative origin, i.e. “intrinsic”.

On the other hand, for smaller *x* the charm PDF depends strongly on scale. When perturbatively generated, it is sizable and positive already at $Q\sim 2$ GeV for all $x\gtrsim 10^{-3}$, while at threshold it becomes large and negative for all $x\lesssim 10^{-2}$, possibly because of large unresummed small-*x* contributions. The best-fit parametrized charm PDF, within its larger uncertainty, is rather flatter and smaller in modulus essentially for all $x\lesssim 10^{-1}$, except at the scale-dependent point at which perturbative charm changes sign. This difference in shape between fitted charm and perturbative charm for all $x\lesssim 0.1$ is rather larger than the uncertainty on either perturbative or fitted charm. This observation seems to support the conclusion that the assumption that charm is perturbatively generated might be a source of bias.

The updated NNPDF3.1 analysis is consistent with the conclusion of our earlier charm studies [23], namely that a non-perturbative charm component in the proton is consistent with global data, though the new high-precision collider data that we include now sets more stringent bounds on how large this component can be. In particular, once the EMC charm structure function dataset is added, we see from Table 13 that a non-perturbative charm momentum fraction of ${\simeq }0.5\%$ represents a deviation from our best-fit value of around two to three sigma. Thus models of intrinsic charm which carry as much as 1% of the proton’s momentum are strongly disfavored by currently available data.

### Parton luminosities

**Fig. 63:**
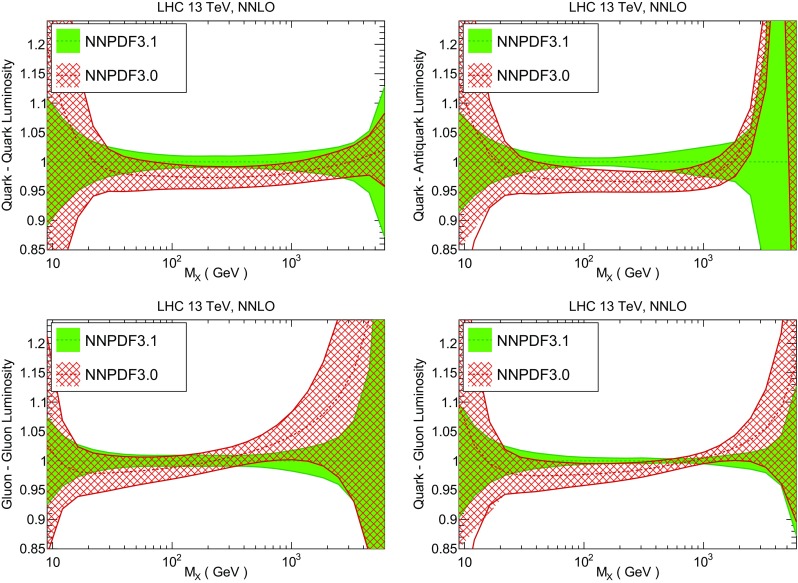
Comparison of parton luminosities with the NNPDF3.0 and NNPDF3.1 NNLO PDF sets for the LHC 13 TeV. From left to right and from top to bottom quark–antiquark, quark–quark, gluon–gluon and quark–gluon PDF luminosities are shown. Results are shown normalized to the central value of NNPDF3.1

**Fig. 64:**
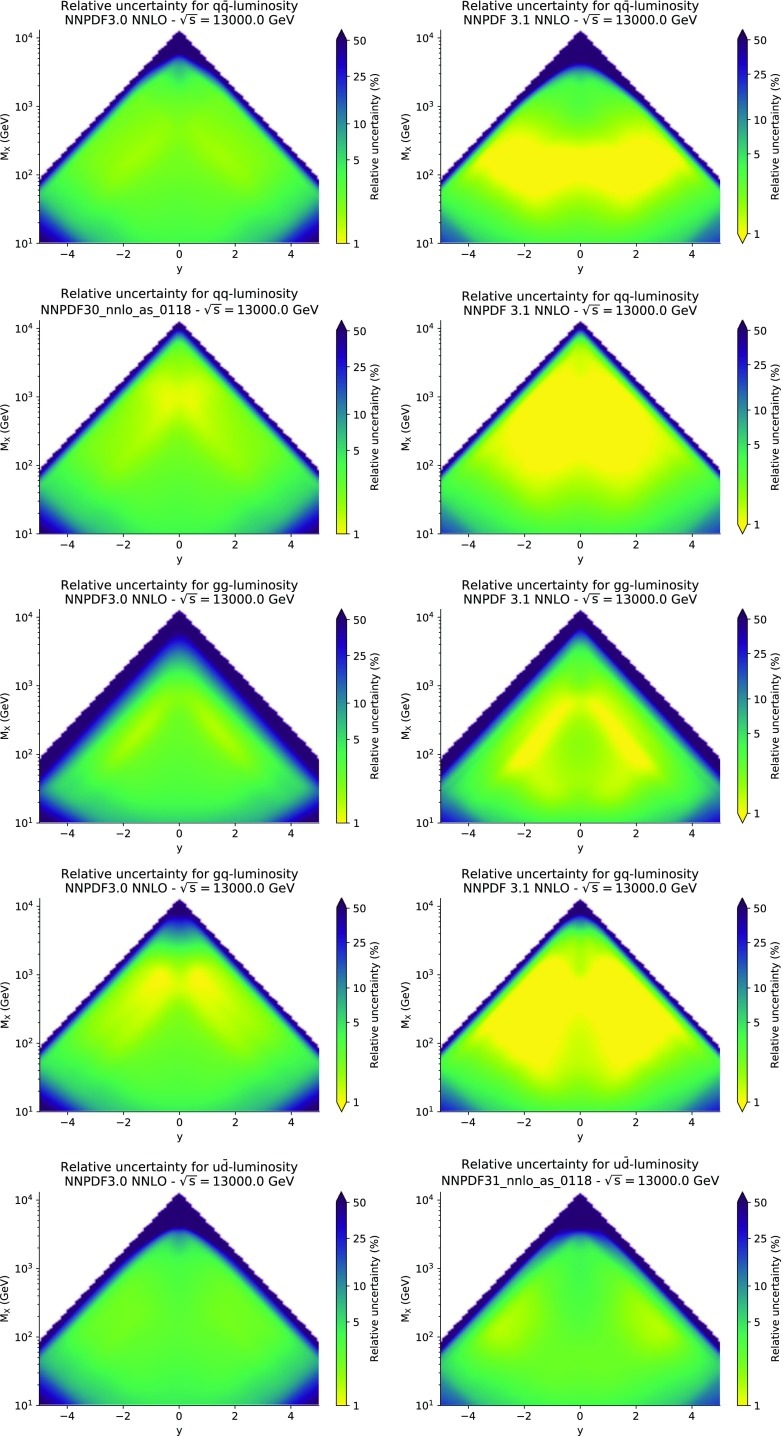
The relative uncertainty on the luminosities of Fig. 63, plotted as a function of the invariant mass $M_X$ and the rapidity *y* of the final state; the left plots show results for NNPDF3.0 and the right plots for NNPDF3.1 (upper four rows). The bottom row shows results for the up–antidown luminosity

After analytically discussing the phenomenological implication of individual PDFs and their uncertainties we now turn to parton luminosities (defined as in Ref. [138]) which drive hadron collider processes. Parton luminosities from the NNPDF3.0 and NNPDF3.1 NNLO sets for the LHC 13 TeV are compared in Fig. 63, and their uncertainties are displayed in Fig. 64 as a two-dimensional contour plot as a function of the invariant mass $M_y$ and rapidity *y* of the final state, all normalized to the NNPDF3.1 central value. We show results for the quark–quark, quark–antiquark, gluon–gluon and gluon–quark luminosities, relevant for the measurement of final states which do not couple to individual flavors (such as *Z* or Higgs). In the uncertainty plot we also show for reference the up–antidown luminosity, relevant e.g. for $W^+$ production.

Two features of this comparison are apparent. First, quark luminosities are generally larger for all invariant masses, while the gluon luminosity is somewhat enhanced for smaller invariant masses and somewhat suppressed for larger invariant masses in NNPDF3.1 in comparison to NNPDF3.0. The size of the shift in the quark sector is of order of one sigma or sometimes even larger, while for the gluon is generally rather below the one-sigma level. This of course reflects the pattern seen in Sect. 3.3 for PDFs; see in particular Fig. 10. Secondly, uncertainties are greatly reduced in NNPDF3.1 in comparison to NNPDF3.0. This reduction is impressive; it is apparent in the plots of Fig. 64, where it is clear that, while uncertainties were typically of order 5% in most of the phase space for NNPDF3.0, they are now of the order of 1–2% in a wide central rapidities range $|y|\lesssim 2$ and for final-state masses 100 GeV$\lesssim M_x\lesssim 1$ TeV. This is a direct consequence of the reduction in uncertainties on both the gluon and the quark singlet PDF discussed in Sect. 5.1 above. Indeed, luminosities which are sensitive to the flavor decomposition, such as the up–antidown luminosity, also shown in Fig. 64, do not display a significant reduction in uncertainties when going to NNPDF3.0 to NNPDF3.1.

**Fig. 65:**
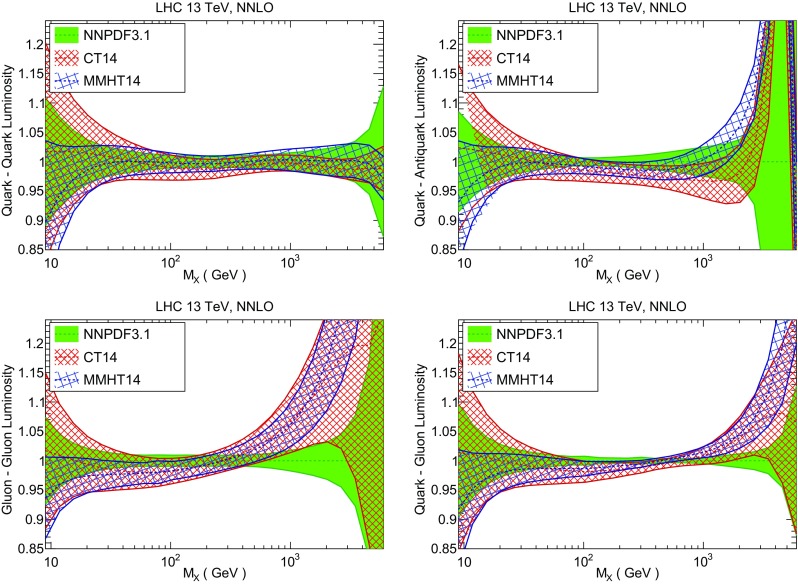
Same as Fig. 63, now comparing NNPDF3.1 NNLO to CT14 and MMHT14

We next compare NNPDF3.1 with CT14 and MMHT14: results are shown in Fig. 65. For the quark–quark luminosities, we find good agreement, while for quark–antiquark there is a somewhat bigger spread in central values though still agreement at the one-sigma level. Agreement becomes marginal at large masses, $M_X\gtrsim 2$ TeV, reflecting the limited knowledge of the large-*x* PDFs. For the gluon–gluon and gluon–quark channels we find reasonable agreement for masses up to $M_X\simeq 600$ GeV, relevant for precision physics at the LHC, but rather worse agreement for larger masses, relevant for BSM searches, in particular between NNPDF3.1 and MMHT14. Of course it should be kept in mind that NNPDF3.1 has a wider dataset and a larger number of independently parametrized PDFs than MMHT14 and CT14, hence the situation may change in the future once all global PDF sets are updated.

**Fig. 66:**
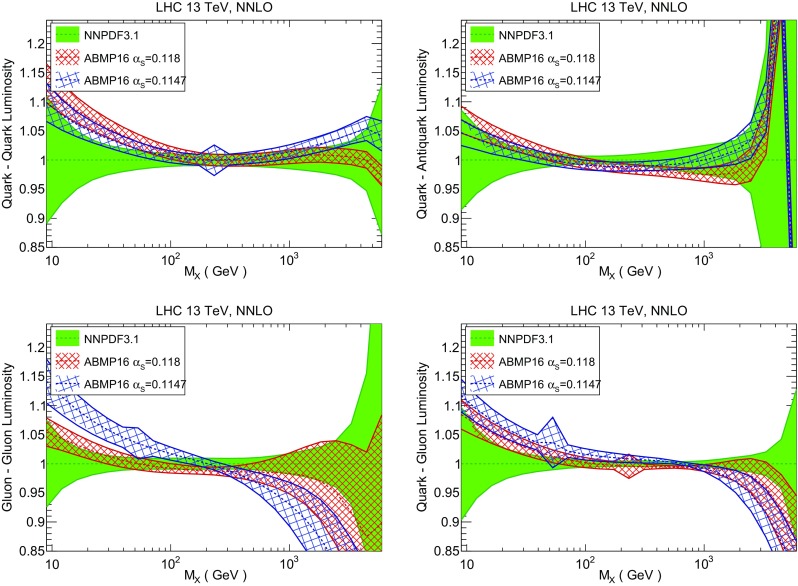
Same as Fig. 65, now comparing to ABMP16 PDFs, both with their default $\alpha _s(m_Z)=0.1149$ and with the common value $\alpha _s(m_Z)=0.118$

Next, in Fig. 66 we compare with ABMP16 PDFs. In this case, we show results corresponding both to the default ABMP16 set, which has $\alpha _s(m_Z)=0.1147$, and to the set with the common $\alpha _s(m_Z)=0.118$ adopted so far in all comparisons. While there are sizable differences between NNPDF3.1 and ABMP16 when the default ABMP16 value $\alpha _s(m_Z)=0.1147$ is used, especially for the gluon–gluon luminosity, the agreement improves when $\alpha _s(m_Z)=0.118$ is adopted also for ABMP16. However, ABMP16 luminosities have very small uncertainties at low and high $M_X$, presumably a consequence of an over-constrained parametrization, and of using a Hessian approach but with no tolerance, as discussed in Sect. 3.3.

**Fig. 67:**
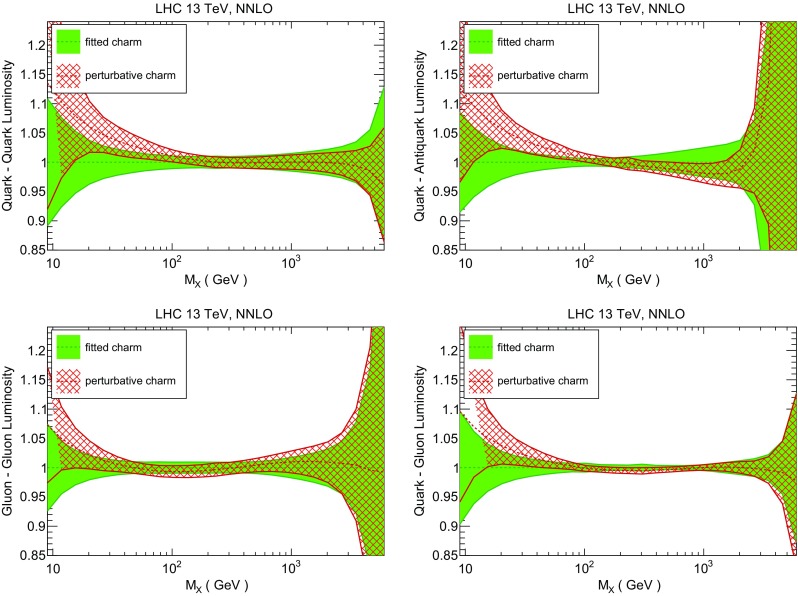
Same as Fig. 63, now comparing NNPDF3.1 to its modified version with perturbative charm

Finally, in order to further emphasize the phenomenological impact of the new NNPDF3.1 methodology, we compare NNPDF3.1 PDFs to the modified version in which the charm PDF is perturbatively generated, already discussed in Sects. 3.4, 5.3. Results are shown in Fig. 67. On the one hand, we confirm that despite having one more parametrized PDF, uncertainties are not increased. On the other hand, the effect on central values is moderate but non-negligible. For gluon–gluon and quark–gluon luminosities, differences are always below the one-sigma level, and typically rather less. For the quark–quark channel, results do not depend on the charm treatment for $M_X\gtrsim 200$ GeV, but for smaller invariant masses perturbatively generated charm leads to a larger PDF luminosity than the best-fit parametrized charm. For the quark–antiquark luminosity, we find a similar pattern at small $M_X$, but also some differences at medium and large $M_X$.

### *W* and *Z* production at the LHC 13 TeV

We compare theoretical predictions based on the NNPDF3.1 set to *W* and *Z* production data at $\sqrt{s}=13$ TeV from ATLAS [139]. Similar measurements by CMS [140] are not included in this comparison as they are still preliminary. We compute fiducial cross-sections using FEWZ [114] at NNLO QCD accuracy, using NNPDF3.1, NNPDF3.0, CT14, MMHT14 and ABMP16 PDFs, together with the corresponding PDF uncertainty band. All calculations (including ABMP16) are performed with $\alpha _s=0.118$.

Electroweak NLO corrections are computed with FEWZ for *Z* production, and with HORACE3.2 [141] for *W* production.

The fiducial phase space for the $W^{\pm }$ cross-section measurement in ATLAS is by $p_T^l\ge 25$ GeV and $|\eta _l|\le 2.5$ for the charged lepton transverse momentum and pseudo-rapidity, a missing energy of $p_T^\nu \ge 25$ GeV and a *W* transverse mass of $m_T\ge 50$ GeV.

For *Z* production, $p_T^l\ge 25$ GeV and $|\eta _l|\le 2.5$ for the charged leptons transverse momentum and rapidity and $66\le m_{ll} \le 116$ GeV for the dilepton invariant mass.

**Fig. 68:**
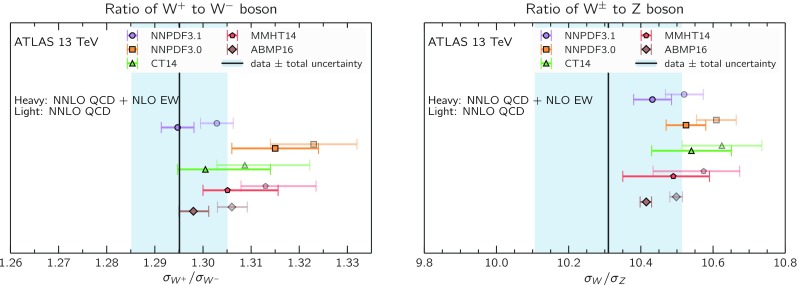
Comparison of the ATLAS measurements of the $W^+/W^-$ ratio (left) and the *W* / *Z* ratio (right) at $\sqrt{s}=13$ TeV with theoretical predictions computed with different NNLO PDF sets. Predictions are shown with (heavy) and without (light) NLO EW corrections computed with FEWZ and HORACE, as described in the text

In Fig. 68 we compare the ATLAS [139] 13 TeV measurements of the $W^+/W^-$ and *W* / *Z* ratios in the fiducial region at $\sqrt{s}=13$ TeV to theoretical predictions, both with and without electroweak corrections.

We see that, for both the $W^+/W^-$ ratio and the *W* / *Z* ratio, all the PDF sets are in reasonably good agreement with the data. The uncertainty in the theoretical prediction shown in the plot is the PDF uncertainty only: parametric uncertainties (in the values of $\alpha _s$ and $m_\mathrm{c}$) and missing higher-order QCD uncertainties are not included. Interestingly, electroweak corrections shift the theory predictions by around 0.5%, and for all PDF sets they improve the agreement with the ATLAS measurements. NNPDF3.1 results have smaller PDF uncertainties than NNPDF3.0, and are in better agreement with the ATLAS data.

**Fig. 69:**
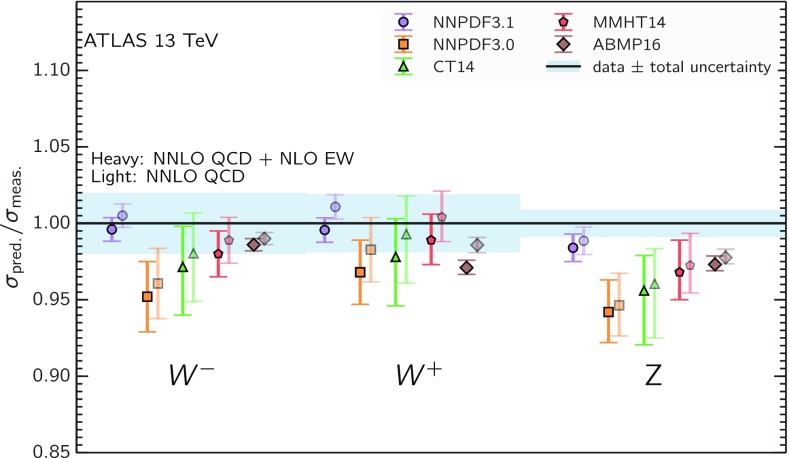
Same as Fig. 68, now for the absolute $W^+$, $W^-$ and *Z* cross-sections. All predictions are normalized to the experimental central value

The corresponding absolute $W^+$, $W^-$ and *Z* cross-sections are shown in Fig. 69, normalized in each case to the experimental central value. Again, predictions are generally in agreement with the data, with the possible exception of ABMP16 for *Z* production. In comparison to cross-section ratios, the effect of electroweak corrections on absolute cross-sections, around 1% for $W^+$ and $W^-$ and around 0.5% for *Z* production, is rather less significant on the scale of the uncertainties involved, and it does not necessarily lead to improved agreement.

Comparing NNPDF3.1 to NNPDF3.0 we see again considerably reduced uncertainties and improved agreement of the prediction with data. This improved agreement is particularly marked for *Z* production, where 3.0 was about 5% below the data, while now 3.1 agrees within uncertainties.

### Higgs production

We finally study the PDF dependence of predictions for inclusive Higgs production at LHC 13 TeV, and for Higgs pair production, which could also be within reach of the LHC in the near future [142, 143]. We study single Higgs in gluon fusion, associated production with gauge bosons and top pairs and vector boson fusion, and double Higgs production in gluon fusion. In each case, we show predictions normalized to the NNPDF3.1 result, and only show PDF uncertainties. All calculations (including ABMP16) are performed with $\alpha _s=0.118$.

The settings are the following. For gluon fusion we perform the calculation at N$^3$LO using ggHiggs [144–146]. Renormalization and factorization scales are set to $\mu _\mathrm{F}=\mu _\mathrm{R}=m_h/2$ and the computation is performed using rescaled effective theory. For associate production with a $t\bar{t}$ pair we use MadGraph5_aMC@NLO [147], with default factorization and renormalization scales $\mu _\mathrm{R}=\mu _\mathrm{F}=H_T/2$, where $H_T$ is the sum of the transverse masses. For associate production with an electroweak gauge boson we use the vh@nnlo code [148] at NNLO with default scale settings. For vector boson fusion we perform the calculation at N$^3$LO using proVBFH [149, 150] with the default scale settings. Finally, for double Higgs production at the FCC 100 TeV the calculation is performed using MadGraph_aMC@NLO.

**Fig. 70:**
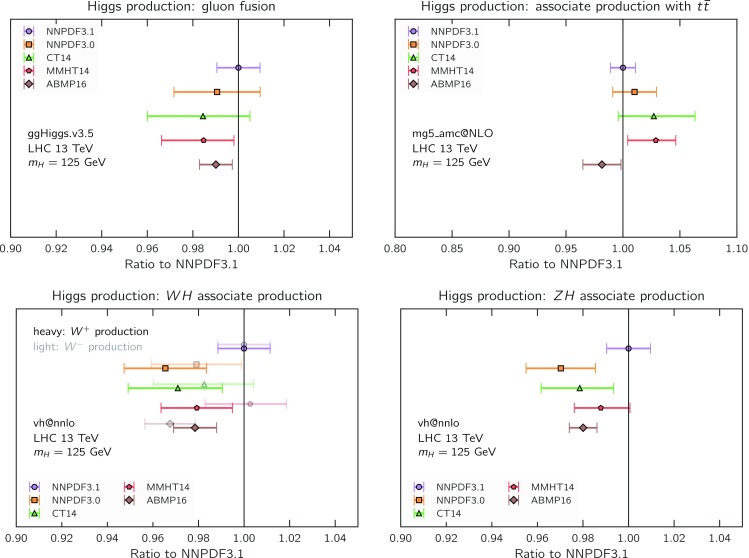
PDF dependence of the Higgs production cross-sections at the LHC 13 TeV for gluon fusion, $t\bar{t}$ associated production, and *VH* associated production. All results are shown as ratios to the central NNPDF3.1 result. Only PDF uncertainties are shown

**Fig. 71:**
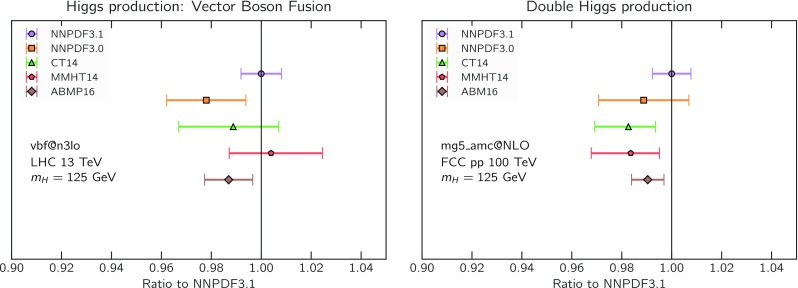
Same as Fig. 70 for single Higgs production in vector boson fusion (left) and double Higgs production in gluon fusion (right)

Results are shown in Figs. 70 and 71. For gluon fusion and $t\bar{t}h$, which are both driven by the gluon PDF, the former for $x\sim 10^{-2}$, and the latter for large *x*, results from the various PDF sets agree within uncertainties; NNPDF3.0 and NNPDF3.1 are also in good agreement, with the new prediction exhibiting reduced uncertainties. The spread of results is somewhat larger for associated production with gauge bosons. The NNPDF3.1 prediction is about 3% higher than the NNPDF3.0 one, with uncertainties reduced by a factor of 2, so the two cross-sections barely agree within uncertainties. Also, of the three PDF sets entering the PDF4LHC15 combination, NNPDF3.0 gave the smallest cross-section, but NNPDF3.1 now gives the highest one: *VH* production is driven by the quark–antiquark luminosity, and this enhancement for $M_X\simeq 200$ GeV between 3.0 and 3.1 could indeed be observed already in Fig. 63. For VBF we also find that the NNPDF3.1 result is larger, by about 2%, than the NNPDF3.0 one, with smaller uncertainties, and it is in better agreement with other PDF sets. Finally, for double Higgs production in gluon fusion the central value with NNPDF3.1 increases slightly but is otherwise consistent with the NNPDF3.0 prediction, and here there is also good agreement for all the PDF sets.

## Summary and outlook

NNPDF3.1 is the new main PDF release from the NNPDF family. It represents a significant improvement over NNPDF3.0, by including constraints from many new observables, some of which are included for the first time in a global PDF determination, thanks to the recent availability of the corresponding NNLO QCD corrections. Notable examples are $t\bar{t}$ differential distributions and the *Z* boson $p_T$ spectrum. From the theoretical point of view, the main improvement is to place the charm PDF on an equal footing as the light-quark PDFs. Independently parametrizing the charm PDF resolves a tension which would otherwise be present between ATLAS gauge boson production and HERA inclusive structure function data, leads to improved agreement with the LHC data, and turns the strong dependence of perturbatively generated charm on the value of the pole charm mass into a PDF uncertainty, as most of the mass dependence is reabsorbed into the initial PDF shape.

The NNPDF3.1 set is also the first set for which PDFs are delivered in a variety of formats: first of all, they are released both in Hessian and Monte Carlo form, and furthermore, the default sets are optimized and compressed so that a smaller number of Monte Carlo replicas or Hessian error sets reproduces the statistical features of much larger underlying replica sets. We now discuss how both Hessian and Monte Carlo reduced sets have been produced out of a large set of Monte Carlo replicas; we then summarize all PDF sets that have been made public through the LHAPDF interface; and finally we present a brief outlook on future developments.

### Validation of the NNPDF3.1 reduced sets

Default NNPDF3.1 NLO and NNLO PDFs for the central $\alpha _s(m_Z)=0.118$ value, as well as the modified version with perturbative charm discussed in Sect. 3.4, have been produced as $N_{\mathrm{rep}}=1000$ replica sets. These large replica samples have been subsequently processed using two reduction strategies: the Compressed Monte Carlo (CMC) algorithm [25], to obtain a Monte Carlo representation based on a smaller number of replicas, and the MC2H algorithm [24], to achieve an optimal Hessian representation of the underlying PDF probability distribution with a fixed number of error sets. Specifically, we have thus constructed CMC-PDF sets with $N_{\mathrm{rep}}=100$ replicas and MC2H sets with $N_{\mathrm{eig}}=100$ (symmetric) eigenvectors.

**Fig. 72:**
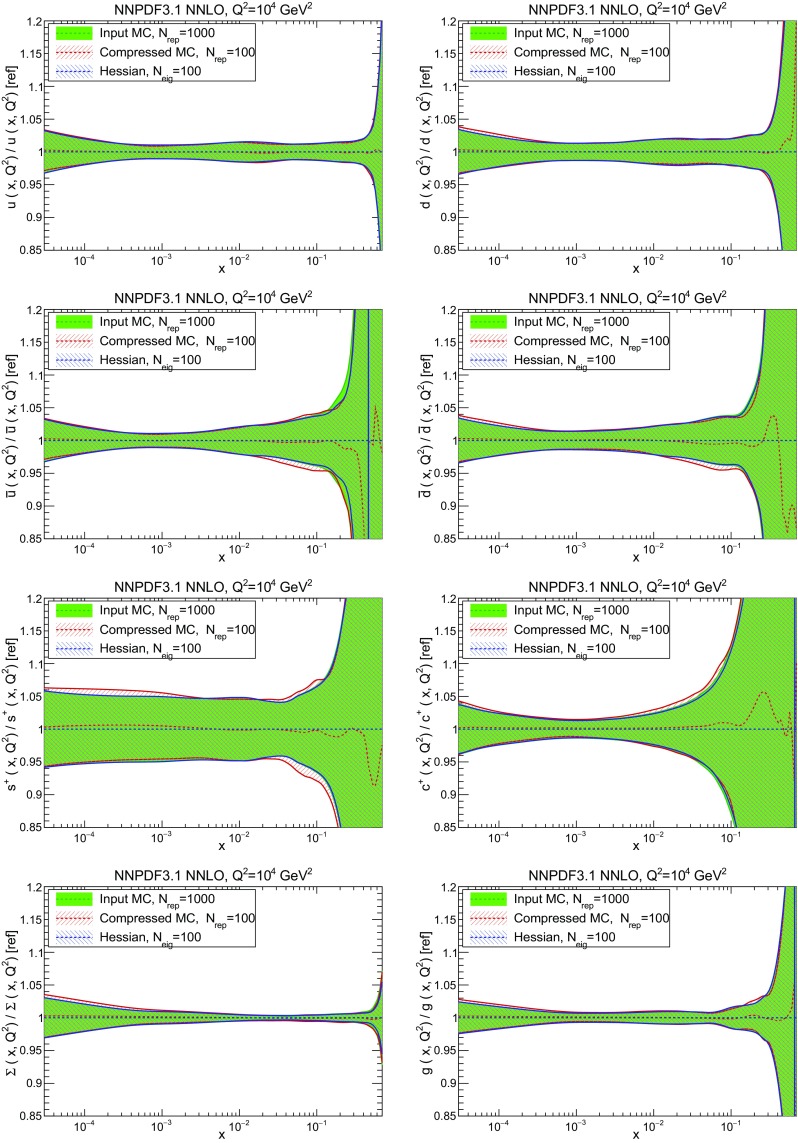
Comparison between the PDFs from the input set of $N_{\mathrm{rep}}=1000$ replicas of NNPDF3.1 NNLO, the reduced Monte Carlo CMC-PDFs with $N_{\mathrm{rep}}=100$ replicas, and the MC2H hessian PDFs with $N_{\mathrm{eig}}=100$ symmetric eigenvalues

In Fig. 72 we show the comparison between the PDFs from the input set of $N_{\mathrm{rep}}=1000$ replicas of NNPDF3.1 NNLO with the corresponding reduced sets of the CMC-PDFs with $N_{\mathrm{rep}}=100$ replicas and the MC2H hessian PDFs with $N_{\mathrm{eig}}=100$ eigenvalues. The agreement between the input $N_{\mathrm{rep}}=1000$ replica MC PDFs and the two reduced sets is very good in all cases. By construction, the agreement in central values and PDF variances is slightly better for the MC2H sets, since the CMC-PDF sets aim to reproduce also higher moments in the probability distribution and thus possibly non-gaussian features, while Hessian sets are Gaussian by construction.

Following the analysis of [24, 25] we have verified that also the correlations between PDFs are reproduced to a high degree of accuracy.

**Fig. 73:**
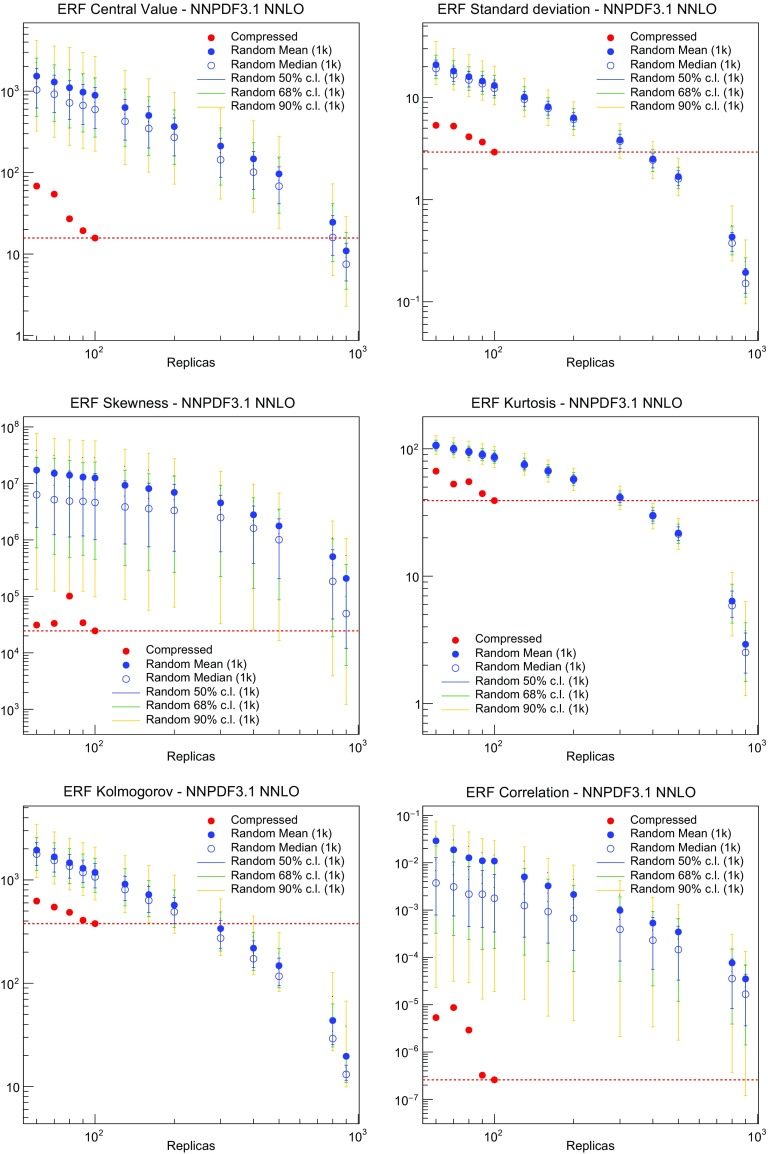
Comparison of estimators of the probability distributions computed using the NNPDF3.1 NNLO input $N_\mathrm{rep}=1000$ replica set, and compressed sets of $\widetilde{N}_\mathrm{rep}$ replicas, plotted as a function of $\widetilde{N}_{\mathrm{rep}}$. The error function (ERF) corresponding to central values, standard deviations, kurtosis, skewness, correlations and Kolmogorov distance are shown

In order to validate the efficiency of the CMC-PDF algorithm reduction from the starting $N_{\mathrm{rep}}=1000$ replicas down to the compressed $N_{\mathrm{rep}}=100$ replicas, in Fig. 73 we show, following the procedure described in [25], the summary of statistical estimators that compare specific properties of the probability distributions defined by the input $N_{\mathrm{rep}}=1000$ replicas of NNPDF3.1 NNLO and the corresponding compressed sets as a function of $\widetilde{N}_{\mathrm{rep}}$, the number of replicas in the reduced set starting from $\widetilde{N}_{\mathrm{rep}}=100$.

We compare the results of the compression algorithm with those of random selection of $\widetilde{N}_{\mathrm{rep}}$ replicas out of the original 1000 ones: the error function ERF corresponding to central values, standard deviations, kurtosis and skewness, correlations and the Kolmogorov distance are all shown. These results indicate that a CMC-PDF 100 replica set reproduces roughly the information contained in a random $\widetilde{N}_{\mathrm{rep}}=400$ PDF set.

### Delivery

We now provide a full list of the NNPDF3.1 PDF sets that are being made publicly available via the LHAPDF6 interface [151],

http://lhapdf.hepforge.org/.

As repeatedly mentioned in the paper, a very wide set of results concerning these PDF sets is available from the repository

http://nnpdf.hepforge.org/html/nnpdf31/catalog.

All sets are made available as $N_{\mathrm{rep}}=100$ Monte Carlo sets. For the baseline sets, these are constructed out of larger $N_{\mathrm{rep}}=1000$ replica sets, which are also being made available. The baseline sets are also provided as Hessian sets with 100 error sets.

The full list is the following:

- *Baseline NLO and NNLO NNPDF3.1 sets*
  Baseline NLO and NNLO NNPDF3.1 sets are based on the global dataset, with $\alpha _s(m_Z)=0.118$ and a variable-flavor number with up to five active flavors. These sets contain $N_{\mathrm{rep}}=1000$ PDF replicas. NNPDF31_nlo_as_0118_1000,
NNPDF31_nnlo_as_0118_1000.
  A modified version in which charm is perturbatively generated (ad in previous NNPDF sets) is also being made available, also with $N_{\mathrm{rep}}=1000$ PDF replicas: NNPDF31_nlo_pch_as_0118_1000,
NNPDF31_nnlo_pch_as_0118_1000.
  Out of these, optimized Monte Carlo $N_{\mathrm{rep}}=100$ NNPDF31_nlo_as_0118,
NNPDF31_nnlo_as_0118,
NNPDF31_nlo_pch_as_0118,
NNPDF31_nnlo_pch_as_0118, and Hessian sets with $N_{\mathrm{eig}}=100$ eigenvectors NNPDF31_nlo_as_0118_hessian,
NNPDF31_nnlo_as_0118_hessian,
NNPDF31_nlo_pch_as_0118_hessian,
NNPDF31_nnlo_pch_as_0118_hessian, have been constructed as discussed in Sect. 6.1 above. Out of these, smaller sets of eigenvectors optimized for the computation of specific observables may be constructed using the SM-PDF tool [26]. Specifically, sets optimized for a wide list of pre-defined observables can be generated and downloaded using the web interface [27] at https://smpdf.mi.infn.it.
- *Flavor number variation*
  We have produced sets, both at NLO and NNLO in which the maximum number of flavors differs from the default five, and it is either extended up to six, or frozen at four: NNPDF31_nlo_as_0118_nf_4,
NNPDF31_nlo_as_0118_nf_6,
NNPDF31_nnlo_as_0118_nf_4,
NNPDF31_nnlo_as_0118_nf_6.
  The variant with perturbatively generated charm is also made available, in this case also in a $n_f=3$ fixed-flavor number scheme: NNPDF31_nlo_pch_as_0118_nf_3,
NNPDF31_nlo_pch_as_0118_nf_4,
NNPDF31_nlo_pch_as_0118_nf_6,
NNPDF31_nnlo_pch_as_0118_nf_3,
NNPDF31_nnlo_pch_as_0118_nf_4,
NNPDF31_nnlo_pch_as_0118_nf_6.
- $\alpha _s$ *variation.* We have produced NNLO sets with the following values of $\alpha _s(m_Z)$: 0.108, 1.110, 0.112, 0.114, 0.116, 0.117, 0.118, 0.119, 0.120, 0.122, 0.124. Thus NNPDF31_nnlo_as_0108,
NNPDF31_nnlo_as_0110,
NNPDF31_nnlo_as_0112,
NNPDF31_nnlo_as_0114,
NNPDF31_nnlo_as_0116,
NNPDF31_nnlo_as_0117,
NNPDF31_nnlo_as_0118,
NNPDF31_nnlo_as_0119,
NNPDF31_nnlo_as_0120,
NNPDF31_nnlo_as_0122,
NNPDF31_nnlo_as_0124.
  For the values $\alpha _s(m_Z)=0.116$ and $\alpha _s(m_Z)=0.120$ we have also produced NLO sets, NNPDF31_nlo_as_0116,
NNPDF31_nlo_as_0120,
  and the variant with perturbative charm both at NLO and NNLO NNPDF31_nlo_pch_as_0116,
NNPDF31_nlo_pch_as_0120,
NNPDF31_nnlo_pch_as_0116,
NNPDF31_nnlo_pch_as_0120.
  In order to facilitate the computation of the combined PDF+$\alpha _s$ uncertainties we have also provided bundled PDF+$\alpha _s$ variation sets for $\alpha _s(m_Z)=0.118\pm 0.002$. These are provided both as Monte Carlo sets, and as Hessian sets. NNPDF31_nlo_pdfas,
NNPDF31_nnlo_pdfas,
NNPDF31_nlo_pch_pdfas,
NNPDF31_nnlo_pch_pdfas,
NNPDF31_nlo_hessian_pdfas,
NNPDF31_nnlo_hessian_pdfas,
NNPDF31_nlo_pch_hessian_pdfas,
NNPDF31_nnlo_pch_hessian_pdfas.
  They are constructed as follows:
  1. The central value (PDF member 0) is the central value of the corresponding $\alpha _s(m_Z)=0.118$ set.
  2. The PDF members 1 to 100 correspond to the $N_{{\mathrm {rep}}}=100$ ($N_{\mathrm{eig}}=100$) Monte Carlo replicas (Hessian eigenvectors) from the $\alpha _s(m_Z)=0.118$ set.
  3. The PDF members 101 and 102 are the central values of the sets with $\alpha _s(m_Z)=0.116$ and $\alpha _s(m_Z)=0.120$, respectively.
  Note that, therefore, in the Hessian case member 0 is the central set, and all remaining bundled members 1–102 are error sets, while in the Monte Carlo case, members 1–100 are Monte Carlo replicas, while members 0, 101 and 102 are central sets (replica averages). The way they should be used to compute combined PDF+$\alpha _s$ uncertainties is discussed e.g. in Ref. [12].
- *Charm mass variation* We provide sets with different values of the charm mass $m_\mathrm{c}^{\mathrm{pole}}$. They are available only at NNLO with $m_\mathrm{c}^{\mathrm{pole}}=1.38$ GeV and $m_\mathrm{c}^{\mathrm{pole}}=1.64$ GeV. NNPDF31_nnlo_as_0118_mc_138,
NNPDF31_nnlo_as_0118_mc_164.
  For comparison, the corresponding modified version with perturbative charm are also made available: NNPDF31_nnlo_pch_as_0118_mc_138,
NNPDF31_nnlo_pch_as_0118_mc_164.
- *Forced positivity sets*
  We provide sets in which PDFs are non-negative: NNPDF31_nlo_as_0118_mc,
NNPDF31_nlo_pch_as_0118_mc,
NNPDF31_nnlo_as_0118_mc,
NNPDF31_nnlo_pch_as_0118_mc.
  These have been constructed simply setting to zero PDFs whenever they become negative. They are thus an approximation, provided for convenience for use in conjunction with codes which fail when PDFs are negative.
- *LO sets* Leading-order PDF sets are made available $\alpha _s = 0.118$ and $\alpha _s = 0.130$: NNPDF31_lo_as_0118,
NNPDF31_lo_as_0130.
  The corresponding variants with perturbative charm are also provided: NNPDF31_lo_pch_as_0118,
NNPDF31_lo_pch_as_0130.
- *Reduced datasets* PDFs determined from subsets of the full NNPDF3.1, discussed in Sects. 4.2–4.12 are also made available, specifically

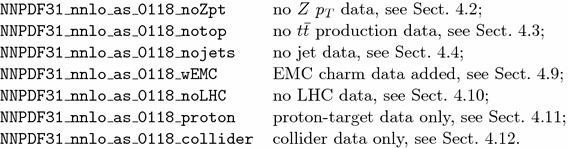

In addition to these, any other PDF sets discussed in this paper is also available upon request.

### Outlook

The NNPDF3.1 PDF determination presented here is an update of the previous NNPDF3.0, yet it contains substantial innovations both in terms of methodology and dataset and it leads to substantially more precise and accurate PDF sets. Thanks to this, several spin-offs can be pursued, either with the goal of updating existing results based on previous NNPDF releases, or in some cases because the greater accuracy enables projects which previously were either impossible or uninteresting.

These spin-off projects include the following:

- A precision determination of the strong coupling constant $\alpha _s(m_Z)$. We expect a significant increase in both precision and accuracy compared with previous NNPDF determinations [152, 153]. Specifically, thanks to the inclusion of many collider observables at NNLO with direct dependence on both the gluon and $\alpha _s(m_Z)$ it might turn out to be advantageous to drop altogether data taken on nuclear targets, and at low scales, i.e. base the $\alpha _s$ determination on the collider-only dataset of Sect. 4.12, or possibly an even more conservative dataset.
- A determination of the charm mass $m_\mathrm{c}$. This would be for the first time based on a PDF determination in which the charm PDF is independently parametrized, thereby avoiding bias related to the identification of $m_\mathrm{c}$ as a parameter which determines the size of the charm PDF.
- A NNPDF3.1QED PDF set including the photon PDF $\gamma (x,Q^2)$, thereby updating the previous NNPDF2.3QED [154] and NNPDF3.0QED [155] sets. This update should include recent theoretical progress: specifically the direct “LuxQED” constraints which determine the photon PDF from nucleon structure functions [156]; and NLO QED corrections to PDF evolution and DIS coefficient functions, now included in APFEL [99].
- PDF sets including small-*x* resummation, based on the formalism developed in [157–159], which has been implemented in the HELL code [160] and interfaced to APFEL. These would provide an answer the long-standing issue of whether or not small-*x* inclusive HERA data are adequately described by fixed-order perturbative evolution [161, 162], and may ultimately give better control of theoretical uncertainties at small *x*.

On a longer timescale, further substantial improvements in dataset and methodology are expected. On the one hand, so far we have essentially restricted ourselves to LHC 8 TeV data. A future release will include a significant number of 13 TeV measurements, of which several, from ATLAS, CMS and LHCb are already available. Specifically, the inclusion of more processes is envisaged, which are not currently part of the dataset, which have a large potential impact on PDFs, and for which higher-order corrections have become available. These include prompt photon production [163], for which NNLO corrections are now available [164] and whose impact on PDF is well known [165]; single-top production (also known at NNLO [166]); and possibly forward *D* meson production or more in general processes with final-state *D* mesons, such as $W+D$ production, recently measured by ATLAS [167], a process whose impact on PDFs has been repeatedly emphasized [168–170].

**Fig. 74:**
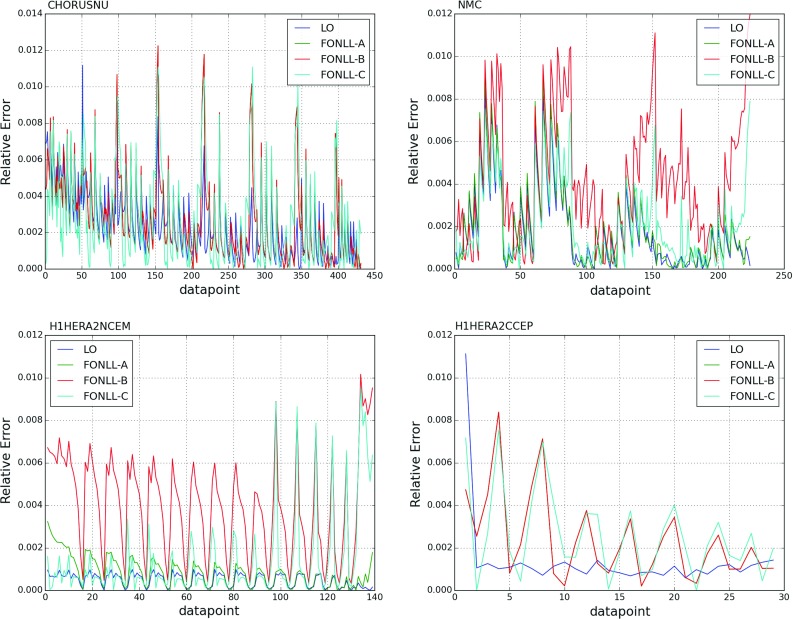
Relative difference in the DIS structure functions computed with FKgenerator and APFEL, using NNPDF3.0 as input PDF set, for the CHORUS charged-current neutrino–nucleus reduced cross-sections, the NMC proton reduced cross-sections and neutral- and charged-current cross-sections from the H1 experiments from the HERA-II dataset. Datasets are as in Table 1 of Ref. [5]. For each dataset, we compare the theoretical calculations at LO and in the FONLL-A, B and C [100] heavy-quark mass schemes

**Fig. 75:**
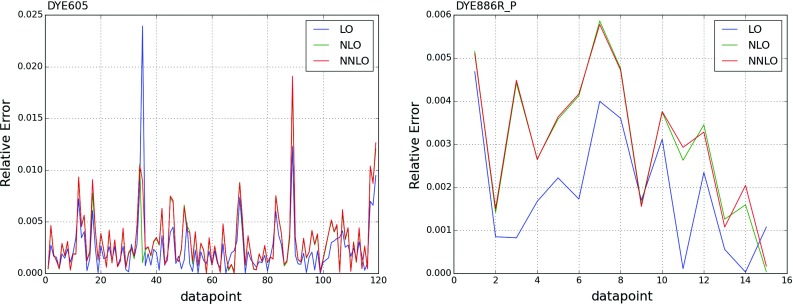
Same as Fig. 74 for fixed-target Drell–Yan cross-sections: results are shown at LO, NLO and NNLO for the E605 *pA* and E866 *pp* cross-section datasets, as given in Table 2 of Ref. [5]

Such a further increase in dataset is likely to require substantial methodological improvements in PDF determination. Also, it is likely to result in a further reduction of PDF uncertainties, thereby requiring better control of theoretical uncertainties. We specifically expect significant progress in two different directions. On the one hand, electroweak corrections, which are now not included, and whose impact is kept under control through kinematic cuts, will have to be included in a more systematic way. On the other hand, theoretical uncertainties due to missing higher-order corrections will also have to be estimated. Indeed, the preliminary estimates presented in Sect. 3.5 suggest that missing higher-order uncertainties on PDFs, currently not included in the PDF uncertainty, are likely to soon become non-negligible, and possibly dominant in some kinematic regions. All of these improvements will be part of a future major PDF release.

## Appendix A: Code development and benchmarking

In all previous NNPDF releases, including NNPDF3.0, PDF evolution and deep-inelastic scattering were computed using the internal FKgenerator code. As discussed in Sect. 2, in NNPDF3.1 PDF evolution is now performed using the public APFEL code. Deep-inelastic scattering and the fixed-target Drell–Yan case are also computed using APFEL. As far as perturbative evolution is concerned, APFEL has been extensively tested against other publicly available codes, such as HOPPET [109] and QCDNUM [171] for the PDF evolution and OpenQCDrad [172] for the calculation of heavy-quark structure functions in the massive scheme.

We have performed an extensive benchmarking of APFEL and FKgenerator, also involving deep-inelastic structure function (as already mentioned in Ref. [23]) and Drell–Yan cross-sections. In the process of this benchmarking, two bugs were found in the FKgenerator implementation of the DIS structure functions (one related to target-mass corrections, the other in the expressions of the $\mathcal {O}(\alpha _s)$ charged-current massive coefficient functions): we checked explicitly that none of them produced an effect on NNPDF3.0 PDFs that could be distinguished from a statistical fluctuation.

Representative results of the benchmarking of deep-inelastic structure functions are shown in Fig. 74, where we show the relative difference between FKgenerator and APFEL implementation, using NNPDF3.0 as input PDF set, for the CHORUS charged-current neutrino–nucleus reduced cross-sections, the NMC proton reduced cross-sections and neutral- and charged-current cross-sections from the H1 experiments from the HERA-II dataset. In each case, we show theoretical predictions calculations at LO and in the FONLL-A, -B and -C general-mass schemes. The two codes are in good agreement, with differences at most being at the 1% level, typically much smaller. This statement holds for all perturbative orders.

The benchmarking of Drell–Yan cross-sections is illustrated in Fig. 75, where we show the relative difference between the FKgenerator and APFEL calculations at LO, NLO and NNLO for the E605 *pd* and E866 *pp* cross-sections, again using NNPDF3.0 as input. Again, differences are at most at the 2% level and typically much smaller. We have traced these residual differences were to the fact that the coverage of the large-*x* region was sub-optimal in the FKgenerator calculation, and is now improved in APFEL thanks to a better choice of input *x* grid.
